# Potent MOR Agonists from 2′-Hydroxy-5,9-dimethyl-*N*-phenethyl Substituted-6,7-benzomorphans and from C8-Hydroxy, Methylene and Methyl Derivatives of *N*-Phenethylnormetazocine

**DOI:** 10.3390/molecules28237709

**Published:** 2023-11-22

**Authors:** Madhurima Das, George W. Ward, Agnieszka Sulima, Dan Luo, Thomas Edward Prisinzano, Gregory H. Imler, Andrew T. Kerr, Arthur E. Jacobson, Kenner C. Rice

**Affiliations:** 1 Drug Design and Synthesis Section, Molecular Targets and Medications Discovery Branch, Intramural Research Program, National Institute on Drug Abuse and the National Institute on Alcohol Abuse and Alcoholism, National Institutes of Health, Department of Health and Human Services, 9800 Medical Center Drive, Bethesda, MD 20892, USA; madhurima.das.91@gmail.com (M.D.); george.ward@fda.hhs.gov (G.W.W.); agnieszka.sulima@nih.gov (A.S.); 2 Department of Pharmaceutical Sciences, College of Pharmacy, University of Kentucky, 789 S. Limestone Street, Lexington, KY 40536, USA; dan.luo@uky.edu (D.L.); prisinzano@uky.edu (T.E.P.); 3 Center for Biomolecular Science and Engineering, Naval Research Laboratory, Washington, DC 20375, USA; greg.imler@gmail.com (G.H.I.); andrew.kerr@nrl.navy.mil (A.T.K.)

**Keywords:** opioid, *N*-phenethyl analogs of 6,7-benzomorphans, *N*-phenethylnormetazocine derivatives, C8-alkyl substituted 6,7-benzomorphans, C8-alkenyl substituted 6,7-benzomorphans, inhibition of forskolin-induced cAMP accumulation assay (cAMP assay)

## Abstract

(−)-5,9-Dimethyl-6,7-benzomorphan (normetazocine) derivatives with a *para*-OH or *ortho*-F substituent in the aromatic ring of the *N*-phenethyl moiety were synthesized and found to have subnanomolar potency at MOR, and both were fully efficacious in vitro. These new compounds, (1*R*,5*R*,9*R*)-6,11-dimethyl-3-(2-fluorophenethyl)-1,2,3,4,5,6-hexahydro-2,6-methanobenzo[d]azocin-8-ol and (1*R*,5*R*,9*R*)-6,11-dimethyl-3-(4-hydroxyphenethyl)-1,2,3,4,5,6-hexahydro-2,6-methanobenzo[d]azocin-8-ol, were more potent than the unsubstituted compound *N*-phenethylnormetazocine and about 30 or 40 times more potent than morphine, respectively. A variety of substituents in the *ortho*, *meta*, or *para* position in the aromatic ring of the *N*-phenethyl moiety were synthesized, 25 of these compounds, and found to have varying effects on potency and efficacy as determined by the forskolin-induced cAMP accumulation assay. The *N*-phenethyl moiety was also modified by increasing chain length to form a *N*-phenylpropyl side chain with and without a *para*-nitro moiety, and by an *N*-cinnamyl side chain. Also, an indole ethylamine normetazocine was synthesized to replace the *N*-phenethylamine side chain in normetazocine. The phenylpropylamine, propenylamine (cinnamyl) and the *para*-nitropropylamine had little or no MOR potency. The indole-ethylamine on the normetazocine nucleus, however, had moderate potency (MOR EC_50_ = 12 nM), and was fully efficacious (%E_max_ = 102%) in the cAMP assay. Retention of the *N*-phenethyl moiety and the addition of alkyl and alkenyl moieties on C8 in (−)-*N*-phenethylnormetazocine gave a C8-methylene derivative that had subnanomolar potency at MOR and a C8-methyl analog that had nanomolar potency. Five C8-substituted compounds were synthesized.

## 1. Introduction

**Figure 1:**
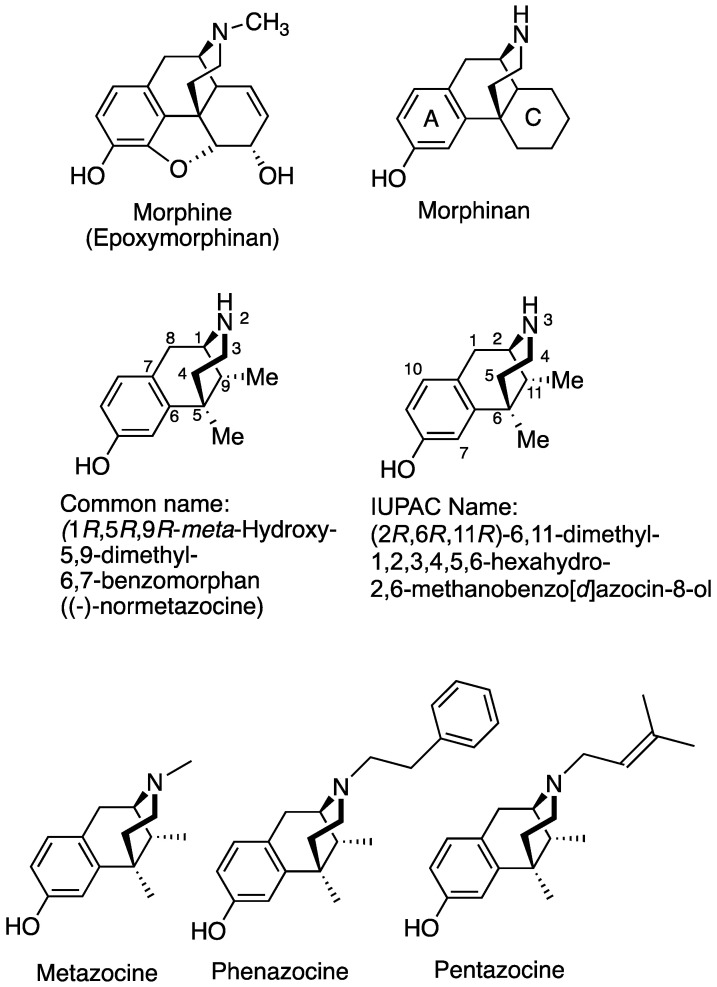
Molecular structures of morphine, morphinan, and the 6,7-benzomorphans-metazocine, phenazocine and pentazocine.

The 6,7-benzomorphans (Figure 1) have been of interest for the past several decades and analgesics based on them have been found to be clinically useful [1,2]. The 6,7-benzomorphan structure is derived from the morphinans. It lacks the morphinan C-ring, making the 6,7-benzomorphans less complex (Figure 1) than the morphinan or epoxymorphinan opioid structures. This structural modification did not necessarily lead to improved analgesics: e.g., a prototypical 6,7-benzomorphan, metazocine, *N*-methyl-5, 9-dimethyl-6,7-benzomorphan, not only acts at the mu opioid receptor (MOR) essentially like morphine, but also has dysphoric and hallucinogenic side-effects attributed to interaction with the kappa opioid receptor (KOR). Several of the derivatives of the 6,7-benzomorphans have been found to act as MOR agonists or antagonists, depending on the N-substituent. The racemic phenazocine (*N*-phenethyl relative of metazocine, Figure 1) was found to have milder side-effects in vivo (e.g., physical dependence, sedation, depression) than equivalent doses of morphine. Pentazocine, the *N*-dimethylallyl relative of metazocine, acts at both MOR and KOR and has been found to be a clinically useful analgesic with milder opioid-like side-effects. It is in clinical use and has been combined with naloxone to lessen its illicit use. The (+)-*N*-allylnormetazocine (SKF 10.047, Alazocine) is the prototypical sigma-1 receptor agonist [3] and does not interact at opioid receptors. Its (−)-enantiomer was found to be a MOR antagonist [4].

A considerable amount of effort has been expended on the synthesis of derivatives of the classical opioids [5], as well as the 6,7-benzomorphans [6,7,8,9,10,11,12], in an attempt to ameliorate their undesirable side-effects, with relatively little success [13].

Various alkyl moieties have been added to the 5,9-positions in the benzomorphan, *cis* and *trans*, and this has influenced the potency of the compound, but the side-effects were those noted for the classical opioids. When the phenolic hydroxyl was converted to a carbonamide function, it was found to have increased potency and, most interestingly, some of those benzomorphans were partial agonists in the [^35^S]GTPgS (GTP) assay [14,15]. Depending on the alkyl substituents at C5 and C9 and the stereochemistry of the molecule, changing the N-substituent has been shown to have a considerable effect on the benzomorphan [12], sometimes improving its potency and, in a few instances, changing its receptor interaction to that of a bifunctional ligand (MOR-σ1) [16].

We have found that substituents on the aromatic ring of the *N*-phenethyl substituent can modify the potency and efficacy of a different type of compound, norhydromorphone, as measured in the inhibition of forskolin-induced cAMP accumulation (cAMP) and [^35^S]GTPgS (GTP) functional assays [17]. The *p*-chlorophenethyl moiety was found to transform norhydromorphone into a bifunctional ligand that had subnanomolar potency at MOR and DOR in the cAMP assay, and that compound did not depress respiration in a rodent assay. We posed the question of whether we could obtain similar results with substituents on the aromatic ring in the *N*-phenethyl moiety of the 6,7-benzomorphans.

Many N-substituents have been synthesized in the 6,7-benzomorphans, including *N*-methyl through decyl [10], heterocyclic derivatives of the *N*-methyl substituent [18], cyanoalkyl, *N*-allyl and *N*-alkynyl [13], *N*-napthyl and quinolyl substituents, *N*-phenylpropanamide [12] and *N*-propyl amides [19]. *N*-Phenethyl-6,7-benzomorphans are very well-known [17,20], but a study of the in vitro effect of substituents on the aromatic ring in the *N*-phenethyl substituent is novel. In this work, a series of (−)-*N*-phenethylnormetazocines were synthesized with electron-withdrawing or -donating substituents on the aromatic ring of the *N*-phenethyl moiety to determine whether they could alter the functional effects of the 6,7-benzomorphans as measured in the cAMP assay and, possibly, convert them to partial agonists, compounds that are not as efficacious as morphine, with the hope that this would lessen their side-effects. Some partial agonists have been noted to have less effect than fully efficacious agonists like heroin or fentanyl on respiratory depression [21,22], a major cause of death due to opioid overdose.

The effect of substituents at C9 in the 5-(3-hydroxy)phenylmorphans, a position comparable to the C8 in normetazocine (Figure 1), has been extensively explored [20,21,22]. Substituents at C9 in the phenylmorphans and C8 in the benzomorphans are relatively close to the tertiary nitrogen atom. Substituents at C8 in 6,7-benzomorphan enantiomers are not well-known, and C8-alkyl and alkenyl-substituted *N*-phenethylnormetazocine enantiomers have not been previously examined. The (−)-*N*-phenethylnormetazocine enantiomers with a C8-methyl or C8 methylene substituent were found to be very potent MOR agonists and were fully efficacious.

## 2. Results and Discussion

### 2.1. Optical Resolution of (±)-Normetazocine

**Scheme 1:**
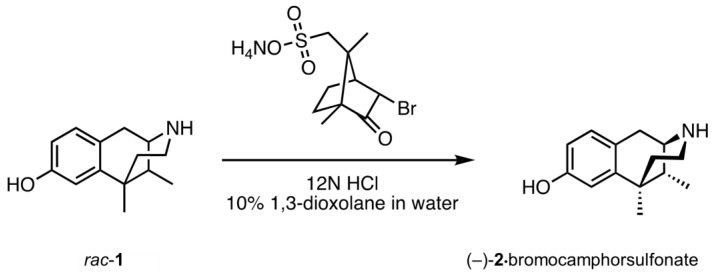
Optical resolution of racemic normetazocine.

Starting with racemic normetazocine (*rac*-**1**, Scheme 1) [2], optical resolution was most easily achieved through treatment with (+)-3-bromo-8-camphorsulphonic acid (bromocamphorsulfonic acid) [23]. This resulted in enantiomerically pure (−)-normetazocine as the bromocamphorsulfonate salt (**2**). The optical purity was determined by NMR using (*R*)-(+)-methylbenzyl isocyanate for chiral derivatization in CDCl_3_/MeOD (1/1).

### 2.2. Substituents on the Aromatic Ring in the N-Phenethyl Side Chain

**Scheme 2:**
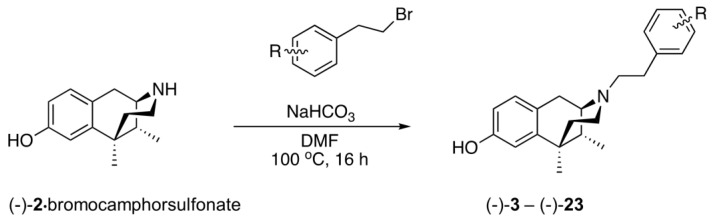
Synthesis of *N*-phenethyl substituted 6,7-benzomorphans.

With the optically resolved (−)-normetazocine in hand, a series of N-substituted analogs were prepared using procedures in the literature (Scheme 2) [17]. The resolved (−)-**2** was used as the bromocamphorsulfonate salt since it was found that the use of the isolated free base resulted in poorer yields. NaHCO_3_ (3 equivalents) was added to the reaction to generate the free base in situ. Heating the reaction at 100 °C overnight in DMF gave the desired N-substituted analogs in good yields. Various substituted *N*-phenethyl bromides and other miscellaneous alkyl bromides were used as alkylating agents.

All of the (−)-*N*-phenethyl-substituted compounds (4-nitro (**3**), 3-nitro (**4**), 2-nitro (**5**), 4-fluoro (**6**), 3-fluoro (**7**), 2-fluoro (**8**), 4-trifluoromethyl (**9**), 3-trifluoromethyl (**10**), 2-trifluoromethyl (**11**), 4-bromo (**12**), 3-bromo (**13**), 2-bromo (**14**), 4-chloro (**15**), 3-chloro (**16**), 2-chloro (**17**), 4-methoxy (**18**), 4-methyl (**19**), 4-hydroxy (**20**), 2,4-dichloro (**21**), and 2,6-dichloro (**22**), and the unsubstituted compound (**23**)), were converted to their HCl salts by dissolving the free base in hot alcohol (isopropanol, ethanol, or methanol) and then adding concentrated hydrochloric acid. The HCl salt crystallized after stirring the solution for a few hours, employing a slow diffusion method with diethyl ether. The HCl salts were recrystallized from hot isopropanol.

### 2.3. Modification of the N-Phenethyl Side Chain

**Scheme 3:**
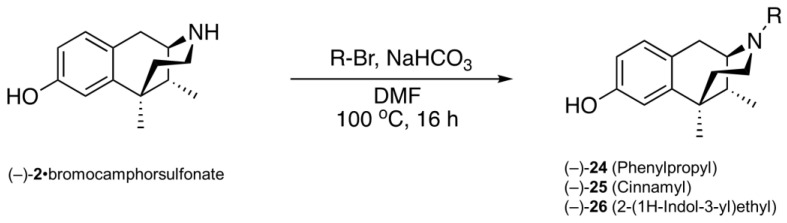
Synthesis of modified (−)-*N*-phenethyl relatives (−)-**24**–(−)-**26**.

Several other types of (−)-N-substituted compounds were synthesized (Scheme 3) and evaluated for their potency and efficacy (Table 1): an *N*-phenylpropyl substituent (−)-**24**, an *N*-cinnamyl substituent (−)-**25**, and an indole (−)-**26**.

**Scheme 4:**
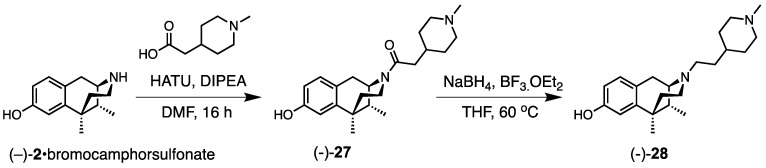
Synthesis of the 1-methyl piperidine analog (−)-**28**.

We then turned to a piperidine analog, a compound bearing a 1-methylpiperidin-4-ylethyl side chain. (−)-Normetazocine, as the bromocamphorsulfonate salt ((−)-**2**•bromocamphorsulfonate), was treated with 2-(1-methylpiperidin-4-yl)acetic acid using HATU as a coupling agent. The resulting amide **27** then underwent a borane reduction [24] to give the desired 1-methylpiperidin-4-ylethyl analog (−)-**28** (Scheme 4).

**Scheme 5:**
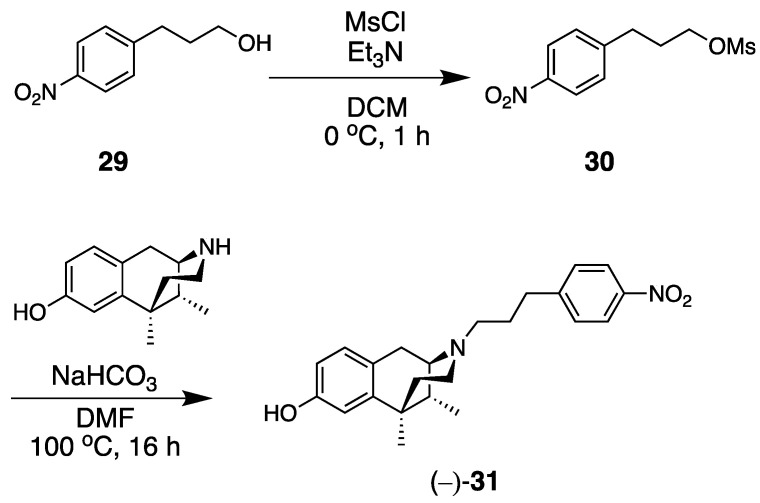
Synthesis of the 4-nitrophenylpropyl substituent (−)-**31**.

A different method was used to synthesize an *N*-(4-nitrophenylpropyl)-substituted analog (−)-**31** because of the unavailability of 4-nitrophenylpropyl bromide. The synthesis was achieved by *O*-mesylating 3-(4-nitrophenyl)propan-1-ol, followed by a nucleophilic substitution with (−)-normetazocine (Scheme 5).

### 2.4. Synthesis of C8-Oxo-Normetazocine

**Scheme 6:**
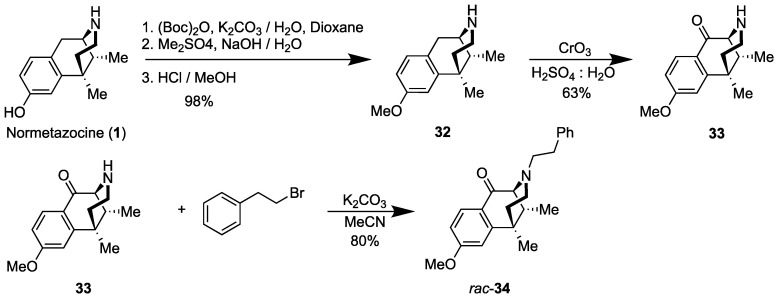
Synthesis of *rac m*-methoxy-8-oxo-*N*-phenethylnormetazocine (*rac*-**34**).

A large-scale synthesis and subsequent optical resolution of a C8-oxo key intermediate (*rac*-2′-methoxy-8-oxo-*N*-phenethylnormetazocine, *rac*-**34**) was undertaken to obtain the starting material for the synthesis of alkenyl and alkyl substituents at that C8 position. The synthesis of the 2′-methoxy racemate *rac*-**34** proceeded according to the literature [25,26] (for the comparable phenol), in good overall yield via a three-step process from normetazocine (**1**) (Scheme 6). Boc protection of the secondary amine in normetazocine, followed by methylation of the phenol and subsequent removal of the Boc group, provided **32** in 98% yield. The C8 position was oxidized using chromium trioxide to form the ketone **33** (63%). Finally, the key intermediate, *rac*-**34**, was synthesized via N-alkylation of the 8-oxo-2′-methoxy-normetazocine (**34**) with phenethyl bromide in high yield (80%). The ketone in **34** was found to be resistant to olefination under Wittig or HWE conditions as well as with oxo-nucleophiles. It did, however, react with organometallics such as alkyl lithiates and Grignard reagents.

### 2.5. Synthesis of the Optical Isomers of C8-oxo m-Methoxy-N-phenethyl-6,7-benzomorphan

**Scheme 7:**
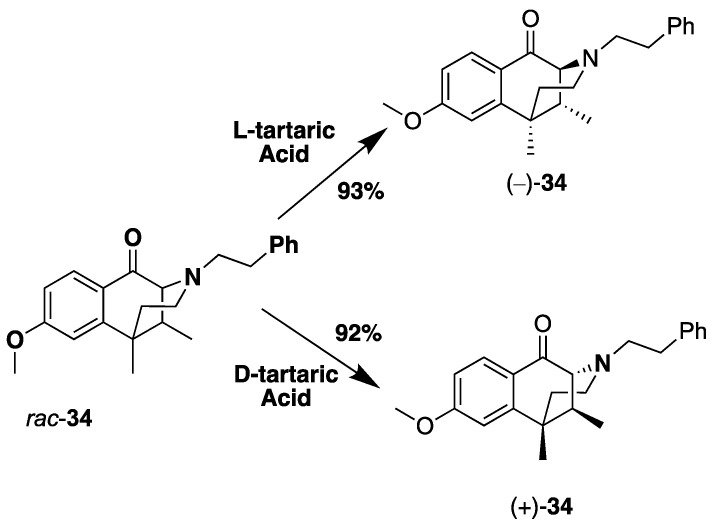
Resolution of *rac*-**34** to provide 1*S*,5*R*,9*R*-(−)-**34** and 1*R*,5*S*,9*S*-(+)-**34**.

While we had successfully resolved normetazocine based on methodology in the literature [27], we preferred to carry the synthesis of the C8-substituted compounds up to *rac*-**34** (Scheme 6) and resolve that compound because that would enable us to run initial exploratory reactions on the available racemic compound. After screening conditions for crystallization, a combination of DMF and acetone was found to be ideal for the optical resolution of *rac*-**34** with (+)-tartaric acid as the chiral acid (Scheme 7). Crystallization occurred after cooling to 0 °C. Conversion to the free base gave, after crystallization, 1*S*,5*R*,9*R* (−)-**34**. Its (+)-enantiomer (1*R*,5*S*,9*S*-(+)-**34**) was obtained after treatment with (−)-tartaric acid of the *rac*-34 base obtained from the residual (+)-tartrate salt of (−)-**34**. These enantiomers had optical rotations of −41.3° and +44.4°, respectively. The resolution of *rac*-**34** was scaled up to provide the needed quantity, and both enantiomers were collected at greater than 90% yield (Scheme 7).

**Figure 2:**
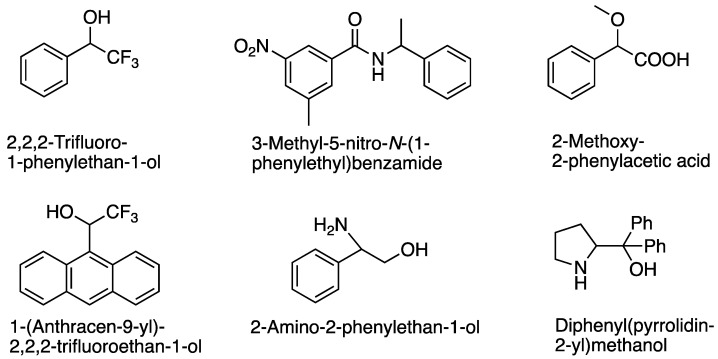
NMR chiral shift reagents.

With the free bases in hand, a way to quantitatively confirm the enantiomeric excess was needed, and several NMR chiral solvating reagents were studied for that purpose (Figure 2).

**Figure 3:**
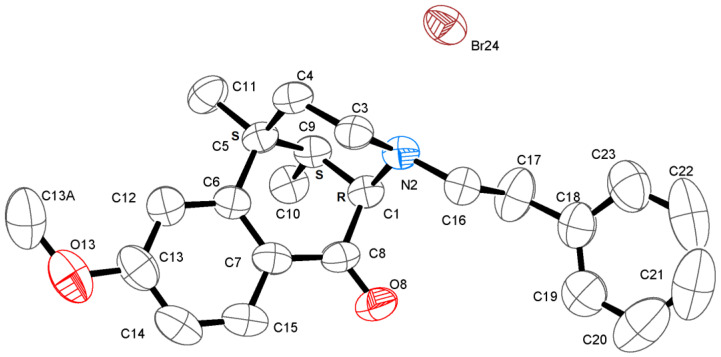
X-ray crystal structure of the HBr salt of (2*R*,6*S*,11*S*)-(+)-**34**.HBr. The ellipsoids are shown at the 50% probability level. The crystal data, atomic coordinates, etc., have been cited in the Supplementary Materials.

The only reagent that was found to be successful for the separation of the enantiomers of **34** so that they could be clearly observed in their NMR spectra was 1-(anthracen-9-yl)-2,2,2-trifluoroethan-1-ol. The two peaks that were most resolved in the ^1^H-NMR spectra and that could be used for determining enantiomeric excess were the methyl doublet and the farthest down-field aryl signal. After a single recrystallization, an enantiomeric excess of >98% was achieved for both enantiomers (see Supplementary Materials for NMR spectra). With a successful resolution method, we needed to determine the absolute configuration of the two enantiomers. Although tartaric acid resolved *rac*-**34** well, it did not provide a proper crystalline form for X-ray spectroscopic determination. A small amount of each enantiomer base was dissolved in acetone and seven drops of concentrated hydrobromic acid added. As the acetone evaporated under ambient pressure, colorless needles formed. The crystals of the HBr salt of (+)-**34** were used for the X-ray crystallographic analysis (Figure 3). The crystal structure analysis confirmed both the overall structure and the absolute configuration for the enantiomeric compounds.

With the enantiopure (−)- and (+)-**34** in hand, we pursued the synthesis of the C8-hydroxy, methylene and methyl compounds.

**Scheme 8:**
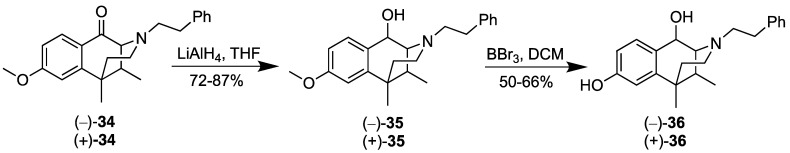
Synthesis of (−)- and (+)-C8-hydroxy benzomorphans.

The C8-hydroxy compounds were synthesized from the resolved *N*-phenethyl C-8 ketones (−)- and (+)-**34** using lithium aluminum hydride to give the phenolic ethers (−)- and (+)-**35** (Scheme 8). O-Demethylation with BBr_3_ gave the desired phenols (−)- and (+)-**36**.

**Scheme 9:**
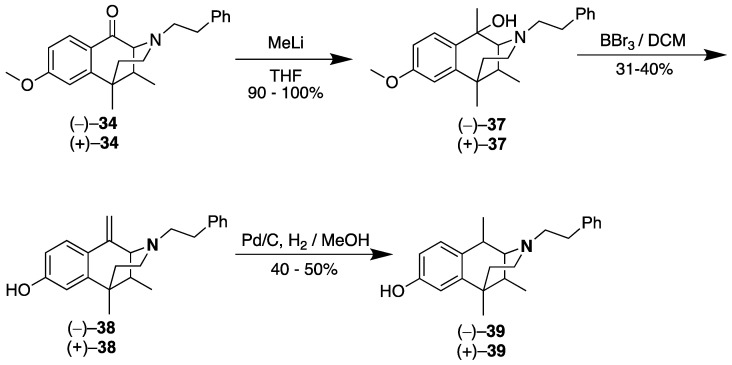
Synthesis of C8-methylene ((−)-**38** and (+)-**38**) and methyl ((−)-**39** and (+)-**39**) compounds.

The reaction of (−)- and (+)-**34** with methyl lithium (Scheme 9) gave the intermediate C8-hydroxymethyl compounds (−)- and (+)-**37** that could be converted to the phenolic C8-vinyl compounds (−)- and (+)-**38** on treatment with BBr_3_. Catalytic reduction of the vinyl compounds gave the desired C8-methyl enantiomers (−)- and (+)-**39.**

### 2.6. Forskolin-Induced cAMP Accumulation Assay for In Vitro Determination of the Potency and Efficacy of the Benzomorphans

Both electron-withdrawing (e.g., NO_2_, (−)-**3**) and -donating (e.g., OH, (−)-**20**) substituents at the *para* position led to highly MOR potent compounds in vitro (Table 1) with EC_50_ values in the subnanomolar range EC_50_ = 0.3 nM and 0.13 nM, respectively. Some of the *ortho*-substituents (e.g., 2-F (−)-**8**) were also extremely potent MOR agonists (EC_50_ = 0.2 nM). The *para*-OH (−)-**20** and the *ortho*-F ((−)-**8**) compounds were among the few synthesized compounds that were more potent than the unsubstituted benzomorphan (−)-**23** (EC_50_ = 0.27 nM). The *meta*-substituted compounds were in general much less potent than the comparable *ortho*- and *para*-analogs, except for the *meta*-fluoro analog (−)-**7** (EC_50_ = 0.6 nM) that was more potent than the *para*-fluoro analog (−)-**6** (EC_50_ = 1.8 nM). Addition of a second chloro substituent 2,4-dichloro, **21** (EC_50_ = 5 nM) or 2,6-dichloro, **22** (EC_50_ = 4 nM) on the aromatic ring reduced the potency of the *ortho* (**17**, EC_50_ = 1.4 nM) or *para* (**15**, EC_50_ = 1.3 nM) monochloro-compound. No clear pattern between agonist potency and electron donating or withdrawal effects was observed. A steric effect due to bulky substituents was also not observed (e.g., the bulky *para*-Br (**12**, EC_50_ = 0.7 nM) and the less bulky *para*-fluoro substituent (**6**, EC_50_ = 1.8 nM)). A second example of that could be seen with the bulky *para*-nitro (**3**) substituted compound having subnanomolar MOR affinity (EC_50_ = 0.3 nM). However, the bulky trifluoromethyl at the *para* position was less potent (**9**, EC_50_ = 2 nM) than compounds with bromo, chloro, or nitro substituents in the *para* position on the aromatic ring, and the dichloro compounds (**21** and **22**) were also less potent than the *para*-monochloro compound **15**. A steric effect that might influence potency at MOR was not clearly observed. Three compounds showed an increase in DOR potency, compared with the unsubstituted *N*-phenethylnormetazocine (**23**). The *para*-nitro, *para*-trifluoromethyl, *para*-bromo, and *para*-chloro substituted compounds (**3**, **9**, **12**, and **15**, respectively) were several-fold more potent than the unsubstituted compound **23**, and these substituents did not modify efficacy—they were all partial DOR agonists. All of the examined compounds were less potent at KOR than the unsubstituted compound **23**. However, **23** had very low efficacy at KOR (EC_50_ = 3.4 nM, %E_max_ = 25%), and most of the compounds with substituents in the aromatic ring in the N-phenethyl side chain had greater efficacy and would, theoretically, be more likely to show the dysphoric and hallucinogenic side-effects known to occur with KOR agonists than the unsubstituted compound **23**.

**Table 1:** Opioid Receptor Activity Measured in the Forskolin-induced cAMP Accumulation Assay ^a^.

			MOR	MOR	DOR	DOR	KOR	KOR
			Agonist	Antagonist ^b^	Agonist	Antagonist ^c^	Agonist	Antagonist ^d^
Compound Number (3–31 (−)-Enantiomers)		Structure	EC_50_ ± SEM (nM) (%E_max_ ± SEM)	IC_50_ ± SEM (nM) (%I_max_ ± SEM)	EC_50_ ± SEM (nM) (%E_max_ ± SEM)	IC_50_ ± SEM (nM) (%I_max_ ± SEM)	EC_50_ ± SEM (nM) (%E_max_ ± SEM)	IC_50_ ± SEM (nM) (%I_max_ ± SEM)
		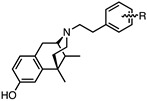 R =						
**3**	**02-0011**	4-NO_2_	0.3 ± 0.05 (101 ± 1%)	N/D	2.7 ± 1.0 (88 ± 5%)	N/D	13 ± 0.5 (46 ± 7%)	N/D
**4**	**02-0015**	3-NO_2_	26 ± 4.0 (71 ± 12%)	N/D	N/D	N/D	N/D	N/D
**5**	**02-0018**	2-NO_2_	2.7 ± 0.4 (101 ± 0.2%)	N/D	37 ± 6 (73 ± 14%)	N/D	16 ± 4 (23 ± 8%)	717 ± 132 (89 ± 5%)
**6**	**02-0006**	4-F	1.8 ± 0.3 (103 ± 0.2%)	N/D	12 ± 3 (73 ± 11%)	N/D	7 ± 3 (34± 6%)	368 ± 92 (71 ± 2%)
**7**	**02-0023**	3-F	0.6 ± 0.1 (100 ± 1%)	N/D	11± 4 (86 ± 5%)	N/D	>10,000	274 ± 83 (87 ± 2%)
**8**	**02-0020**	2-F	0.2 ± 0.1 (101 ± 1%)	N/D	6 ± 2 (87± 5%)	N/D	6 ± 1 (45 ± 7%)	N/D
**9**	**02-0017**	4-CF_3_	2 ± 1 (102 ± 1%)	N/D	3 ± 1 (72 ± 14%)	N/D	23 ± 5 (100 ± 2%)	N/D
**10**	**02-0024**	3-CF_3_	167 ± 72 (63 ± 13%)	N/D	N/D	N/D	N/D	N/D
**11**	**02-0008**	2-CF_3_	6 ± 0.2 (102 ± 1%)	N/D	24 ± 5 (77 ± 9%)	N/D	36 ± 3 (72 ± 6%)	N/D
**12**	**02-0010**	4-Br	0.7 ± 0.2 (102 ± 1%)	N/D	1.1 ± 0.3 (89± 4%)	N/D	5 ± 2 (100 ±1%)	N/D
**13**	**02-0014**	3-Br	18 ± 7 (78 ± 9%)	N/D	110 ± 24 (51 ± 4%)	N/D	>10,000	90 ± 37 (99 ± 2%)
**14**	**02-0021**	2-Br	2 ± 0.4 (101 ± 2%)	N/D	13 ± 2 (89 ± 6%)	N/D	16 ± 2 (80 ± 6%)	N/D
**15**	**02-0009**	4-Cl	1.3 ± 0.4 (103 ± 1%)	N/D	1.2 ± 0.5 (89 ± 6%)	N/D	5 ± 1 (97 ± 1%)	N/D
**16**	**02-0012**	3-Cl	13 ± 2 (95 ± 2%)	N/D	93 ± 25 (64 ± 6%)	N/D	>10,000	111 ± 43 (108 ± 10%)
**17**	**02-0022**	2-Cl	1.4 ± 0.3 (101 ± 1%)	N/D	15 ± 4.0 (87 ± 5%)	N/D	8 ± 1 (57 ±11%)	N/D
**18**	**02-0007**	4-OMe	1.2 ± 0.4 (103 ± 0.3%)	N/D	4 ± 1 (87 ± 6%)	N/D	9 ± 2 (89 ± 3%)	N/D
**19**	**02-0005**	4-Me	1.4 ± 0.4 (103 ± 1%)	N/D	5 ± 2 (87± 5%)	N/D	13 ± 3 (93 ± 3%)	N/D
**20**	**02-0019**	4-OH	0.13 ± 0.02 (102 ± 0.4%)	N/D	5 ± 1 (84 ± 5%)	N/D	8 ± 3 (97 ± 1%)	N/D
**21**	**02-0025**	2,4-dichloro	5 ± 3 (103 ± 1%)	N/D	37 ± 9 (86 ± 12%)	N/D	42 ± 10 (93± 5%)	N/D
**22**	**02-0026**	2,6-dichloro	4 ± 1 (99 ± 2%)	N/D	59 ± 25 (78 ± 11%)	N/D	>10,000	65 ± 24 (87 ± 7%)
**23**	**02-013**	H	0.27 ± 0.1 (101 ± 0.3%)	N/D	4 ± 1 (85 ± 6%)	N/D	3.4 ± 0.4 (25 ± 5%)	281± 94 (81 ± 5%)
		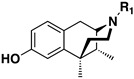						
		*N*-R_1_ =						
**24**	**02-0027**	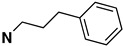	69 ± 13 (100 ± 1%)	N/D	N/D	N/D	N/D	N/D
**25**	**02-0029**	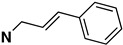	244 ± 58 (101 ± 1%)	N/D	N/D	N/D	N/D	N/D
**26**	**02-0034**	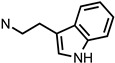	12 ± 1 (102 ± 1%)	N/D	343 ± 35 (91 ± 5%)	N/D	>10,000	>10,000
**28**	**02-0057**	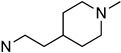	>10,000	>10,000	>10,000	>10,000	0.05 ± 0.02 (15 ± 3%)	>10,000
**31**	**02-0033**	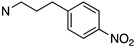	>10,000	>10,000	N/D	N/D	N/D	N/D
		**C8-Substituted**						
(+)-**36**	GW-S-01-73	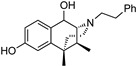	25.2 ± 5.9 (103 ± 0.3%),	N/D	1906 ± 232 (97 ± 4.2%)	N/D	>10,000	N/D
(+)-**38**	GW-S-01-75	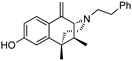	70 ± 15 (102 ± 1%)	N/D	978 ± 374 (92 ± 3.6%)	N/D	1181 ± 46 (53 ± 6.3%)	N/D
(−)-**38**	GW-S-01-76	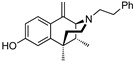	0.23 ± 0.01 (101 ± 0.4%)	N/D	4.9 ± 3.0 (89 ± 2.6%)	N/D	9.5 ± 2.0 (57 ± 7.6%)	N/D
(+)-**39**	GW-01-106	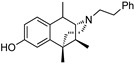	21.7 ± 10.4 (103% ± 1.5%)	N/D	1338 ± 389 (94 ± 1.7%)	N/D	953 ± 120 (37 ± 5.4%)	>10,000
(−)-**39**	GW-01-107	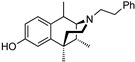	1.2 ± 0.5 (102% ± 1%)	N/D	12.6 ± 3.9 (91± 2.2%)	N/D	20.7 ± 6.1 (38 ± 8.0%)	731 ± 362 (57 ± 5%)
		**Standards**						
		Morphine	5.8 ± 0.3 (102 ± 0.1%)					
		DAMGO	0.3 ± 0.1 (103 ± 1%)					
		U50488H					0.3 ± 0.03 (100 ± 0.3%)	
		SNC80			1.7 ± 0.2 (79 ± 2%)			
		Naltrexone	2.1 ± 1.2 (30 ± 6%)	11 ± 1 (104 ± 1%)	>10,000	295 ± 48 (99 ± 1%)	0.6 ± 0.3 (57 ± 7%)	6 ± 1 (41 ± 7%)
		nor-BNI						2.3 ± 0.3 (102 ± 1%)

^a^ Inhibition of forskolin-induced cAMP accumulation; cAMP Hunter^TM^ Chinese hamster ovary cells (CHO-K1) that express human μ-opioid receptor (OPRM1), human κ-opioid receptor (OPRK1), and human δ-opioid receptor (OPRD1) were used for the forskolin-induced cAMP accumulation assay to determine potency and efficacy of the compounds following the previously established methods [28]; to determine % efficacy in forskolin-induced cAMP assays, data were blank subtracted with the vehicle control, followed by normalization to the forskolin control. Data were then analyzed in GraphPad Prism 8 (GraphPad, LaJolla, CA, USA) using nonlinear regression; values are expressed as the mean ± SEM of at least three independent experiments; N/D = not determined (MOR antagonist activity was not determined when a compound had potent, fully efficacious MOR agonist activity; DOR and KOR agonist activity were not determined if the MOR agonist activity was less than 20 nM). ^b^ MOR antagonist potency (IC_50_) determined versus EC_90_ of fentanyl; degree of antagonism (I_max_) normalized to naltrexone. ^c^ DOR antagonist potency (IC_50_) determined versus EC_50_ of SNC80; degree of antagonism (I_max_) normalized to naltrexone. ^d^ KOR antagonist potency (IC_50_) determined versus EC_90_ of U50488H; degree of antagonism (Imax) normalized to *nor*-BNI.

The *N*-phenethyl side-chain-modified benzomorphans (−)-**24,** (−)-**25,** (−)-**28** and (−)-**31** (Table 1) were found to have little or no potency in vitro at MOR. The indole (−)-**26**, however, was fully efficacious and had moderate MOR agonist activity (EC_50_ = 12 nM, %E_max_ = 102%). Apparently, the addition of a single methylene in the side chain of the *N*-phenethyl moiety, to give an *N*-phenylpropyl derivative (−)-**24**, changed the highly MOR potent *N*-phenethyl compound (−)-**23** (EC_50_ = 0.27, %E_max_ = 101%) into a relatively inactive compound (−)-**24** (EC_50_ = 69 nM). Introduction of a double-bond in the side chain to give the cinnamyl derivative (−)-**25**, decreased the potency even more (EC_50_ = 244 nM). The addition of a *para*-nitro moiety to the *N*-phenylpropyl side chain gave a totally inactive compound (−)-**31** (MOR agonist EC_50_ ≥ 10,000 nM), in contrast with the *para*-nitro *N*-phenethyl compound (−)-**3** with a MOR EC_50_ = 0.3 nM. The saturated 1-methylpiperidine substituent (−)-**28** was also inactive at MOR, but showed high agonist potency at KOR, although with very little efficacy (EC_50_ = 0.05 nM, %E_max_ = 15%).

All the C8 compounds were fully efficacious. As expected, the (+)-C8-compounds (+)-**36**, (+)-**38**, and (+)-**39** were much less potent than the levorotatory compounds (−)-**38** and (−)-**39** at MOR. This was expected, since dextrorotatory benzomorphans are usually much less active than their levorotatory relatives. Two (+)-compounds, the C8-hydroxy and methyl derivatives (+)-**36** and (+)-**39**, had moderate MOR potency (EC_50_ = 25.2 and 21.7 nM, respectively) and the (+)-methylene compound (+)-**38** was considerably less potent (EC_50_ = 70 nM). The methylene compound (−)-**38** was found to have MOR subnanomolar potency (EC_50_ = 0.23 nM); it was about 25 times more potent than morphine. The C8-methyl compound (−)-**39** was also quite potent, with an EC_50_ = 1.2 nM. Both the C8-methylene ((-)-**38**) and methyl ((-)-**39**) compounds had moderate potency at DOR, and neither was as potent as the unsubstituted compound **23** at DOR or KOR. Both compounds had nearly full efficacy at DOR, and both had little efficacy at KOR.

## 3. Materials and Methods

### 3.1. General Information

All reactions were performed in oven-dried glassware under an argon atmosphere unless otherwise noted. Proton (^1^H NMR) and carbon (^13^C NMR) spectra were recorded on a Varian Gemini-400 spectrometer at 400 MHz for ^1^H NMR and 101 MHz for ^13^C NMR. Mass spectra (HRMS) were recorded on a Waters (Mitford, MA, USA) Xevo-G X5 QTof. The optical rotation data were obtained on a PerkinElmer polarimeter model 341, and melting points were obtained using a Thomas-Hoover melting point apparatus. Thin layer chromatography (TLC) was performed on a 250 mm Analtech GHLF. Visualization was accomplished under UV or by staining in an iodine chamber. Flash column chromatography was performed with Fluka silica gel 60 (mesh 220−400). Elemental analyses were performed by Robertson Microlit Laboratories, Ledgewood, NJ, USA.

### 3.2. Synthesis

Optical resolution of *rac*-normetazocine (*rac*-**1**). *rac*-Normetazocine was optically resolved using a procedure in the literature [23] to obtain (−)-normetazocine (−)-**1**. *rac*-Normetazocine (*rac*-**1**, 10.6 g, 1 equiv, 48.8 mmol) was suspended in H_2_O (56 mL). Then, 37% HCl (1.78 g, 4.06 mL, 12 molar, 1 equiv, 48.8 mmol) was added to the solution, followed by (+)-3-bromo-8-camphorsulfonic acid ammonium salt (16.0 g, 1 equiv, 48.8 mmol) and 1,3-dioxolane (5.6 mL). The solution was heated to 100 °C until all of the solids dissolved. The solution was allowed to cool to room temperature and stirred for 1 h. The crystals that formed were filtered and washed with 10% 1,3-dioxolane in H_2_O. The salt was recrystallized 2x from 12% 1,3-dioxolane in H_2_O and dried under high vacuum at 60 °C overnight. NMR studies using (*R*)-(+)-methylbenzyl isocyanate in CDCl_3_:CD_3_OD (1:1) indicated >99% optical purity.

General procedure for N-alkylation of (−)-normetazocine. Optically resolved (−)-2•bromocamphorsulfonate salt (1 equiv, 1.89 mmol), sodium bicarbonate (3 equiv, 3.69 mmol) and the alkyl bromide (1.5 equiv,1.84 mmol) were combined in anhydrous DMF (15 mL). The reaction was stirred at 100 °C for 20 h. The reaction was cooled to room temperature, and H_2_O (20 mL) was added. The mixture was transferred to a separatory funnel and extracted with CHCl_3_ (3 × 20 mL). The organic layers were combined and washed with H_2_O (5 × 10 mL) and brine (20 mL). The organic layer was dried over anhydrous Na_2_SO_4_ and filtered. The solvent was removed, and the residual material was purified by flash chromatography (gradient 1–10% MeOH/NH_4_OH in CHCl_3_).

*(1R,5R,9R)-6,11-Dimethyl-3-(4-nitrophenethyl)-1,2,3,4,5,6-hexahydro-2,6-methanobenzo[d]azocin-8-ol* ((−)-**3**). General procedure was used, and the alkylation was achieved with 4-nitrophenethyl bromide. The product was obtained as a pale-yellow solid (300 mg, 67%), mp (HCl salt) 273–274 °C (dec). ^1^H NMR (400 MHz; CD_3_OD): δ 7.43–7.40 (m, 2H), 7.17–7.14 (m, 2H), 6.89 (d, *J* = 8.3 Hz, 1H), 6.69 (d, *J* = 2.5 Hz, 1H), 6.56 (dd, *J* = 8.2, 2.6 Hz, 1H), 3.02 (dd, *J* = 5.5, 3.1 Hz, 1H), 2.92 (d, *J* = 18.3 Hz, 1H), 2.80–2.74 (m, 3H), 2.72–2.61 (m, 3H), 2.13 (td, *J* = 12.4, 3.2 Hz, 1H), 1.88 (dd, *J* = 7.0, 3.0 Hz, 1H), 1.81 (td, *J* = 12.9, 4.7 Hz, 1H), 1.34 (s, 3H), 1.32 (t, *J* = 2.5 Hz, 1H), 0.87 (d, *J* = 7.0 Hz, 3H). ^13^C NMR (101 MHz; CD_3_OD): δ 155.4, 148.4, 146.5, 142.2, 129.4, 127.7, 126.7, 123.0, 112.7, 111.6, 57.6, 55.7, 45.5, 41.5, 41.2, 35.9, 33.3, 24.4, 22.8, 13.0. HRMS (ES+) Calcd. For C_22_H_27_N_2_O_3_, 367.2022 (M+H)^+^; found, 367.2016. [α]^20^_D_ –94.8° (*c* 1.17, CHCl_3_/MeOH (9/1)). Anal. Calcd. For C_22_H_27_ClN_2_O_3_ • 0.15 C_3_H_8_O: C, 65.46; H, 6.90; N, 6.80. Found: C, 65.60; H, 6.94; N, 6.66.
*(1R,5R,9R)-6,11-Dimethyl-3-(3-nitrophenethyl)-1,2,3,4,5,6-hexahydro-2,6-methanobenzo[d]azocin-8-ol* ((−)-**4**). General procedure was used, and the alkylation was achieved with 3-nitrophenethyl bromide. The product was obtained as a pale-yellow solid (276 mg, 81%), mp (HCl salt) 259–262 °C (dec). ^1^H NMR (400 MHz; CD*3*OD): δ 8.16 (t, *J* = 1.8 Hz, 1H), 8.10–8.07 (m, 1H), 7.67 (d, *J* = 7.7 Hz, 1H), 7.54 (t, *J* = 7.9 Hz, 1H), 6.91 (d, *J* = 8.3 Hz, 1H), 6.70 (d, *J* = 2.5 Hz, 1H), 6.57 (dd, *J* = 8.2, 2.6 Hz, 1H), 3.04 (dd, *J* = 5.5, 3.1 Hz, 1H), 2.95 (dt, *J* = 10.4, 4.2 Hz, 3H), 2.89–2.81 (m, 1H), 2.78–2.64 (m, 3H), 2.18 (td, *J* = 12.4, 3.2 Hz, 1H), 1.89 (dd, *J* = 7.1, 3.0 Hz, 1H), 1.83 (td, *J* = 12.9, 4.7 Hz, 1H), 1.36 (s, 3H), 1.32 (d, *J* = 20.7 Hz, 1H), 0.88 (d, *J* = 7.0 Hz, 3H). ^13^C NMR (101 MHz; CD_3_OD): δ 155.4, 148.3, 142.5, 142.2, 134.9, 129.1, 127.7, 126.6, 123.1, 120.7, 112.8, 111.6, 57.6, 55.8, 45.5, 41.5, 41.2, 35.9, 32.9, 24.5, 22.8, 13.0. HRMS (ES+) Calcd. For C_22_H_27_N_2_O_3_, 367.2022 (M+H)^+^; found, 367.2022[α]^20^_D_ –94.5° (*c* 1.1, CHCl_3_/MeOH (9/1)). Anal. Calcd. For C_22_H_27_ClN_2_O_3_ • 0.25 C_3_H_8_O: C, 65.38; H, 6.99; N, 6.70. Found: C, 65.19; H, 6.80; N, 6.82.
*(1R,5R,9R)-6,11-Dimethyl-3-(2-nitrophenethyl)-1,2,3,4,5,6-hexahydro-2,6-methanobenzo[d]azocin-8-ol* ((-)-**5**). General procedure was used, and the alkylation was achieved with 2-nitrophenethyl bromide. The product was obtained as a pale-yellow solid (265 mg, 76%), mp (HCl salt) 237–239 °C (dec). ^1^H NMR (400 MHz; CD_3_OD): δ 7.89 (dd, *J* = 8.2, 1.1 Hz, 1H), 7.59 (td, *J* = 7.5, 1.2 Hz, 1H), 7.47 (dd, *J* = 7.7, 1.2 Hz, 1H), 7.44–7.40 (m, 1H), 6.88 (d, *J* = 8.3 Hz, 1H), 6.68 (d, *J* = 2.5 Hz, 1H), 6.55 (dd, *J* = 8.2, 2.6 Hz, 1H), 3.13 (ddd, *J* = 12.5, 10.5, 5.9 Hz, 1H), 3.03–2.94 (m, 2H), 2.91 (d, *J* = 18.4 Hz, 1H), 2.86–2.79 (m, 1H), 2.71–2.64 (m, 2H), 2.59 (dd, *J* = 11.9, 3.1 Hz, 1H), 2.14 (td, *J* = 12.4, 3.2 Hz, 1H), 1.87 (dd, *J* = 7.1, 3.0 Hz, 1H), 1.81 (td, *J* = 12.8, 4.7 Hz, 1H), 1.33 (s, 3H), 1.29 (t, *J* = 2.4 Hz, 1H), 0.84 (d, *J* = 7.0 Hz, 3H). ^13^C NMR (101 MHz; CD_3_OD): δ 155.3, 149.7, 142.3, 134.7, 132.7, 132.3, 127.7, 127.2, 126.8, 124.1, 112.7, 111.6, 57.7, 55.6, 45.6, 41.4, 41.0, 35.9, 30.5, 24.5, 23.1, 13.1. HRMS (ES+) Calcd. For C_22_H_27_N_2_O_3_, 367.2022 (M+H)^+^; found, 367.2020. [α]^20^_D_ –87.8° (*c* 1.4, CHCl_3_/MeOH (9/1)). Anal. Calcd. For C_22_H_27_ClN_2_O_3_ • 0.3 H_2_O • 0.5 C_3_H_8_O: C, 64.39; H, 7.27; N, 6.39. Found: C, 64.34; H, 7.32; N, 6.42.
*(2R,6R,11R)-6,11-Dimethyl-3-(4-fluorophenethyl)-1,2,3,4,5,6-hexahydro-2,6-methanobenzo[d]azocin-8-ol* ((−)-**6**). General procedure was used, and the alkylation was achieved with 4-fluorophenethyl bromide. The product was obtained as a pale-yellow solid (500 mg, 83%), mp (HCl salt) 303–304 °C(dec). ^1^H-NMR (400 MHz; CD_3_OD): δ 7.23–7.20 (m, 2H), 7.00–6.95 (m, 2H), 6.88 (d, *J* = 8.3 Hz, 1H), 6.67 (d, *J* = 2.5 Hz, 1H), 6.54 (dd, *J* = 8.2, 2.6 Hz, 1H), 3.02 (dd, *J* = 5.6, 3.2 Hz, 1H), 2.91 (d, *J* = 18.4 Hz, 1H), 2.80–2.59 (m, 6H), 2.12 (td, *J* = 12.4, 3.2 Hz, 1H), 1.87 (dd, *J* = 7.0, 3.0 Hz, 1H), 1.80 (td, *J* = 12.9, 4.7 Hz, 1H), 1.33 (s, 3H), 1.30 (t, *J* = 2.3 Hz, 1H), 0.85 (d, *J* = 7.0 Hz, 3H). ^13^C NMR (101 MHz; CD_3_OD): δ 160.2 (2), 155.4, 142.2, 135.90 (2), 129.84 (2), 127.7, 126.6, 114.5 (2), 112.8, 111.6, 57.3, 56.6, 47.87, 47.83, 47.5, 45.6, 41.4, 41.1, 35.9, 32.5, 24.5, 22.5, 13.1. HRMS (ES^+^) Calcd. For C_22_H_27_FNO, 340.2077 (M+H)^+^; found, 340.2075[α]^20^_D_ –109.7° (*c* 1.0, CHCl_3_/MeOH (9/1)). Anal. Calcd. For C_22_H_27_ClFNO: C, 70.29; H, 7.24; N, 3.73. Found: C, 70.55; H, 7.23; N, 3.71.
*(1R,5R,9R)-6,11-Dimethyl-3-(3-fluorophenethyl)-1,2,3,4,5,6-hexahydro-2,6-methanobenzo[d]azocin-8-ol* ((−)-**7**). General procedure was used, and the alkylation was achieved with 3-fluorophenethyl bromide. The product was obtained as a pale-yellow solid (200 mg, 64%), mp (HCl salt) 268–270 °C (dec). ^1^H NMR (400 MHz; CD_3_OD): δ 7.26 (td, *J* = 7.9, 6.1 Hz, 1H), 7.03 (d, *J* = 7.7 Hz, 1H), 6.97 (dt, *J* = 10.1, 2.0 Hz, 1H), 6.92–6.88 (m, 2H), 6.67 (d, *J* = 2.5 Hz, 1H), 6.55 (dd, *J* = 8.2, 2.6 Hz, 1H), 3.07 (dd, *J* = 5.6, 3.1 Hz, 1H), 2.92 (d, *J* = 18.4 Hz, 1H), 2.85–2.78 (m, 3H), 2.75–2.63 (m, 3H), 2.17 (td, *J* = 12.5, 3.2 Hz, 1H), 1.89 (tt, *J* = 7.0, 3.5 Hz, 1H), 1.81 (td, *J* = 13.0, 4.7 Hz, 1H), 1.35 (dd, *J* = 3.1, 2.1 Hz, 1H), 1.33 (s, 3H), 0.86 (d, *J* = 7.0 Hz, 3H). ^13^C NMR (101 MHz; CD_3_OD): δ 162.9 (2), 155.5, 142.6 (2), 142.0, 129.7 (2), 127.7, 126.3, 124.1 (2), 115.0 (2), 112.9, 112.4 (2), 111.6, 57.6, 56.0, 45.6, 41.2, 40.9, 35.8, 32.9, 24.4, 22.6, 13.0. HRMS (ES^+^) Calcd. For C_22_H_27_FNO, (M+H)^+^ 340.2077; found, 340.2071[α]^20^_D_ –99.1° (*c* 0.97, CHCl_3_/MeOH (9/1)). Anal. Calcd. For C_22_H_27_ClFNO: C, 70.13; H, 7.25; N, 3.72. Found: C, 69.90; H, 6.96; N, 3.75.
*(1R,5R,9R)-6,11-Dimethyl-3-(2-fluorophenethyl)-1,2,3,4,5,6-hexahydro-2,6-methanobenzo[d]azocin-8-ol* ((−)-**8**). General procedure was used, and the alkylation was achieved with 2-fluorophenethyl bromide. The product was obtained as a pale-yellow solid (200 mg, 62%), mp (HCl salt) 263–265 °C (dec). ^1^H NMR (400 MHz; CD_3_OD): δ 7.25 (td, *J* = 7.6, 1.6 Hz, 1H), 7.19 (ddd, *J* = 7.6, 5.5, 2.0 Hz, 1H), 7.09–7.05 (m, 1H), 7.04–6.99 (m, 1H), 6.87 (d, *J* = 8.3 Hz, 1H), 6.67 (d, *J* = 2.5 Hz, 1H), 6.54 (dd, *J* = 8.2, 2.5 Hz, 1H), 3.05 (dd, *J* = 5.5, 3.1 Hz, 1H), 2.93–2.84 (m, 2H), 2.78 (ddt, *J* = 19.8, 12.3, 6.4 Hz, 2H), 2.70–2.67 (m, 1H), 2.65–2.61 (m, 2H), 2.13 (td, *J* = 12.4, 3.2 Hz, 1H), 1.88 (dd, *J* = 7.0, 3.0 Hz, 1H), 1.81 (td, *J* = 12.9, 4.7 Hz, 1H), 1.32 (s, 3H), 1.30 (t, *J* = 2.4 Hz, 1H), 0.85 (d, *J* = 7.0 Hz, 3H). ^13^C NMR (101 MHz; CD_3_OD): δ 161.1 (2), 155.4, 142.1, 130.7 (2), 127.8 (2), 127.7, 126.5 (2), 126.4, 123.9 (2), 114.8, 114.6, 112.8, 111.6, 57.4, 54.9, 45.6, 41.3, 40.9, 35.8, 26.59, 26.57, 24.4, 22.6, 13.1. HRMS (ES^+^) Calcd. For C_22_H_27_FNO, 340.2077 (M+H)^+^; found, 340.2082[α]^20^_D_ –107.1° (*c* 1.0, CHCl_3_/MeOH (9/1)). Anal. Calcd. For C_22_H_27_ClFNO: C, 70.29; H, 7.24; N, 3.73. Found: C, 70.13; H, 6.94; N, 3.75.
*(1R,5R,9R)-6,11-Dimethyl-3-(4-trifluoromethylphenethyl)-1,2,3,4,5,6-hexahydro-2,6-methanobenzo[d]azocin-8-ol* ((−)-**9**). General procedure was used, and the alkylation was achieved with 4-trifluoromethylphenethyl bromide. The product was obtained as a pale-yellow solid (260 mg, 59%), mp (HCl salt) 283–285 °C (dec). ^1^H NMR (400 MHz; CD_3_OD): δ 7.58 (d, *J* = 8.1 Hz, 2H), 7.43 (d, *J* = 8.1 Hz, 2H), 6.90 (d, *J* = 8.3 Hz, 1H), 6.70 (d, *J* = 2.5 Hz, 1H), 6.57 (dd, *J* = 8.2, 2.6 Hz, 1H), 3.04 (dd, *J* = 5.6, 3.1 Hz, 1H), 2.96–2.87 (m, 3H), 2.86–2.77 (m, 1H), 2.74–2.62 (m, 3H), 2.15 (td, *J* = 12.4, 3.2 Hz, 1H), 1.90 (td, *J* = 7.0, 3.0 Hz, 1H), 1.83 (td, *J* = 12.8, 4.7 Hz, 1H), 1.35 (s, 3H), 1.33 (t, *J* = 2.5 Hz, 1H), 0.88 (d, *J* = 7.0 Hz, 3H). ^13^C NMR (101 MHz, CD_3_OD): d 155.4, 144.8, 142.2, 129.0, 128.0 (4), 127.5, 126.6, 124.8 (4), 124.4 (4), 112.8, 111.6, 57.4, 56.0, 45.5, 41.4, 41.1, 35.9, 33.2, 24.5, 22.6, 13.1. HRMS (ES^+^) Calcd. For C_23_H_27_NOF_3_, 390.2045 (M+H)^+^; found, 390.2043[α]^20^_D_ –87.5° (*c* 1.0, CHCl_3_/MeOH (9/1)). Anal. Calcd. For C_23_H_27_ClF_3_NO • 0.05 C_3_H_8_O: C, 64.83; H, 6.44; N, 3.27. Found: C, 64.84; H, 6.30; N, 3.10.
*(1R,5R,9R)-6,11-Dimethyl-3-(3-trifluoromethylphenethyl)-1,2,3,4,5,6-hexahydro-2,6-methanobenzo[d]azocin-8-ol* ((−)-**10**). General procedure was used, and the alkylation was achieved with 3-trifluoromethylphenethyl bromide. The product was obtained as a pale-yellow solid (280 mg, 77%), mp (HCl salt) 282.2–284.0 °C (dec). ^1^H NMR (400 MHz; CD_3_OD): δ 7.53 (s, 1H), 7.49–7.42 (m, 3H), 6.87 (d, *J* = 8.3 Hz, 1H), 6.67 (d, *J* = 2.5 Hz, 1H), 6.54 (dd, *J* = 8.2, 2.5 Hz, 1H), 3.00 (dd, *J* = 5.3, 3.1 Hz, 1H), 2.92–2.84 (m, 3H), 2.80–2.75 (m, 1H), 2.73–2.58 (m, 3H), 2.13 (td, *J* = 12.4, 3.1 Hz, 1H), 1.86 (dd, *J* = 7.0, 2.9 Hz, 1H), 1.79 (td, *J* = 12.9, 4.7 Hz, 1H), 1.32 (s, 3H), 1.30 (s, 1H), 0.84 (d, *J* = 7.0 Hz, 3H). ^13^C NMR (101 MHz; CD_3_OD): δ 155.4, 142.2, 141.5, 132.2, 130.2 (4), 128.8, 127.7, 126.7, 125.0 (4), 124.3 (4), 122.5 (4), 112.8, 111.6, 57.5, 56.1, 45.5, 41.5, 41.1, 35.9, 33.1, 24.5, 22.7, 13.1. HRMS (ES+) Calcd. For C_23_H_27_F_3_NO, (M+H)^+^ 390.2045; found, 390.2040[α]^20^_D_ –91.0° (*c* 1.2, CHCl_3_/MeOH (9/1)). Anal. Calcd. For C_23_H_27_ClF_3_NO: C, 64.86; H, 6.39; N, 3.29. Found: C, 64.64; H, 6.12; N, 3.23.
*(1R,5R,9R)-6,11-Dimethyl-3-(2-trifluoromethylphenethyl)-1,2,3,4,5,6-hexahydro-2,6-methanobenzo[d]azocin-8-ol* ((−)-**11**). General procedure was used, and the alkylation was achieved with 2-trifluoromethylphenethyl bromide. The product was obtained as a pale-yellow solid (100 mg, 14%), mp (HCl salt) 215–217 °C. ^1^H NMR (400 MHz; CD_3_OD): δ 7.63 (d, *J* = 7.9 Hz, 1H), 7.53 (t, *J* = 7.5 Hz, 1H), 7.44 (d, *J* = 7.6 Hz, 1H), 7.35 (t, *J* = 7.6 Hz, 1H), 6.87 (d, *J* = 8.3 Hz, 1H), 6.67 (d, *J* = 2.5 Hz, 1H), 6.54 (dd, *J* = 8.2, 2.5 Hz, 1H), 3.02–2.94 (m, 3H), 2.89 (d, *J* = 18.3 Hz, 1H), 2.77 (td, *J* = 11.6, 5.9 Hz, 1H), 2.70–2.61 (m, 3H), 2.14 (td, *J* = 12.4, 3.1 Hz, 1H), 1.89 (dd, *J* = 7.0, 2.9 Hz, 1H), 1.82 (td, *J* = 12.9, 4.7 Hz, 1H), 1.33 (s, 3H), 1.29 (d, *J* = 18.1 Hz, 1H), 0.86 (d, *J* = 7.0 Hz, 3H). ^13^C NMR (101 MHz; CD_3_OD): δ 155.4, 142.2, 138.5, 131.9, 131.6, 128.2, 127.7, 126.6, 126.5 (4), 126.21, 125.4 (4), 112.8, 111.6, 57.7, 56.5, 48.2, 47.8, 46.9, 45.6, 41.4, 41.0, 35.9, 30.3, 24.5, 22.8, 13.1. HRMS (ES^+^) Calcd. For C_23_H_27_F_3_NO_2_, 390.2045 (M+H)^+^; found, 390.2050[α]^20^_D_ –109.8° (*c* 1.0, CHCl_3_/MeOH (9/1)). Anal. Calcd. For C_23_H_27_ClF_3_NO_2_ • 0.6 C_3_H_8_O: C, 64.48; H, 6.94; N, 3.03. Found: C, 64.51; H, 6.95; N, 3.03.
*(1R,5R,9R)-6,11-Dimethyl-3-(4-bromophenethyl)-1,2,3,4,5,6-hexahydro-2,6-methanobenzo[d]azocin-8-ol* ((−)-**12**). General procedure was used, and the alkylation was achieved with 4-bromophenethyl bromide. The product was obtained as a pale-yellow solid (311 mg, 63%), mp (HCl salt) 292–293 °C (dec). ^1^H NMR (400 MHz; CD_3_OD): δ 7.43–7.40 (m, 2H), 7.17–7.14 (m, 2H), 6.89 (d, *J* = 8.3 Hz, 1H), 6.69 (d, *J* = 2.5 Hz, 1H), 6.56 (dd, *J* = 8.2, 2.6 Hz, 1H), 3.02 (dd, *J* = 5.5, 3.1 Hz, 1H), 2.92 (d, *J* = 18.3 Hz, 1H), 2.80–2.74 (m, 3H), 2.72–2.61 (m, 3H), 2.13 (td, *J* = 12.4, 3.2 Hz, 1H), 1.88 (dd, *J* = 7.0, 3.0 Hz, 1H), 1.81 (td, *J* = 12.9, 4.7 Hz, 1H), 1.34 (s, 3H), 1.32 (t, *J* = 2.5 Hz, 1H), 0.87 (d, *J* = 7.0 Hz, 3H). ^13^C NMR (101 MHz; CD_3_OD): δ 155.4, 142.2, 139.3, 131.1, 130.3, 127.7, 126.6, 119.4, 112.8, 111.6, 57.4, 56.2, 45.6, 41.4, 41.1, 35.9, 32.7, 24.4, 22.6, 13.1. HRMS (ES^+^) Calcd. For C_22_H_27_BrNO, 400.1276 (M+H)^+^; found, 400.1271[α]^20^_D_ –105.5° (*c* 1.0, CHCl_3_/MeOH, (9/1)). Anal. Calcd. For C_22_H_27_BrClNO • 0.45 C_3_H_8_O: C, 60.46; H, 6.65; N, 3.02. Found: C, 60.40; H, 6.58; N, 2.96.
*(1R,5R,9R)-6,11-Dimethyl-3-(3-bromophenethyl)-1,2,3,4,5,6-hexahydro-2,6-methanobenzo[d]azocin-8-ol* ((−)-**13**). General procedure was used, and the alkylation was achieved with 3-bromophenethyl bromide. The product was obtained as a pale-yellow solid (276 mg, 81%), mp (HCl salt) 255–257 °C (dec). ^1^H NMR (400 MHz; CD_3_OD): δ 7.42 (s, 1H), 7.34 (dt, *J* = 6.5, 2.3 Hz, 1H), 7.21–7.19 (m, 2H), 6.90 (d, *J* = 8.3 Hz, 1H), 6.69 (d, *J* = 2.5 Hz, 1H), 6.56 (dd, *J* = 8.2, 2.5 Hz, 1H), 3.02 (dd, *J* = 5.5, 3.1 Hz, 1H), 2.92 (d, *J* = 18.3 Hz, 1H), 2.81–2.75 (m, 3H), 2.72–2.60 (m, 3H), 2.14 (td, *J* = 12.4, 3.2 Hz, 1H), 1.88 (dd, *J* = 7.0, 3.0 Hz, 1H), 1.82 (dt, *J* = 12.9, 6.4 Hz, 1H), 1.34 (s, 3H), 1.32 (t, *J* = 2.4 Hz, 1H), 0.87 (d, *J* = 7.0 Hz, 3H). ^13^C NMR (101 MHz; CD_3_OD): δ 155.4, 142.8, 142.2, 131.3, 129.8, 128.8, 127.7, 127.2, 126.7, 121.9, 112.8, 111.6, 57.4, 56.2, 45.6, 41.4, 41.1, 35.9, 33.0, 24.5, 22.6, 13.1. HRMS (ES+) Calcd. For C_22_H_27_NOBr, 400.1276 (M+H)^+^; found, 400.1277. [α]^20^_D_ –92.5° (*c* 1.0, CHCl_3_/MeOH (9/1)). Anal. Calcd. For C_22_H_27_BrClNO: C, 60.49; H, 6.23; N, 3.21. Found: C, 60.67; H, 6.33; N, 3.16.
*(1R,5R,9R)-6,11-Dimethyl-3-(2-bromophenethyl)-1,2,3,4,5,6-hexahydro-2,6-methanobenzo[d]azocin-8-ol* ((−)-**14**). General procedure was used, and the alkylation was achieved with 2-bromophenethyl bromide. The product was obtained as a pale-yellow solid (200 mg, 59%), mp (HCl salt) 233–235 °C (dec). ^1^H NMR (400 MHz; CD_3_OD): δ 7.51 (dd, *J* = 8.0, 1.0 Hz, 1H), 7.31–7.23 (m, 2H), 7.10–7.06 (m, 1H), 6.86 (d, *J* = 8.3 Hz, 1H), 6.67 (d, *J* = 2.5 Hz, 1H), 6.54 (dd, *J* = 8.2, 2.5 Hz, 1H), 3.05 (dd, *J* = 5.5, 3.1 Hz, 1H), 3.00–2.87 (m, 3H), 2.76 (dt, *J* = 11.8, 5.9 Hz, 1H), 2.71 (t, *J* = 4.7 Hz, 1H), 2.66–2.57 (m, 2H), 2.15 (td, *J* = 12.4, 3.2 Hz, 1H), 1.89 (qd, *J* = 7.0, 3.0 Hz, 1H), 1.81 (td, *J* = 12.9, 4.7 Hz, 1H), 1.32 (s, 3H), 1.30 (t, *J* = 2.4 Hz, 1H), 0.85 (d, *J* = 7.0 Hz, 3H). ^13^C NMR (101 MHz; CD_3_OD): δ 155.4, 142.1, 139.1, 132.4, 130.7, 127.85, 127.71, 127.5, 126.6, 123.8, 112.8, 111.6, 57.5, 54.7, 45.7, 41.3, 40.9, 35.9, 33.6, 24.5, 22.9, 13.1. HRMS (ES^+^) Calcd. For C_22_H_27_BrNO, 400.1276 (M+H)^+^; found, 400.1278. [α]^20^_D_ –88.2° (*c* 0.85, CHCl_3_/MeOH (9/1)). Anal. Calcd. For C_22_H_27_BrClNO • 0.1 H_2_O • 0.2 C_3_H_8_O: C, 60.24; H, 6.44; N, 3.11. Found: C, 60.32; H, 6.37; N, 3.02.
*(1R,5R,9R)-6,11-Dimethyl-3-(4-chlorophenethyl)-1,2,3,4,5,6-hexahydro-2,6-methanobenzo[d]azocin-8-ol* ((−)-**15**). General procedure was used, and the alkylation was achieved with 4-chlorophenethyl bromide. The product ((−)-**15**) was obtained as a pale-yellow solid (310 mg, 71%), mp (HCl salt) 300–302 °C (dec). ^1^H-NMR (400 MHz; CD_3_OD): δ 7.26–7.23 (m, 2H), 7.21–7.18 (m, 2H), 6.87 (d, *J* = 8.3 Hz, 1H), 6.67 (d, *J* = 2.5 Hz, 1H), 6.54 (dd, *J* = 8.2, 2.5 Hz, 1H), 3.01 (dd, *J* = 5.6, 3.1 Hz, 1H), 2.91 (d, *J* = 18.3 Hz, 1H), 2.80–2.72 (m, 3H), 2.68 (dd, *J* = 9.9, 4.1 Hz, 1H), 2.64–2.62 (m, 1H), 2.61–2.58 (m, 1H), 2.12 (td, *J* = 12.4, 3.2 Hz, 1H), 1.86 (dd, *J* = 7.0, 3.0 Hz, 1H), 1.80 (td, *J* = 12.9, 4.7 Hz, 1H), 1.32 (s, 3H), 1.30 (t, *J* = 2.5 Hz, 1H), 0.85 (d, *J* = 7.0 Hz, 3H). ^13^C NMR (101 MHz; CD_3_OD): δ 155.4, 142.2, 138.8, 131.5, 129.9, 128.0, 127.7, 126.6, 112.8, 111.6, 57.4, 56.3, 45.6, 41.4, 41.1, 35.9, 32.7, 24.5, 22.6, 13.1. HRMS (ES^+^) Calcd. For C_22_H_27_ClNO, 356.1781 (M+H)^+^; found, 356.1779. [α]^20^_D_ –105.0° (*c* 1.0, CHCl_3_/MeOH (9/1)). Anal. Calcd. For C_22_H_27_Cl_2_NO: C, 67.35; H, 6.94; N, 3.57. Found: C, 67.38; H, 6.69; N, 3.57.
*(1R,5R,9R)-6,11-Dimethyl-3-(3-chlorophenethyl)-1,2,3,4,5,6-hexahydro-2,6-methanobenzo[d]azocin-8-ol* ((−)-**16**). General procedure was used, and the alkylation was achieved with 3-chlorophenethyl bromide. The product was obtained as a pale-yellow solid (300 mg, 69%), mp (HCl salt) 258–260 °C (dec). ^1^H NMR (400 MHz; CD_3_OD): δ 7.26–7.22 (m, 2H), 7.18–7.13 (m, 2H), 6.88 (d, *J* = 8.3 Hz, 1H), 6.67 (d, *J* = 2.5 Hz, 1H), 6.54 (dd, *J* = 8.2, 2.6 Hz, 1H), 3.00 (dd, *J* = 5.6, 3.1 Hz, 1H), 2.90 (d, *J* = 18.3 Hz, 1H), 2.80–2.72 (m, 3H), 2.67 (dd, *J* = 5.5, 1.8 Hz, 1H), 2.65–2.57 (m, 2H), 2.12 (td, *J* = 12.4, 3.2 Hz, 1H), 1.85 (dt, *J* = 7.5, 4.0 Hz, 1H), 1.79 (dt, *J* = 12.9, 6.4 Hz, 1H), 1.32 (s, 3H), 1.30 (dd, *J* = 2.8, 2.2 Hz, 1H), 0.85 (d, *J* = 7.0 Hz, 3H). 13C NMR (101 MHz; CD_3_OD): δ 155.4, 142.5, 142.2, 133.7, 129.5, 128.3, 127.7, 126.74, 126.69, 125.8, 112.8, 111.6, 57.4, 56.2, 45.6, 41.5, 41.1, 35.9, 33.1, 24.5, 22.6, 13.1. HRMS (ES+) Calcd. For C_22_H_27_NOCl, 356.1781 (M+H)^+^; found, 356.1785. [α]^20^_D_ –104° (*c* 1.1, CHCl_3_/MeOH (9/1)). Anal. Calcd. For C_22_H_27_Cl_2_NO: C, 67.35; H, 6.94; N, 3.57. Found: C, 67.37; H, 6.72; N, 3.50.
*(1R,5R,9R)-6,11-Dimethyl-3-(2-chlorophenethyl)-1,2,3,4,5,6-hexahydro-2,6-methanobenzo[d]azocin-8-ol* ((−)-**17**). General procedure was used, and the alkylation was achieved with 2-chlorophenethyl bromide. The product was obtained as a pale-yellow solid (250 mg, 74%), Mp (HCl salt) 249–251 °C (dec). ^1^H NMR (400 MHz; CD_3_OD): δ 7.31 (ddd, *J* = 15.6, 7.6, 1.5 Hz, 2H), 7.23–7.14 (m, 2H), 6.86 (d, *J* = 8.3 Hz, 1H), 6.67 (d, *J* = 2.5 Hz, 1H), 6.54 (dd, *J* = 8.2, 2.5 Hz, 1H), 3.04 (dd, *J* = 5.4, 3.1 Hz, 1H), 3.00–2.86 (m, 3H), 2.75 (td, *J* = 11.6, 5.6 Hz, 1H), 2.72–2.58 (m, 3H), 2.14 (td, *J* = 12.4, 3.2 Hz, 1H), 1.88 (dd, *J* = 7.0, 3.0 Hz, 1H), 1.81 (td, *J* = 13.0, 4.7 Hz, 1H), 1.32 (s, 3H), 1.30 (s, 1H), 0.85 (d, *J* = 7.0 Hz, 3H). ^13^C NMR (101 MHz; CD_3_OD): δ 155.4, 142.2, 137.4, 133.5, 130.7, 129.1, 127.70, 127.61, 126.9, 126.6, 112.8, 111.6, 57.5, 54.6, 45.7, 41.3, 40.9, 35.9, 31.1, 24.5, 22.8, 13.1. HRMS (ES+) Calcd. For C_22_H_27_ClNO, 356.1781 (M+H)^+^; found, 356.1781. [α]^20^_D_ –90.6° (*c* 1.2, CHCl_3_/MeOH (9/1)). Anal. Calcd. For C_22_H_27_Cl_2_NO • 0.15 H_2_O • 0.1 C_3_H_8_O: C, 66.78; H, 7.06; N, 3.49. Found: C, 66.87; H, 6.97; N, 3.39.
*(1R,5R,9R)-6,11-Dimethyl-3-(4-methoxyphenethyl)-1,2,3,4,5,6-hexahydro-2,6-methanobenzo[d]azocin-8-ol* ((−)-**18**). General procedure was used, and the alkylation was achieved with 4-methoxyphenethyl bromide. The product was obtained as a pale-yellow solid (465 mg, 82%), mp (HCl salt) 164–166 °C. ^1^H-NMR (400 MHz; CD_3_OD): δ 7.12–7.09 (m, 2H), 6.87 (d, *J* = 8.3 Hz, 1H), 6.83–6.79 (m, 2H), 6.67 (d, *J* = 2.5 Hz, 1H), 6.54 (dd, *J* = 8.2, 2.5 Hz, 1H), 3.73 (s, 3H), 3.03 (dd, *J* = 5.6, 3.1 Hz, 1H), 2.91 (d, *J* = 18.3 Hz, 1H), 2.73–2.59 (m, 6H), 2.12 (td, *J* = 12.4, 3.2 Hz, 1H), 1.87 (dd, *J* = 7.0, 3.0 Hz, 1H), 1.80 (td, *J* = 12.9, 4.7 Hz, 1H), 1.33 (s, 3H), 1.30 (t, *J* = 2.4 Hz, 1H), 0.85 (d, *J* = 7.0 Hz, 3H). ^13^C NMR (101 MHz; CD_3_OD): δ 158.2, 155.4, 142.1, 131.8, 129.1, 127.7, 126.6, 113.5, 112.8, 111.6, 57.3, 56.8, 54.2, 45.7, 41.3, 41.0, 35.9, 32.4, 24.4, 22.5, 13.1. HRMS (ES^+^) Calcd. For C_23_H_30_NO_2_, 352.2277 (M+H)^+^; found, 352.2278. [α]^20^_D_ –109.0° (*c* 1.0, CHCl_3_/MeOH (9/1)). Anal. Calcd. For C_23_H_30_ClNO_2_ • 0.5 H_2_O: C, 69.59; H, 7.87; N, 3.53. Found: C, 69.88; H, 8.22; N, 3.23.
*(1R,5R,9R)-6,11-Dimethyl-3-(4-methylphenethyl)-1,2,3,4,5,6-hexahydro-2,6-methanobenzo[d]azocin-8-ol* ((−)-**19**). General procedure was used, and the alkylation was achieved with 4-methylphenethyl bromide. The product was obtained as a pale-yellow solid (460 mg, 72%), mp (HCl salt) 305–306 °C (dec). ^1^H-NMR (400 MHz; CD_3_OD): δ 7.09 (s, 4H), 6.89 (d, *J* = 8.3 Hz, 1H), 6.69 (d, *J* = 2.5 Hz, 1H), 6.56 (dd, *J* = 8.2, 2.6 Hz, 1H), 3.05 (dd, *J* = 5.7, 3.1 Hz, 1H), 2.93 (d, *J* = 18.3 Hz, 1H), 2.79–2.60 (m, 6H), 2.28 (s, 3H), 2.13 (td, *J* = 12.4, 3.3 Hz, 1H), 1.90 (td, *J* = 6.9, 2.9 Hz, 1H), 1.82 (td, *J* = 12.9, 4.7 Hz, 1H), 1.35 (s, 3H), 1.32 (t, *J* = 2.5 Hz, 1H), 0.87 (d, *J* = 7.0 Hz, 3H). ^13^C NMR (101 MHz; CD_3_OD): δ 156.9, 143.7, 138.3, 136.8, 130.1, 129.6, 129.2, 128.1, 114.3, 113.1, 58.7, 58.2, 47.2, 42.9, 42.5, 37.4, 34.4, 26.0, 24.0, 21.1, 14.6. HRMS (ES^+^) Calcd. For C_23_H_30_NO, 336.2327 (M+H)^+^; found, 336.2324. [α]^20^_D_ –110.8° (*c* 1.0, CHCl_3_/MeOH (9/1)). Anal. Calcd. For C_23_H_30_ClNO • 0.05 H_2_O: C, 74.09; H, 8.14; N, 3.76. Found: C, 74.10; H, 8.18; N, 3.74.
*(1R,5R,9R)-6,11-Dimethyl-3-(4-hydroxyphenethyl)-1,2,3,4,5,6-hexahydro-2,6-methanobenzo[d]azocin-8-ol* ((−)-**20**). General procedure was used, and the alkylation was achieved with 4-hydroxyphenethyl bromide. The product was obtained as a pale-yellow solid (170 mg, 52%), mp (HCl salt) 300–302 °C (dec). ^1^H NMR (400 MHz; CD_3_OD): δ 7.03–6.99 (m, 2H), 6.88 (d, *J* = 8.3 Hz, 1H), 6.70–6.66 (m, 3H), 6.54 (dd, *J* = 8.2, 2.5 Hz, 1H), 3.03 (dd, *J* = 5.5, 3.0 Hz, 1H), 2.96–2.83 (m, 2H), 2.73–2.59 (m, 6H), 2.11 (td, *J* = 12.4, 3.2 Hz, 1H), 1.87 (dt, *J* = 7.0, 3.5 Hz, 1H), 1.80 (td, *J* = 12.9, 4.7 Hz, 1H), 1.32 (s, 3H), 1.30 (d, *J* = 2.0 Hz, 1H), 0.85 (d, *J* = 7.0 Hz, 3H). ^13^C NMR (101 MHz; CD_3_OD): δ 155.40, 155.35, 142.1, 130.5, 129.1, 127.7, 126.6, 114.8, 112.8, 111.6, 57.2, 56.9, 45.7, 41.3, 40.9, 35.9, 32.4, 24.4, 22.4, 13.1. HRMS (ES+) Calcd. For C_22_H_28_NO_2_, 338.2120 (M+H)^+^; found, 338.2122. [α]^20^_D_ –120° (*c* 1.0, CHCl_3_/MeOH (9/1Anal. Calcd. For C_22_H_28_ClNO_2_ • 0.05 H_2_O • 0.2 C_3_H_8_O: C, 70.17; H, 7.74; N, 3.62. Found: C, 70.24; H, 7.83; N, 3.70.
*(1R,5R,9R)-6,11-Dimethyl-3-(2,4-dichlorophenethyl)-1,2,3,4,5,6-hexahydro-2,6-methanobenzo[d]azocin-8-ol* ((−)-**21**). General procedure was used, and the alkylation was achieved with 2,4-dichlorophenethyl bromide. The product was obtained as a pale-yellow solid (220 mg, 61%), Mp (HCl salt) 240–241 °C. ^1^H NMR (400 MHz; CD_3_OD): δ 7.39 (d, *J* = 2.1 Hz, 1H), 7.28 (d, *J* = 8.3 Hz, 1H), 7.23 (dd, *J* = 8.2, 2.1 Hz, 1H), 6.86 (d, *J* = 8.3 Hz, 1H), 6.67 (d, *J* = 2.5 Hz, 1H), 6.54 (dd, *J* = 8.2, 2.5 Hz, 1H), 3.01 (dt, *J* = 5.6, 2.8 Hz, 1H), 2.96–2.89 (m, 2H), 2.87–2.83 (m, 1H), 2.77–2.71 (m, 1H), 2.68 (t, *J* = 9.3 Hz, 1H), 2.63–2.56 (m, 2H), 2.13 (td, *J* = 12.4, 3.2 Hz, 1H), 1.87 (tt, *J* = 7.0, 3.5 Hz, 1H), 1.79 (td, *J* = 12.9, 4.7 Hz, 1H), 1.32 (s, 3H), 1.29–1.25 (m, 1H), 0.84 (d, *J* = 7.0 Hz, 3H). ^13^C NMR (101 MHz; CD_3_OD): δ 155.4, 142.2, 136.4, 134.3, 132.4, 131.8, 128.6, 127.7, 127.0, 126.6, 112.8, 111.6, 57.6, 54.3, 45.6, 41.4, 41.0, 35.9, 30.6, 24.5, 22.9, 13.1. HRMS (ES+) Calcd. For C_22_H_26_Cl_2_NO, (M+H)^+^ 390.1391; found, 390.1387. [α]^20^_D_ –95.6° (*c* 1.1, CHCl_3_/MeOH (9/1)). Anal. Calcd. For C_22_H_26_Cl_3_NO • 0.55 H_2_O: C, 61.56; H, 6.09; N, 3.12. Found: C, 61.59; H, 6.17; N, 3.03.
*(1R,5R,9R)-6,11-Dimethyl-3-(2,6-dichlorophenethyl)-1,2,3,4,5,6-hexahydro-2,6-methanobenzo[d]azocin-8-ol* ((−)-**22**). General procedure was used, and the alkylation was achieved with 2,6-dichlorophenethyl bromide. The product was obtained as a pale-yellow solid (245 mg, 66%), mp (HCl salt) 241–243 °C. ^1^H NMR (400 MHz; CD_3_OD): δ 7.34 (d, *J* = 8.0 Hz, 2H), 7.17 (t, *J* = 8.1 Hz, 1H), 6.86 (d, *J* = 8.3 Hz, 1H), 6.67 (d, *J* = 2.5 Hz, 1H), 6.54 (dd, *J* = 8.2, 2.5 Hz, 1H), 3.21–3.13 (m, 2H), 3.10 (t, *J* = 4.5 Hz, 1H), 2.93 (d, *J* = 18.4 Hz, 1H), 2.73 (dtd, *J* = 17.4, 11.5, 5.4 Hz, 3H), 2.59 (td, *J* = 11.9, 5.1 Hz, 1H), 2.19 (td, *J* = 12.4, 3.1 Hz, 1H), 1.93–1.87 (m, 1H), 1.83 (dt, *J* = 12.9, 6.5 Hz, 1H), 1.34–1.31 (m, 4H), 0.86 (d, *J* = 7.0 Hz, 3H). ^13^C NMR (101 MHz; CD_3_OD): δ 155.4, 142.1, 135.18, 135.07, 128.26, 128.08, 127.7, 126.4, 112.8, 111.6, 57.7, 52.2, 45.8, 41.2, 40.7, 35.8, 28.8, 24.4, 23.0, 13.1. HRMS (ES+) Calcd. For C_22_H_26_Cl_2_NO, (M+H)^+^ 390.1391; found, 390.1389. [α]^20^_D_ –89.1° (*c* 1.1, CHCl_3_/MeOH (9/1)). Anal. Calcd. For C_22_H_26_Cl_3_NO • 0.4 H_2_O • 0.2 C_3_H_8_O: C, 60.86; H, 6.42; N, 3.14. Found: C, 60.92; H, 6.35; N, 3.07.
*(1R,5R,9R)-6,11-Dimethyl-3-(phenethyl)-1,2,3,4,5,6-hexahydro-2,6-methanobenzo[d]azocin-8-ol* ((−)-**23**). General procedure was used, and the alkylation was achieved with phenethyl bromide. The product was obtained as a pale-yellow solid (244 mg, 64%), mp (HCl salt) 277–280 °C (dec). ^1^H NMR (400 MHz; CD_3_OD): δ 7.27–7.19 (m, 4H), 7.18–7.14 (m, 1H), 6.88 (d, *J* = 8.3 Hz, 1H), 6.67 (d, *J* = 2.5 Hz, 1H), 6.55 (dd, *J* = 8.2, 2.5 Hz, 1H), 3.07 (dd, *J* = 5.5, 3.1 Hz, 1H), 2.97–2.90 (m, 1H), 2.83–2.76 (m, 3H), 2.73–2.63 (m, 3H), 2.15 (td, *J* = 12.5, 3.2 Hz, 1H), 1.88 (ddd, *J* = 6.4, 5.8, 4.4 Hz, 1H), 1.81 (dt, *J* = 13.0, 6.5 Hz, 1H), 1.33 (s, 3H), 1.31 (d, *J* = 2.8 Hz, 1H), 0.86 (d, *J* = 7.0 Hz, 3H). ^13^C NMR (101 MHz; CD3OD): δ 155.4, 142.1, 139.8, 128.24, 128.05, 127.7, 126.5, 125.8, 112.8, 111.6, 57.4, 56.5, 45.7, 41.2, 40.9, 35.8, 33.3, 24.4, 22.5, 13.0. HRMS (ES+) Calcd. For C_22_H_28_NO, 322.2171 (M+H)^+^; found, 322.2170. [α]^20^_D_ –101.5° (*c* 1.1, CHCl_3_/MeOH (9/1)). Anal. Calcd. For C_22_H_28_ClNO • 0.05 H2O: C, 73.64; H, 7.89; N, 3.90. Found: C, 73.67; H, 7.79; N, 3.83.
*(1R,5R,9R)-6,11-Dimethyl-3-(phenylpropyl)-1,2,3,4,5,6-hexahydro-2,6-methanobenzo[d]azocin-8-ol* ((−)-**24**). General procedure was used, and the alkylation was achieved with phenylpropyl bromide. The product (−)-**24** was obtained as a pale-yellow solid (255 mg, 80%), mp (HCl salt) 209–211 °C. ^1^H NMR (400 MHz; CD_3_OD): δ 7.26–7.22 (m, 2H), 7.19–7.12 (m, 3H), 6.87 (d, *J* = 8.3 Hz, 1H), 6.67 (d, *J* = 2.5 Hz, 1H), 6.55 (dd, *J* = 8.3, 2.5 Hz, 1H), 3.06 (dd, *J* = 5.5, 3.1 Hz, 1H), 2.87 (d, *J* = 18.5 Hz, 1H), 2.72–2.56 (m, 6H), 2.15 (td, *J* = 12.6, 3.3 Hz, 1H), 1.90–1.75 (m, 4H), 1.34–1.31 (m, 4H), 0.84 (d, *J* = 7.0 Hz, 3H). ^13^C NMR (101 MHz; CD_3_OD): δ 155.6, 141.8, 141.5, 128.0, 127.8, 125.9, 125.6, 113.0, 111.6, 57.4, 53.7, 45.8, 40.8, 40.5, 35.7, 33.2, 28.1, 24.3, 22.5, 12.9. HRMS (ES+) Calcd. For C_23_H_30_NO, (M+H)^+^ 336.2327; found, 336.2329. [α]^20^_D_ –73.1° (*c* 1.1, CHCl_3_/MeOH (9/1)). Anal. Calcd. For C_23_H_30_ClNO • 0.2 H_2_O: C, 73.56; H, 8.16; N, 3.73. Found: C, 73.57; H, 8.02; N, 3.64.
*(1R,5R,9R)-6,11-Dimethyl-3-(cinnamyl)-1,2,3,4,5,6-hexahydro-2,6-methanobenzo[d]azocin-8-ol* ((−)-**25**). General procedure was used, and the alkylation was achieved with cinnamyl bromide. The product (−)-**25** was obtained as an off-white foam (200 mg, 66%), mp (HCl salt) 162–164 °C. ^1^H NMR (400 MHz; CD_3_OD): δ 7.39–7.37 (m, 2H), 7.29–7.25 (m, 2H), 7.21–7.17 (m, 1H), 6.91 (d, *J* = 8.3 Hz, 1H), 6.67 (d, *J* = 2.5 Hz, 1H), 6.60–6.55 (m, 2H), 6.26 (dt, *J* = 15.8, 7.0 Hz, 1H), 3.37 (ddd, *J* = 13.4, 7.1, 1.0 Hz, 1H), 3.01–2.95 (m, 2H), 2.65 (dd, *J* = 18.4, 6.0 Hz, 1H), 2.59–2.55 (m, 1H), 2.12 (td, *J* = 12.5, 3.2 Hz, 1H), 1.88 (dd, *J* = 7.0, 3.1 Hz, 1H), 1.79 (td, *J* = 12.9, 4.7 Hz, 1H), 1.32 (s, 3H), 1.30 (t, *J* = 2.5 Hz, 1H), 0.83 (d, *J* = 7.0 Hz, 3H). ^13^C NMR (101 MHz; CD_3_OD): δ 155.4, 142.2, 136.8, 133.4, 128.2, 127.7, 127.2, 126.6, 125.9, 125.4, 112.8, 111.6, 57.2, 56.8, 45.4, 41.3, 41.0, 35.9, 24.5, 22.5, 13.1. HRMS (ES+) Calcd. For C_23_H_28_NO, (M+H)^+^ 334.2171; found, 334.2171. [α]^20^_D_ –118.3° (*c* 1.1, CHCl_3_/MeOH (9/1)). Anal. Calcd. For C_23_H_28_ClNO • 0.5 H_2_O: C, 72.9; H, 7.71; N, 3.70. Found: C, 73.00; H, 7.52; N, 3.48.
*(1R,5R,9R)-3-(2-(1H-Indol-3-yl)ethyl)-6,11-dimethyl-1,2,3,4,5,6-hexahydro-2,6-methanobenzo[d]azocin-8-ol* ((−)-**26**). General procedure was used, and the alkylation was achieved with 3-(2-bromoethyl)-1H-indole. The product was obtained as a pale-yellow solid (250 mg, 80%), mp 115–118 °C. ^1^H NMR (400 MHz; CD_3_OD): δ 7.52 (d, *J* = 7.8 Hz, 1H), 7.30 (d, *J* = 8.1 Hz, 1H), 7.08–7.04 (m, 2H), 6.98 (td, *J* = 7.5, 0.8 Hz, 1H), 6.87 (d, *J* = 8.3 Hz, 1H), 6.68 (d, *J* = 2.5 Hz, 1H), 6.55 (dd, *J* = 8.2, 2.5 Hz, 1H), 3.13 (dd, *J* = 5.5, 3.0 Hz, 1H), 2.98–2.92 (m, 3H), 2.90–2.85 (m, 1H), 2.81 (dd, *J* = 10.7, 4.6 Hz, 1H), 2.71–2.65 (m, 2H), 2.19–2.13 (m, 1H), 1.92 (dd, *J* = 7.0, 3.0 Hz, 1H), 1.85 (dt, *J* = 13.0, 6.5 Hz, 1H), 1.35–1.32 (m, 4H), 0.87 (d, *J* = 7.0 Hz, 3H). ^13^C NMR (101 MHz; CD_3_OD): δ 155.4, 142.1, 136.7, 127.7, 127.2, 126.5, 121.6, 120.9, 118.1, 117.7, 112.8, 112.3, 111.6, 110.8, 57.2, 55.4, 45.8, 41.2, 40.9, 35.9, 24.4, 22.63, 22.50, 13.1. HRMS (ES+) Calcd. For C_24_H_29_N_2_O, (M+H)^+^ 361.2280; found, 361.2285. [α]^20^_D_ –78.5° (*c* 1.0, CHCl_3_/MeOH (9/1)). Anal. Calcd. For C_24_H_28_N_2_O • 0.7 H_2_O • 0.25 CHCl_3_: C, 72.28; H, 7.42; N, 6.95. Found: C, 72.37; H, 7.51; N, 6.80.
*1-((1R,5R,9R)-8-Hydroxy-6,11-dimethyl-1,4,5,6-tetrahydro-2,6-methanobenzo[d]azocin-3(2H)-yl)-2-(1-methylpiperidin-4-yl)ethan-1-one* (−)-**27**). To a stirred solution of 2-(1-methylpiperidin-4-yl)acetic acid (219 mg, 1.05 equiv, 1.39 mmol), N,N-diisopropylethylamine (685 mg, 923 µL, 4 equiv, 5.30 mmol) and (−)-**2**•bromocamphorsulfonate (700 mg, 1 equiv, 1.32 mmol) in DMF at 0 ^°^C, HATU (529 mg, 1.05 equiv, 1.39 mmol) was added. The reaction was stirred for 16 h. The reaction was diluted with water, and saturated NaHCO_3_ was added to it to adjust the pH to ~8.5. The aqueous solution was extracted with CHCl_3_/MeOH (9/1). The organic extractions were combined and washed with H_2_O (3 × 20 mL) to remove DMF. The organic extractions were then washed with brine (2 × 20 mL). The organic layer was dried over anhydrous Na_2_SO_4_, filtered, and the solvent was removed in vacuo. The crude residue was purified by flash chromatography (2–25% CMA in CHCl_3_) to give (−)-**27** as an off-white foam (400 mg, 97%). ^1^H NMR (400 MHz; CD_3_OD): δ 6.87 (d, *J* = 8.3 Hz, 1H), 6.66 (d, *J* = 2.5 Hz, 1H), 6.54 (dd, *J* = 8.2, 2.5 Hz, 1H), 2.96 (dd, *J* = 5.6, 3.0 Hz, 1H), 2.91–2.83 (m, 3H), 2.67–2.47 (m, 4H), 2.24 (s, 3H), 2.08 (td, *J* = 12.5, 3.2 Hz, 1H), 2.00 (t, *J* = 11.0 Hz, 2H), 1.84 (qd, *J* = 7.5, 3.2 Hz, 1H), 1.74 (ddd, *J* = 15.4, 11.3, 3.9 Hz, 3H), 1.45 (dd, *J* = 10.2, 5.7 Hz, 2H), 1.31–1.26 (m, 7H), 0.84 (d, *J* = 7.0 Hz, 3H). ^13^C NMR (101 MHz; CD_3_OD): δ 155.4, 142.1, 127.7, 126.5, 112.8, 111.6, 57.0, 55.2, 52.0, 45.7, 44.9, 41.3, 40.9, 35.9, 33.5, 33.1, 31.64, 31.56, 24.4, 22.3, 13.1. HRMS (ES+) Calcd. For C_22_H_33_N_2_O_2_, (M+H)^+^ 357.2542; found, 357.2539.
*(1R,5R,9R)-6,11-Dimethyl-3-(2-(1-methylpiperidin-4-yl)ethyl)-1,2,3,4,5,6-hexahydro-2,6-methanobenzo[d]azocin-8-ol* ((−)-**28**). Amide (−)-**27** (0.307 g, 1 equiv, 861 µmol) was dissolved in THF (7 mL). To this, a freshly prepared borane solution (2.58 mL, 4 molar, 12 equiv, 10.03 mmol) was added. The solution was heated at 60 °C for 24 h. The reaction was quenched with anhydrous MeOH at 0 °C and stirred for 30 min. The solvent was removed in vacuo and then redissolved in MeOH and acidified with 2N HCl. The mixture was refluxed for 4 h. The reaction was cooled to room temperature and then basified with NH_4_OH solution to pH 9. The aqueous solution was extracted with CHCl_3_: MeOH (9:1). The organic layers were washed with brine and dried over anhydrous Na_2_SO_4_, filtered, and solvent removed in vacuo to give (−)-**28** as a white solid (170 mg, 58%), mp (HCl salt) 309–310 °C (dec). ^1^H NMR (400 MHz; CD_3_OD): δ 6.87 (d, *J* = 8.3 Hz, 1H), 6.66 (d, *J* = 2.5 Hz, 1H), 6.54 (dd, *J* = 8.2, 2.5 Hz, 1H), 2.96 (dd, *J* = 5.6, 3.0 Hz, 1H), 2.91–2.83 (m, 3H), 2.67–2.47 (m, 4H), 2.24 (s, 3H), 2.08 (td, *J* = 12.5, 3.2 Hz, 1H), 2.00 (t, *J* = 11.0 Hz, 2H), 1.84 (qd, *J* = 7.5, 3.2 Hz, 1H), 1.74 (ddd, *J* = 15.4, 11.3, 3.9 Hz, 3H), 1.45 (dd, *J* = 10.2, 5.7 Hz, 2H), 1.31–1.26 (m, 7H), 0.84 (d, *J* = 7.0 Hz, 3H). ^13^C NMR (101 MHz; CD_3_OD): δ 155.4, 142.1, 127.7, 126.5, 112.8, 111.6, 57.0, 55.2, 52.0, 45.7, 44.9, 41.3, 40.9, 35.9, 33.5, 33.1, 31.64, 31.56, 24.4, 22.3, 13.1. HRMS (ES+) Calcd. For C_22_H_35_N_2_O, (M+H)^+^ 343.2749; found, 343.2746. [α]^20^_D_ –87.5° (*c* 1.1, CHCl_3_/MeOH (9/1)). Anal. Calcd. For C_22_H_36_Cl_2_N_2_O • 0.2 C_3_H_8_O • 1.15 H_2_O: C, 60.57; H, 8.97; N, 6.25. Found: C, 60.57; H, 8.99; N, 6.26.
*(1R,5R,9R)-6,11-Dimethyl-3-(3-(4-nitrophenyl)propyl)-1,2,3,4,5,6-hexahydro-2,6-methanobenzo[d]azocin-8-ol* ((−)-**31**). General procedure was used, and the alkylation was achieved with **30** as the alkylating agent (obtained from 3-(4-nitrophenyl)propan-1-ol, **29**). The product (−)-**31** was obtained as a pale-yellow solid (252 mg, 70%), mp (HCl salt) 265–266 °C (dec). ^1^H NMR (400 MHz; CD_3_OD): δ 8.15–8.11 (m, 2H), 7.46–7.42 (m, 2H), 6.85 (d, *J* = 8.3 Hz, 1H), 6.65 (d, *J* = 2.5 Hz, 1H), 6.53 (dd, *J* = 8.2, 2.6 Hz, 1H), 2.92 (dd, *J* = 5.6, 3.1 Hz, 1H), 2.85 (d, *J* = 18.3 Hz, 1H), 2.76 (t, *J* = 7.6 Hz, 2H), 2.65–2.45 (m, 4H), 2.06 (td, *J* = 12.4, 3.2 Hz, 1H), 1.90–1.80 (m, 3H), 1.76 (td, *J* = 12.9, 4.7 Hz, 1H), 1.30 (s, 3H), 1.27 (t, *J* = 2.5 Hz, 1H), 0.82 (d, *J* = 7.0 Hz, 3H). ^13^C NMR (101 MHz; CD_3_OD): δ 155.4, 150.2, 146.3, 142.2, 129.11, 129.06, 127.6, 126.6, 123.1, 112.7, 111.6, 57.2, 53.7, 45.6, 41.4, 41.0, 35.9, 33.1, 28.0, 24.5, 22.5, 13.1. HRMS (ES+) Calcd. For C_23_H_29_N_2_O_3_, (M+H)^+^ 381.2178; found, 381.2174. [α]^20^_D_ –77.5° (*c* 1.1, CHCl_3_/MeOH (9/1)). Anal. Calcd. For C_23_H_29_ClN_2_O_3_ **•** 0.05 H_2_O: C, 66.11; H, 7.02; N, 6.70. Found: C, 65.97; H, 6.75; N, 6.62.
*3-(4-Nitrophenyl)propyl methanesulfonate* (**30**). 3-(4-Nitrophenyl)propan-1-ol (510 mg, 1 equiv, 2.81 mmol) was dissolved in anhydrous dichloromethane (15 mL) and cooled to 0 °C. Triethylamine (427 mg, 588 µL, 1.5 equiv, 4.22 mmol) and methanesulfonyl chloride (355 mg, 241 µL, 1.1 equiv, 3.10 mmol) were added to it, and the reaction was stirred at 0 °C for 1 h. The reaction was diluted with water and extracted with dichloromethane (15 mL). The organic layer was washed with saturated NaHCO_3_ (15 mL) and brine (15 mL) and dried over anhydrous Na_2_SO_4_. The organic layer was filtered, and the solvent was removed in vacuo to give a pale-yellow oil. The crude product was purified by flash chromatography (0–80% EtOAc/hexanes) to give **30** as a pale-yellow oil that solidified on standing (700 mg, 96%). ^1^HNMR (400 MHz; CD_3_OD): δ 8.16–8.13 (m, 2H), 7.47–7.44 (m, 2H), 4.24 (t, *J* = 6.2 Hz, 2H), 3.05 (s, 3H), 2.86 (t, *J* = 7.7 Hz, 2H), 2.11–2.04 (m, 2H). ^13^C NMR (101 MHz; CD_3_OD): δ 148.9, 146.5, 129.2, 123.2, 69.1, 35.6, 31.0, 30.0. HRMS (ES+) Calcd. For C_10_H_13_NO_5_S, (M+Na)^+^ 282.0412; found, 282.0413.
*rac-8-Methoxy-6,11-dimethyl-1,2,3,4,5,6-hexahydro-2,6-methanobenzo[d]azocine* (**32**) [29]. 6,11-Dimethyl-1,2,3,4,5,6-hexahydro-2,6-methanobenzo[d]azocin-8-ol (*rac*-normetazocine, (*rac*-**1**), 10 g, 1 equiv, 46 mmol) and potassium carbonate (13 g, 2 equiv, 92 mmol) were added to H_2_O (0.15 L), and the mixture was cooled to 0 °C for 20 min. In a separate Erlenmeyer flask, di-tert-butyl dicarbonate (10 g, 11 mL, 1 equiv, 46 mmol) was dissolved in 1,4-dioxane (75 mL). This solution was added to the aqueous mixture, and the reaction was warmed at 23 °C over the course of 18 h. The organic products were extracted with CHCl_3_ 3x, and the organic solution was washed with 1M HCl and brine. The combined organic phase was dried over sodium sulfate and concentrated in vacuo to provide tert-butyl 8-hydroxy-6,11-dimethyl-1,4,5,6-tetrahydro-2,6-methanobenzo[d]azocine-3(2H)-carboxylate (15 g, 47 mmol, 100%) as a yellow/orange oil. The tert-butyl compound, as a crude oil (15 g, 1 equiv, 46 mmol), was dissolved in 2N NaOH (94 mL) and cooled to 0 °C for 20 min. Dimethyl sulfate (5.8 g, 4.4 mL, 1 equiv, 46 mmol) was added, and the mixture kept at 23 °C for 4 h. Diethyl ether was added to dilute the reaction, and the aqueous phase extracted 3x with diethyl ether. The combined organic phase was washed with brine and concentrated in vacuo to a white foam. The foam was dissolved in 1M HCl (0.11 L) and methanol (0.11 L) and kept at 50 °C until TLC showed full conversion. Volatiles and H_2_O were removed in vacuo to provide *rac*-normetazocine methyl ether **32** (7.65 g, 33.1 mmol, 72%) as a foam. ^1^H-NMR (400 MHz; CDCl_3_): δ 7.06 (d, *J* = 8.4 Hz, 1H), 6.80 (d, *J* = 2.6 Hz, 1H), 6.76 (dd, *J* = 8.4, 2.6 Hz, 1H), 3.79 (s, 3H), 3.23–3.05 (m, 3H), 2.74 (td, *J* = 13.3, 3.6 Hz, 1H), 2.15 (dd, *J* = 7.0, 3.1 Hz, 1H), 1.99 (dd, *J* = 13.7, 4.7 Hz, 1H), 1.51 (dd, *J* = 14.0, 2.0 Hz, 1H), 1.42 (s, 3H), 0.88 (d, *J* = 7.0 Hz, 3H). ^13^C NMR (101 MHz; cdcl3): δ 158.8, 140.4, 128.7, 125.0, 111.9, 111.5, 55.2, 52.2, 38.6, 37.71, 37.54, 35.5, 27.1, 24.8, 13.1. The NMR spectra corresponded to those in the literature [29].
*rac-8-Methoxy-6,11-dimethyl-3,4,5,6-tetrahydro-2,6-methanobenzo[d]azocin-1(2H)-one* (**33**). In an argon-charged flame-dried flask, CrO_3_ (7.5 g, 1.5 equiv, 74.6 mmol) was dissolved in 10:1 H_2_O: H_2_SO_4_, 330 mL. To this, the methyl ether **32** (13 g, 1 equiv, 57.4 mmol) in 330 mL of 10:1 H_2_O: H_2_SO_4,_ was added rapidly, and the mixture was refluxed for 5 h. The mixture was cooled and basified with NH_4_OH to pH 9. The chromium precipitate (grey solid) was filtered off. The filtrate was extracted 2x with EtOAc. The organic phase was washed with brine, dried, and concentrated in vacuo. The residue was purified via flash chromatography with CMA (50:45:5) to provide 8.9 g (63% yield) of the ketone **33** as a yellow syrup. ^1^H-NMR (400 MHz; CDCl_3_): δ 8.05 (d, *J* = 8.7 Hz, 1H), 6.87 (dd, *J* = 8.7, 2.5 Hz, 1H), 6.81 (d, *J* = 2.5 Hz, 1H), 3.89 (d, *J* = 6.1 Hz, 3H), 3.28 (d, *J* = 2.8 Hz, 1H), 2.75 (ddd, *J* = 12.0, 5.3, 1.8 Hz, 1H), 2.60 (td, *J* = 12.4, 3.3 Hz, 1H), 2.18–2.08 (m, 2H), 1.92 (td, *J* = 12.9, 5.3 Hz, 1H), 1.49 (ddd, *J* = 13.0, 3.2, 1.9 Hz, 1H), 1.44 (s, 3H), 0.87 (d, *J* = 7.1 Hz, 3H). ^13^C NMR (101 MHz; CDCl_3_): δ 198.8, 164.8, 149.4, 128.1, 127.6, 112.0, 111.6, 63.2, 55.3, 43.6, 40.8, 39.2, 37.8, 25.9, 14.2, 13.7. HRMS: [C_15_H_19_NO_2_]H^+^ Calcd: 246.1494; Found: 246.1490. NMR spectra corresponded to those in the literature [29].
*rac-8-Methoxy-6,11-dimethyl-3-phenethyl-3,4,5,6-tetrahydro-2,6-methanobenzo[d]azocin-1(2H)-one* (*rac-***34**). In a flame-dried flask, the C8-ketone **33** (8.9 g, 1 equiv, 36 mmol), (2-bromoethyl) benzene (20 g, 15 mL, 3 equiv, 0.11 mol), and potassium carbonate (20 g, 4 equiv, 0.15 mol) were added to acetonitrile (120 mL). The mixture was heated at 80 °C for 18 h. The reaction mixture was filtered through celite to remove the remaining potassium carbonate. The filtrate was concentrated in vacuo, and the residual material was purified by flash chromatography using 30% ethyl acetate in hexanes. The *N*-phenethyl compound *rac*-**34** (10.2 g, 29.2 mmol, 80%) was isolated as a light brown oil. The oil was concentrated overnight in vacuo to give a light-yellow solid. ^1^H-NMR (400 MHz; CDCl_3_): δ 7.99 (d, *J* = 8.7 Hz, 1H), 7.25–7.20 (m, 4H), 7.15 (s, 1H), 6.82 (dd, *J* = 8.7, 2.5 Hz, 1H), 6.77 (d, *J* = 2.5 Hz, 1H), 3.84 (d, *J* = 5.0 Hz, 3H), 3.23 (d, *J* = 2.8 Hz, 1H), 3.01–2.94 (m, 1H), 2.80–2.73 (m, 3H), 2.56–2.49 (m, 1H), 2.10 (d, *J* = 10.0 Hz, 3H), 1.40 (s, 3H), 0.87 (d, *J* = 7.1 Hz, 3H). ^13^C NMR (101 MHz; CDCl_3_): δ 194.9, 164.7, 149.1, 140.4, 128.8, 128.2, 127.95, 127.83, 125.9, 111.9, 67.4, 57.1, 55.4, 46.3, 44.1, 41.6, 37.5, 34.0, 25.6, 13.7. NMR spectra agreed with those in the literature [29].

Optimized optical resolution of *rac*-8-methoxy-6,11-dimethyl-3-phenethyl-3,4,5,6-tetrahydro-2,6-methanobenzo[*d*]azocin-1(2*H*)-one (*rac*-**34**) to obtain 2*S*,6*R*,11*R*-(−)-**34** and 2*R*,6*S*,11*S*-(+)-**34** (1*S*,5*R*,9*R*-(−)-**34** and 1*R*,5*S*,9*S*-(+)-**34** in benzomorphan nomenclature). The *N*-phenethyl compound *rac*-**34** (3.14 g, 2 equiv, 8.99 mmol) as an orange oil was dissolved in acetone (4 mL). In a separate Erlenmeyer flask, L-tartaric acid (1.35 g, 2 equiv, 8.99 mmol) was dissolved in *N*,*N*-dimethylformamide (3 mL) with stirring and heating to 80 °C, and the acetone solution of *rac*-**34** was added. The combined solution was allowed to cool to 0 °C, and crystallization occurred after scratching the solution with a glass rod, providing a white crystalline tartrate salt of (−)-**34** (1.33 g), mp. 197–199 °C; [α]_25_^D^ = −48.6° (*c* 0.44, MeOH). The salt was collected via filtration and free-based with concentrated NH_4_OH. The free base was extracted with CHCl_3,_ and the aqueous phase was extracted 2x with CHCl_3_. The combined organic phases were washed 2x with saturated Na_2_CO_3_. After drying over Na_2_SO_4_ and solvent removal in vacuo, (−)-**34** (1.05 g, 3.00 mmol, 67%) was obtained as a yellow oil (see Figure 1 for IUPAC numbering of benzomorphans), [α]_25_^D^ –41.3° (*c* 1.08, MeOH). ^1^HNMR (400 MHz; C_6_H_6_): δ 8.34 (d, *J* = 8.7 Hz, 1H), 7.21 (dd, *J* = 16.8, 7.4 Hz, 15H), 7.08 (t, *J* = 7.1 Hz, 1H), 6.74 (s, 1H), 6.50 (dd, *J* = 8.6, 1.5 Hz, 1H), 3.34 (s, 1H), 3.21 (s, 3H), 3.16–3.01 (m, 2H), 2.84–2.77 (m, 1H), 2.65 (ddd, *J* = 12.2, 9.1, 5.5 Hz, 1H), 2.53–2.49 (m, 1H), 2.17–2.10 (m, 1H), 1.94–1.90 (m, 1H), 1.79–1.71 (m, 1H), 1.24–1.21 (m, 1H), 1.08 (s, 3H), 0.74 (d, *J* = 7.1 Hz, 3H). HRMS: [C_23_H_27_NO_2_]H^+^ Calculated: 350.2120; Found: 350.2123. The mother liquor after isolation of (−)-**34** was free-based with ammonia water and extracted 3x with CHCl_3_. After drying and removal of solvent in vacuo, 2.1 g of *rac*-**34** base was collected and dissolved in 3 mL of acetone. D-tartaric acid (0.9 g) was dissolved in 2 mL of DMF and added to the acetone solution. Crystallization was achieved using the same procedure as with (−)-**34** to give the D-tartrate salt of (+)-**34** as a solid, mp 197–199 °C, [α]_25_^D^ = +46.9° (*c* 0.48, MeOH). After conversion to the free base, (+)-**34** was isolated as a yellow oil, [α]_25_^D^ +44.4° (*c* 1.65, MeOH). ^1^H-NMR (400 MHz; C_6_H_6_): δ 8.27 (d, *J* = 8.6 Hz, 1H), 7.18–7.10 (m, 8H), 7.04–7.01 (m, 1H), 6.68 (s, 1H), 6.45 (dd, *J* = 8.7, 1.2 Hz, 1H), 6.11 (s, 1H), 3.28 (d, *J* = 2.1 Hz, 1H), 3.15 (s, 3H), 3.10–2.95 (m, 2H), 2.74 (ddd, *J* = 13.3, 8.5, 5.3 Hz, 1H), 2.59 (ddd, *J* = 12.1, 9.0, 5.6 Hz, 1H), 2.45 (dd, *J* = 11.9, 4.9 Hz, 1H), 2.08 (td, *J* = 12.4, 3.0 Hz, 1H), 1.89–1.84 (m, 1H), 1.69 (td, *J* = 12.8, 5.1 Hz, 1H), 1.17 (d, *J* = 12.7 Hz, 1H), 1.02 (s, 3H), 0.68 (d, *J* = 7.1 Hz, 3H). ^13^C NMR (101 MHz; C_6_D_6_): δ 193.6, 164.5, 148.6, 140.7, 128.8, 128.5, 128.2, 127.8, 127.51, 127.39, 125.8, 112.2, 111.1, 67.3, 56.9, 54.4, 46.2, 43.9, 41.6, 37.2, 34.0, 25.1, 13.3. HRMS: [C_23_H_27_NO_2_]H^+^ Calcd: 350.2120; Found: 350.2118. The absolute configuration of (+)-**34** was established as 2*R*,6*S*,11*S* by X-ray crystallographic analysis of the HBr salt (Figure 3). Anal. Calcd. For C_23_H_28_BrNO_2_ • 0.8 H_2_O • 0.25 CH_4_O: C 61.67; H 6.81; N 3.09. Found C 61.71; H 6.69; N 2.97.

*(2R,6S,11S)-8-Methoxy-6,11-dimethyl-3-phenethyl-1,2,3,4,5,6-hexahydro-2,6-methanobenzo[d]azocin-1-ol* ((2*R*,6*S*,11*S*)-(+)-**35**). In a flame-dried flask, (+)-**34** (0.580 g, 1 equiv, 1.66 mmol) was dissolved in THF (10 mL) and cooled to 0 °C. Lithium aluminum hydride (75.6 mg, 1.2 equiv, 1.99 mmol) was added, and the reaction was allowed to warm slowly to room temperature. The reaction was quenched successively with iPrOH, MeOH and water, followed by dilution with CHCl_3_. The heterogeneous mixture was diluted further with brine and extracted 3x with CHCl_3_. The combined organic phases were washed with ammonium chloride, dried, and the concentrated residue was purified by flash chromatography. The alcohol (+)-**35** (0.508 g, 1.45 mmol, 87.1%) was isolated as a colorless oil, and only a single diastereomer was observed in the NMR. The optical rotation was essentially the same as that determined for (−)-**35**, except for the sign of rotation, as expected for an enantiomeric pair. [α]_25_^D^ = +52.3° (c, 2.8, MeOH). ^1^H-NMR (400 MHz; CDCl_3_): δ 7.47 (d, *J* = 8.5 Hz, 1H), 7.29 (t, *J* = 7.3 Hz, 2H), 7.22–7.18 (m, 3H), 6.80 (dd, *J* = 8.5, 2.6 Hz, 1H), 6.69 (d, *J* = 2.6 Hz, 1H), 4.62 (d, *J* = 6.5 Hz, 1H), 3.14–3.08 (m, 1H), 2.93–2.86 (m, 2H), 2.78 (t, *J* = 7.2 Hz, 2H), 2.49 (dd, *J* = 9.9, 2.5 Hz, 2H), 2.08–2.02 (m, 1H), 1.83–1.76 (m, 1H), 1.32 (s, 3H), 1.01 (dt, *J* = 13.0, 2.4 Hz, 1H), 0.82 (d, *J* = 7.1 Hz, 3H). ^13^C-NMR (101 MHz, CDCl_3_): δ 158.86, 141.26, 140.04, 132.89, 128.70, 128.38, 126.09, 111.22, 110.89, 63.80, 62.96, 55.98, 55.11, 43.61, 37.61, 37.54, 37.19, 35.95, 25.98, 13.03. HRMS [C_23_H_29_NO_2_]H^+^ Calcd: 352.2277; Found: 352.2277.
*(2S,6R,11R)-8-Methoxy-6,11-dimethyl-3-phenethyl-1,2,3,4,5,6-hexahydro-2,6-methanobenzo[d]azocin-1-ol* (2*S*,6*R*,11*R*-(−)-**35**). In a flame-dried flask, (−)-**34** (0.550 g, 1 equiv, 1.57 mmol) was dissolved in THF (10 mL) and cooled to 0 °C. Lithium aluminum hydride (71.7 mg, 1.2 equiv, 1.89 mmol) was added, and the reaction was allowed to warm slowly to room temperature. The reaction was quenched successively with iPrOH, MeOH and H_2_O, followed by addition of CHCl_3_. Brine was added to the heterogeneous mixture, and it was extracted 3x with CHCl_3_. The combined organic phases were washed with NH_4_OH, dried, and purified by flash chromatography. The alcohol (−)-**35** (0.398 g, 1.13 mmol, 71.9%) was isolated as a colorless oil, [α]_25_^D^ –51.7° (c, 2.08, MeOH). ^1^H-NMR (400 MHz; CDCl_3_): δ 7.47 (d, *J* = 8.5 Hz, 1H), 7.29 (t, *J* = 7.3 Hz, 2H), 7.22–7.18 (m, 3H), 6.80 (dd, *J* = 8.5, 2.6 Hz, 1H), 6.69 (d, *J* = 2.6 Hz, 1H), 4.62 (d, *J* = 6.5 Hz, 1H), 3.11 (dt, *J* = 13.0, 6.6 Hz, 1H), 2.93–2.86 (m, 2H), 2.78 (t, *J* = 7.2 Hz, 2H), 2.49 (dd, *J* = 9.7, 2.4 Hz, 2H), 2.08–2.02 (m, 1H), 1.83–1.76 (m, 1H), 1.32 (s, 3H), 1.01 (dt, *J* = 13.0, 2.4 Hz, 1H), 0.82 (d, *J* = 7.1 Hz, 3H). ^13^C-NMR (101 MHz, CDCl_3_): δ 158.87, 141.25, 140.04, 132.89, 128.69, 128.38, 126.08, 111.23, 110.89, 63.81, 62.96, 55.98, 55.11, 43.62, 37.61, 37.54, 37.20, 35.96, 25.98, 13.03. HRMS: [C_23_H_29_NO_2_]H^+^ Calcd: 352.2277; Found: 352.2279.
*(2R,6S,11S)-6,11-Dimethyl-3-phenethyl-1,2,3,4,5,6-hexahydro-2,6-methanobenzo[d]azocine-1,8-diol* (2*R*,6*S*,11*S*-(+)-**36**). In a flame-dried flask, the aromatic ether (+)-**35** (0.493 g, 1 equiv, 1.40 mmol) was dissolved in dichloromethane (10 mL) and cooled to –78 °C. To this solution, boron tribromide (703 mg, 265 µL, 2 equiv, 2.81 mmol) was added, and the reaction was allowed to warm to room temperature. The reaction was cooled to 0 °C and quenched with MeOH followed by 2M HCl. The reaction was allowed to stir for 1 h and then basified with a saturated sodium bicarbonate solution. The aqueous phase was separated and extracted 3x with CHCl_3,_ followed by washing the combined organic phases with a saturated sodium bicarbonate solution. After drying, the organic phase was concentrated in vacuo, and the residue purified via flash chromatography, eluting with 7% CMA in CHCl_3_, to give **t**he phenol (+)-**36** (0.312 g, 925 µmol, 65.9%) as a white foam, [α]_25_^D^ +58.1° (c 1.14, MeOH). ^1^H-NMR (400 MHz; CDCl_3_): δ 7.40 (d, *J* = 8.3 Hz, 1H), 7.31–7.18 (m, 8H), 6.72–6.69 (m, 2H), 6.63 (d, *J* = 2.6 Hz, 1H), 4.62 (d, *J* = 6.5 Hz, 1H), 3.54 (s, 1H), 3.11 (td, *J* = 10.8, 5.3 Hz, 2H), 2.93–2.86 (m, 3H), 2.78 (q, *J* = 6.5 Hz, 2H), 2.51 (dd, *J* = 8.9, 2.2 Hz, 2H), 2.04 (dq, *J* = 10.6, 3.5 Hz, 2H), 1.83–1.75 (m, 2H), 1.34 (s, 1H), 1.31 (d, *J* = 12.9 Hz, 4H), 1.01 (dt, *J* = 13.0, 2.3 Hz, 1H), 0.87 (d, *J* = 7.3 Hz, 1H), 0.82 (d, *J* = 7.1 Hz, 3H). ^13^C-NMR (101 MHz, CDCl_3_): δ 154.99, 141.50, 139.99, 132.58, 130.07, 128.75, 128.68, 128.62, 128.40, 128.36, 126.11, 113.64, 111.53, 63.81, 62.91, 55.97, 43.60, 37.52, 37.49, 37.14, 35.93, 25.95, 13.00. HRMS: [C_22_H_28_NO_2_]^+^ Calcd: 338.2120; Found: 338.2116. The HCl salt of the phenol (+)-**36** was formed by dissolving the free base in a minimal amount of acetone, followed by the addition of 6 drops of concentrated HCl. The solution was concentrated to a thick wax that was triturated in diethyl ether at room temperature overnight to provide the HCl salt as a free-flowing off-white solid. Anal. Calcd. For C_22_H_28_ClNO_2_ • 0.7 H_2_O: C, 68.36; H, 7.67; N, 3.62. Found C, 68.53; H, 7.81; N, 3.45.
*(2S,6R,11R)-6,11-Dimethyl-3-phenethyl-1,2,3,4,5,6-hexahydro-2,6-methanobenzo[d]azocine-1,8-diol* (2*S*,6*R*,11*R)-*(−)-**36.** In a flame-dried flask, the aromatic ether ((−)-**35** (0.396 g, 1 equiv, 1.13 mmol) was dissolved in dichloromethane (10 mL) and cooled to –78 °C. To this solution, boron tribromide (0.564 mg, 213 µL, 2 equiv, 2.25 mmol) was added, and the reaction was allowed to warm to room temperature. The reaction was cooled to 0 °C and quenched with MeOH followed by 2M HCl. The reaction was allowed to stir for 1 h and then basified with a saturated sodium bicarbonate solution. The aqueous phase was extracted 3x with CHCl_3_, and the combined organic phases were washed with a saturated sodium bicarbonate solution. After drying, the organic phase was purified by flash chromatography, eluting with 7% CMA in CHCl_3_, to give (−)-**36** as a white foam (0.190 g, 563 µmol, 50.0%). [α]_25_^D^ –57.4° (*c* 1.45, MeOH) ^1^H-NMR (400 MHz; CDCl_3_): δ 7.40 (d, *J* = 8.3 Hz, 1H), 7.31–7.16 (m, 8H), 6.70 (td, *J* = 6.0, 2.8 Hz, 2H), 6.63 (d, *J* = 2.5 Hz, 1H), 4.62 (d, *J* = 6.5 Hz, 1H), 4.17 (s, 1H), 3.54 (s, 1H), 3.11 (dt, *J* = 13.0, 6.6 Hz, 2H), 2.89 (dq, *J* = 11.4, 7.2 Hz, 3H), 2.82–2.74 (m, 3H), 2.50 (t, *J* = 4.5 Hz, 2H), 2.04 (dtd, *J* = 14.2, 7.2, 3.5 Hz, 2H), 1.86–1.75 (m, 2H), 1.36–1.33 (m, 2H), 1.30 (s, 3H), 1.01 (dt, *J* = 12.9, 2.3 Hz, 1H), 0.88 (d, *J* = 7.2 Hz, 1H), 0.82 (d, *J* = 7.1 Hz, 3H). ^13^C NMR (101 MHz, CDCl_3_): δ 155.12, 141.48, 139.97, 132.39, 130.07, 128.75, 128.68, 128.61, 128.40, 128.37, 126.12, 126.09, 113.69, 111.73, 111.56, 73.50, 63.83, 62.90, 59.67, 57.78, 55.96, 43.60, 37.49, 37.13, 35.92, 25.96, 14.38, 13.00.
*(2S,6R,11R)-8-Methoxy-1,6,11-trimethyl-3-phenethyl-1,2,3,4,5,6-hexahydro-2,6-methanobenzo[d]azocin-1-ol* (2*S*,6*R*,11*R*-(−)-**37**). In an argon-charged, flame-dried flask, ((−)-**34** (0.5 g, 1 equiv, 1 mmol) was dissolved in THF (15 mL) and cooled to –78 °C. Methyllithium (0.09 g, 3 mL, 1.3 molar, 3 equiv, 4 mmol) was added, and the reaction was warmed to room temperature overnight. The reaction mixture was quenched with a concentrated NH_4_Cl solution, and it was extracted with CHCl_3_. The aqueous phase was separated and extracted with CHCl_3_ 2x_,_ and the combined organic phases were washed with brine. The organic phase was then dried with sodium sulfate, filtered, and purified via flash chromatography.to give (−)-**37** as a dark amber oil (0.454 g, 1.24 mmol, 90%). ^1^H NMR (400 MHz; CDCl_3_): δ 7.52 (d, *J* = 8.6 Hz, 1H), 7.32–7.25 (m, 3H), 7.24–7.19 (m, 3H), 6.83 (dt, *J* = 8.6, 3.2 Hz, 1H), 6.69 (d, *J* = 2.7 Hz, 1H), 3.80 (d, *J* = 6.4 Hz, 3H), 3.14–3.07 (m, 1H), 2.98–2.89 (m, 2H), 2.82–2.73 (m, 3H), 2.66–2.42 (m, 4H), 2.15 (qd, *J* = 7.2, 3.4 Hz, 1H), 1.82 (td, *J* = 13.2, 4.8 Hz, 1H), 1.54 (d, *J* = 7.7 Hz, 3H), 1.34 (d, *J* = 10.3 Hz, 3H), 0.95 (t, *J* = 8.6 Hz, 3H)**.** ^13^C-NMR (101 MHz, CDCl_3_): δ 128.85, 128.70, 128.42, 128.15, 127.79, 126.15, 111.64, 110.83, 69.26, 67.29, 60.31, 55.76, 55.10, 55.08, 43.38, 43.34, 37.97, 37.95, 36.99, 35.99, 33.62, 26.32, 23.69, 16.35. HRMS: [C_24_H_32_NO_2_]^+^ Calcd: 366.2433; found: 366.2437.
*(2R,6S,11S)-8-Methoxy-1,6,11-trimethyl-3-phenethyl-1,2,3,4,5,6-hexahydro-2,6-methanobenzo[d]azocin-1-ol* (2*R*,6*S*,11*S*-(+)-**37**). In an argon-charged flame-dried flask, (+)-**34** (0.5 g, 1 equiv, 1 mmol) was dissolved in THF (15 mL) and cooled to –78 °C. Methyllithium (0.09 g, 3 mL, 1.3 molar, 3 equiv, 4 mmol) was added, and the reaction was allowed to warm to room temperature. The reaction mixture was quenched with a concentrated aqueous NH_4_Cl solution and extracted with CHCl_3_. The aqueous phase was separated and extracted 2x with CHCl_3_, and the combined organic phases were washed with brine. The organic phase was then dried with sodium sulfate, filtered, and purified by flash chromatography to give (+)-**37** as a dark amber oil (0.51 g, 1.4 mmol, 100%). ^1^H NMR (400 MHz; CDCl_3_): δ 7.52 (d, *J* = 8.6 Hz, 1H), 7.31–7.23 (m, 3H), 7.23–7.19 (m, 3H), 6.84 (dt, *J* = 8.6, 2.6 Hz, 1H), 6.69 (d, *J* = 2.6 Hz, 1H), 3.79 (d, *J* = 6.5 Hz, 3H), 3.13–3.06 (m, 1H), 2.98–2.88 (m, 2H), 2.84–2.77 (m, 2H), 2.65 (d, *J* = 1.8 Hz, 1H), 2.59–2.45 (m, 2H), 2.15 (qd, *J* = 7.0, 3.4 Hz, 1H), 1.81 (td, *J* = 13.2, 4.8 Hz, 1H), 1.53 (s, 3H), 1.33 (s, 3H), 1.07–1.04 (m, 1H), 0.95 (t, *J* = 7.1 Hz, 3H). ^13^C NMR (101 MHz, CDCl_3_): δ 158.67, 140.41, 128.82, 128.69, 128.40, 128.21, 127.77, 126.13, 111.88, 111.64, 110.82, 69.25, 67.27, 55.80, 55.10, 43.42, 38.09, 38.07, 37.99, 37.02, 36.03, 33.62, 26.33, 16.36. HRMS: [C_24_H_32_NO_2_]^+^ Calcd: 366.2433; found: 366.2433.
*(2R,6S,11S)-6,11-Dimethyl-1-methylene-3-phenethyl-1,2,3,4,5,6-hexahydro-2,6-methanobenzo[d]azocin-8-ol* (2*R*,6*S*,11*S*-(+)-**38**). In a flame-dried flask, (+)-**37** (0.5 g, 1 equiv, 1 mmol) was dissolved in dichloromethane (7 mL) and cooled to –78 °C. To this solution, boron tribromide (498 mg, 0.188 mL, 1 equiv, 1.99 mmol) was added, and the reaction was allowed to warm to room temperature. The reaction was quenched with MeOH followed by an equal volume of 2M HCl. The solution was heated to 75 °C for 2 h, followed by addition of a sodium bicarbonate to a pH of 7.5–8. The aqueous phase was extracted with CHCl_3_ 3x. The combined organic phases were washed with a saturated sodium bicarbonate solution and brine, then dried with sodium sulfate. The organic phase was purified by flash chromatography, eluting with 15% CMA in CHCl_3_ to give (+)-**38**. ^1^H NMR (400 MHz; CD_3_OD): δ 7.53 (d, *J* = 8.6 Hz, 1H), 7.23–7.20 (m, 2H), 7.13 (td, *J* = 6.8, 2.9 Hz, 3H), 6.67 (d, *J* = 2.6 Hz, 1H), 6.60 (dd, *J* = 8.6, 2.6 Hz, 1H), 5.68 (s, 1H), 4.80 (s, 1H), 3.33 (s, 1H), 3.30 (t, *J* = 3.5 Hz, 1H), 2.83 (dq, *J* = 6.6, 4.7 Hz, 1H), 2.76–2.68 (m, 2H), 2.61–2.57 (m, 1H), 2.46–2.41 (m, 1H), 1.97–1.90 (m, 2H), 1.84 (dt, *J* = 12.7, 6.3 Hz, 1H), 1.34–1.31 (m, 3H), 0.78 (d, *J* = 7.0 Hz, 3H). ^13^C NMR (101 MHz, CD_3_OD): δ 157.92, 142.09, 140.03, 136.67, 128.27, 128.02, 126.59, 125.70, 123.34, 113.34, 111.82, 110.61, 65.90, 56.79, 45.86, 41.80, 41.78, 36.94, 33.12, 24.66, 13.24. HRMS: [C_23_H_28_NO]^+^ Calculated: 334.2171; Found: 334.2165. The HCl salt of (+)-**38** was obtained by dissolving the free base in a minimal amount of acetone. The solution was concentrated to a pale syrup in vacuo, followed by addition of diethyl ether. The mixture was stirred overnight to give (+)-**38**•HCl as a pale beige solid. Anal. Calcd. For C_23_H_28_ClNO • 0.7 H_2_O C, 72.21; H, 7.75; N, 3.66. Found C, 72.13; H, 7.55; N, 3.55.
*(2S,6R,11R)-6,11-Dimethyl-1-methylene-3-phenethyl-1,2,3,4,5,6-hexahydro-2,6-methanobenzo[d]azocin-8-ol* (2*S*,6*R*,11*R*-(−)-**38**). In a flame-dried flask, (−)-**37** (0.454 g, 1 equiv, 1.24 mmol) was dissolved in dichloromethane (7 mL) and cooled to –78 °C. To this solution, boron tribromide (615 mg, 0.232 mL, 1.98 equiv, 2.45 mmol) was added, and the reaction was allowed to warm to room temperature. The reaction was quenched with MeOH followed by an equal volume of 2M HCl. The solution was heated to 75 °C for 2 h, followed by addition of a saturated sodium bicarbonate solution to a pH of 7.5–8. Chloroform was added, and the aqueous phase was extracted with CHCl_3_ 3x. The combined organic phase was washed with a saturated sodium bicarbonate solution and brine, then dried with sodium sulfate. The organic phase was purified by flash chromatography, eluting with 15% CMA in CHCl_3_ to give (−)-**38** (0.125 g, 375 µmol, 30.2%). ^1^H NMR (400 MHz; CD_3_OD): δ 7.55 (d, *J* = 8.6 Hz, 1H), 7.25–7.22 (m, 2H), 7.17–7.12 (m, 3H), 6.67 (d, *J* = 2.6 Hz, 1H), 6.60 (dd, *J* = 8.6, 2.6 Hz, 1H), 5.71 (s, 1H), 4.84 (s, 1H), 3.34 (d, *J* = 3.0 Hz, 1H), 2.86 (dt, *J* = 11.6, 5.7 Hz, 1H), 2.79–2.68 (m, 2H), 2.65–2.61 (m, 1H), 2.47 (td, *J* = 13.0, 6.0 Hz, 1H), 2.00–1.91 (m, 2H), 1.85 (td, *J* = 12.8, 4.5 Hz, 1H), 1.34 (s, 3H), 0.80 (d, *J* = 7.0 Hz, 3H). ^13^C NMR (101 MHz, CD_3_OD): δ 159.35, 143.52, 141.46, 138.12, 129.69, 129.44, 128.04, 127.12, 124.76, 114.74, 113.21, 112.05, 67.35, 58.22, 47.30, 43.23, 43.21, 38.37, 34.53, 26.05, 14.61. HRMS: [C_23_H_28_NO]^+^ Calcd: 334.2171; found: 334.2165. The -(−)-**38**•HCl salt was formed by dissolving the free base in a minimal amount of acetone. The solution was concentrated to a pale syrup, and diethyl ether was added. The mixture was stirred overnight to give (−)-**38**•HCl as a pale beige solid. Anal. Calcd. For C_23_H_28_ClNO • 0.4 H_2_O C, 73.25; H, 7.70; N, 3.71. Found: C, 73.24; H, 7.82; N, 3.57.
*(2R,6S,11S)-1,6,11-Trimethyl-3-phenethyl-1,2,3,4,5,6-hexahydro-2,6-methanobenzo[d]azocin-8-ol* (2*R*,6*S*,11*S*-(+)-**39**)**.** In a Parr Shaker flask, (+)-**38** (0.107 g, 1 equiv, 321 µmol), palladium on carbon (205 mg, 5% wt, 0.3 equiv, 96.3 µmol), and acetic acid (18.4 µL, 1 equiv, 321 µmol) were dissolved in methanol (10 mL) under an argon atmosphere. The vessel was charged with 30 psi of hydrogen gas and shaken overnight at room temperature. The reaction mixture was filtered through celite and rinsed multiple times with methanol. The filtrate was purified via flash chromatography to give (+)-**39** as a light brown foam (56 mg, 0.17 mmol, 52%). ^1^H NMR (400 MHz; CDCl_3_): δ 7.29–7.25 (m, 2H), 7.22–7.19 (m, 3H), 7.05 (d, *J* = 9.2 Hz, 1H), 6.76–6.73 (m, 2H), 3.22 (d, *J* = 2.3 Hz, 1H), 3.05–2.93 (m, 5H), 2.89 (dd, *J* = 12.2, 3.9 Hz, 1H), 2.26 (td, *J* = 12.6, 3.3 Hz, 1H), 2.19–2.13 (m, 1H), 2.11 (s, 2H), 1.95 (td, *J* = 13.3, 4.7 Hz, 1H), 1.37 (d, *J* = 7.6 Hz, 3H), 1.29 (s, 3H), 0.87 (d, *J* = 7.2 Hz, 3H). ^13^C NMR (101 MHz, CDCl_3_): δ 155.80, 141.25, 138.45, 130.41, 128.76, 128.61, 128.20, 126.57, 114.10, 112.31, 64.12, 55.48, 45.33, 40.38, 39.36, 36.13, 32.46, 25.47, 24.12, 15.36. HRMS: [C_23_H_30_NO]^+^ Calcd: 336.2327; Found: 336.2327. The free base was dissolved in a minimal amount of acetone, and 6 drops of concentrated HCl was added. Upon stirring, a syrup formed. Diethyl ether was added, and the mixture was stirred overnight to give (+)-**39**. HCl as an off-white solid (32 mg), mp 178–181 °C, [α]_25_^D^ = +70.7° (*c* 0.39, MeOH). Anal. Calcd. For C_23_H_30_ClNO • 0.75 H_2_O C, 71.67; H, 8.24; N, 3.63. Found: C, 71.83; H, 8.40; N:3.54.
*(2S,6R,11R)-1,6,11-Trimethyl-3-phenethyl-1,2,3,4,5,6-hexahydro-2,6-methanobenzo[d]azocin-8-ol* (2*S*,6*R*,11*R*-(−)-**39**). In a Parr Shaker flask, (−)-**38** (0.120 g, 1 equiv, 360 µmol), palladium on carbon (230 mg, 5% wt, 0.3 equiv, 108 µmol), and acetic acid (20.6 µL, 1 equiv, 360 µmol) were added to methanol (10 mL) under an argon atmosphere. The vessel was charged with 30 psi of hydrogen gas and shaken overnight at room temperature. The reaction mixture was filtered through celite and rinsed multiple times with methanol. The filtrate was purified via flash chromatography to give (−)-**39** as a light brown foam (71 mg, 0.21 mmol, 59%), ^1^H NMR (400 MHz; CDCl_3_): δ 7.31–7.27 (m, 2H), 7.27–7.20 (m, 3H), 7.07 (d, *J* = 8.2 Hz, 1H), 6.83–6.81 (m, 2H), 3.32 (s, 1H), 3.13–3.00 (m, 4H), 3.00–2.94 (m, 1H), 2.41–2.35 (m, 2H), 2.16–2.08 (m, 2H), 1.40 (d, *J* = 7.6 Hz, 3H), 1.33 (s, 2H), 0.89 (d, *J* = 7.2 Hz, 3H). ^13^C NMR (101 MHz, CDCl_3_): δ 155.84, 140.71, 137.58, 128.78, 128.74, 128.31, 126.86, 114.42, 112.38, 64.56, 55.44, 45.69, 39.72, 38.76, 35.96, 31.86, 25.19, 24.08, 15.24. HRMS: [C_23_H_30_NO]^+^ Calcd: 336.2327; Found: 336.2326. The free base was dissolved in a minimal amount of acetone, and 6 drops of concentrated HCl was added. Diethyl ether was added to the syrup that was obtained, and the mixture was stirred overnight to give (−)-**39**•HCl as an off-white solid (57 mg), mp 181–184 °C, [α]_25_^D^ –69.1° (c 0.59, MeOH). Anal. Calcd. For C_23_H_30_ClNO • 0.75 H_2_O C, 71.67; H, 8.24; N, 3.63. Found: C, 71.73; H, 8.34; N, 3.48.

### 3.3. X-ray Crystal Structure Experimental Data

Single-crystal X-ray diffraction data on compound (+)-**34** were collected using Cu Kα radiation and a Bruker SMART APEX II CCD area detector. The crystal was prepared for data collection by coating with high-viscosity microscope oil. The oil-coated crystal was mounted on a micromesh mount (MiTeGen, Inc., Ithaca, NY, USA) and transferred to the diffractometer, and a data set collected at 293(2) K. The 0.097 × 0.074 × 0.060 mm^3^ crystal was monoclinic in space group P2_1_ with unit cell dimensions a = 11.2668(6) Å, b = 7.8053(4) Å, c = 13.0428(6) Å, *α* = *γ* = 90° and *β* = 97.890(2)°. The final anisotropic full matrix least-squares refinement on F^2^ with 272 variables converged at R_1_ = 5.68% for the observed data and wR2 = 15.01% for all data. The structure was solved by direct methods and refined by full-matrix least-squares on F^2^ values using the programs found in the SHELXL suite (Bruker, SHELXL v2014.7, 2014, Bruker AXS Inc., Madison, WI, USA). Corrections were applied for Lorentz, polarization, and absorption effects. Parameters refined included atomic coordinates and anisotropic thermal parameters for all nonhydrogen atoms. The H atoms were included using a riding model and direct assignment. The hydrogen atom found on the solvent methanol (O25) required a DFIX command to prevent migration. This is likely due to very minor positional disorder that could not be modeled to completion. Stereochemistry of C1 (R), C5 (S) and C9 (S) was determined via the orientation of the molecules. Complete information on data collection and refinement is available in the Supplementary Materials, Tables S1–S7.

Atomic coordinates for (+)-**34** have been deposited with the Cambridge Crystallographic Data Centre, deposition number 2290877. Copies of the data can be obtained, free of charge, on application to CCDC, 12 Union Road, Cambridge, CB2 1EZ, UK (fax: +44(0)-1223-336033 or e-mail: deposit@ccdc.cam.ac.uk).

### 3.4. In Vitro Assay

#### 3.4.1. Cell Lines and Cell Culture

Cell lines and cell culture: cAMP Hunter^TM^ Chinese hamster ovary cells (CHO-K1) that express human μ-opioid receptor (OPRM1), human κ-opioid receptor (OPRMK1), and human δ-receptor (OPRMD1) were purchased from Eurofins DiscoverX (Fremont, CA) and used for the forskolin-induced cAMP accumulation assays [16]. All cell lines were maintained in F-12 media with 10% fetal bovine serum (Life Technologies, Grand Island, NY, USA), 1% penicillin/streptomycin/l-glutamine (Life Technologies), and 4.800 µg/mL Geneticin (Mirus Bio, Madison, WI, USA). All cells were grown at 37 °C and 5% CO_2_ in a humidified incubator.

#### 3.4.2. Forskolin-Induced cAMP Accumulation Assays

Assays were performed as previously described [30]. Briefly, 10,000 cells/well of cells were plated in 384-well tissue culture plates and incubated overnight at 37 °C in 5% CO_2_. Stock solutions of the compound were prepared in DMSO at 5 mM concentration, and then 9 to 10 concentrations of 100X working solutions were prepared by serial dilution using DMSO. 5X working solutions were subsequently prepared using assay buffer consisting of Hank’s Buffered Salt Solution, HEPES, and forskolin. For the agonist assay, cells were incubated at 37 °C with compounds for 30 min at a 1X final concentration. For the antagonist assay [28], cells were incubated at 37 °C with compounds for 15 min before 30 min incubation at 37 °C with the selected agonist at their EC_50_ or EC_90_ dose. Detection was performed by using the HitHunter cAMP Assay for Small Molecules by DiscoverX according to the manufacturer’s directions, and the BioTek Synergy H1 hybrid plate reader (BioTek, Winooski, VT, USA) and Gen5 Software version 2.01 were used to quantify luminescence (BioTek, Winooski, VT, USA). To determine % efficacy in forskolin-induced cAMP assays, data were blank subtracted with the vehicle control, followed by normalization to the forskolin control. Data were then analyzed in GraphPad Prism 8 (GraphPad, LaJolla, CA, USA) using nonlinear regression. Values are expressed as the mean ± SEM of at least three independent experiments. Degree of antagonism (I_max_) was normalized to naltrexone (MOR, DOR) or *nor*-BNI (KOR).

## 4. Conclusions

Highly potent MOR agonists were found with electron withdrawing (e.g., NO_2_, (−)-**3**, EC_50_ = 0.3 nM, % E_max_ = 101%) and electron donating (e.g., OH, (−)-**20**, EC_50_ = 0.13 nM, % E_max_ = 102%) substituents at the *para* position in the aromatic ring of the *N*-phenethyl moiety. An *ortho*-positioned substituent (e.g., F, (−)-**8**) was also extremely potent (EC_50_ = 0.2 nM, % E_max_ = 101%). The *meta*-substituted compounds were in general much less potent than their comparable *ortho*- and *para*-analogs with one exception—the *meta*-fluoro analog (−)-**7** (EC_50_ = 0.6 nM, % E_max_ = 100%) was more potent than the *para*-fluoro analog (−)-**6** (EC_50_ = 1.8 nM, % E_max_ = 100%). A clear pattern between agonist potency and electron donating or withdrawal effects was not observed.

Steric effects were not clearly observable since potent compounds were found with both more bulky and less bulky substituents. Compounds with bulky *ortho* and *meta* substituents (e.g., **4** and **5**) were generally less potent than a compound with a comparable *para* substituent (**6**). The bulkier substituents—the 4-nitro ((−)-**3**) and 4-bromo ((−)-**12**) moieties, were potent, as were the less bulky 4-hydroxy ((−)-**20**) and 4-chloro ((−)-**15**) substituents. Addition of a second chloro atom in the aromatic ring ((−)-**21** and (−)-**22**) increased possible steric interactions and decreased potency—the monochloro substituent at the *ortho* (−)-**1**7 or *para* (−)-**15** position was at least three-fold more potent than the dichloro compounds.

A remarkable specificity of the chain length of the *N*-phenethyl moiety was observed. A single extra methylene changed the highly potent *N*-phenethyl compound (−)-**23** (EC_50_ = 0.27, %E_max_ = 101%) into a relatively inactive *N*-phenylpropyl compound (−)-**30** (EC_50_ = 70 nM, %E_max_ = 101%). The synthesis of levorotatory C8-substituted *N*-phenethylnormetazocines gave potent compounds, with the C8-methylene-substituted *N*-phenethylnormetazocine having subnanomolar potency. All the potent C8-substituted compounds were fully efficacious in vitro. These data could be summarized by noting that most substituents on the aromatic ring in the phenethyl moiety did not increase potency at MOR when compared with the unsubstituted aromatic ring in **23**. All of the substituents that were examined reduced potency, with only two exceptions, and those only slightly increased the potency of the unsubstituted compound **23**. Similarly, there were no major effects observable at DOR and KOR from compounds with the added substituents when compared with the unsubstituted compound **23**. Although the examined substituents on the aromatic ring in the *N*-phenethyl side chain were, with only two minor exceptions, unable to increase MOR potency or efficacy, the length of that side chain was of extreme importance—the addition of a single methylene group, to form an *N*-phenylpropyl side chain, resulted in over a 250-fold reduction in potency at MOR.

## Data Availability

The data presented in this study are available in this article or in the Supplementary Materials.

## Supplementary Materials

The following supporting information can be downloaded at: https://www.mdpi.com/article/10.3390/molecules28237709/s1, Figures S1–S40: ^1^H and ^13^C-NMR spectra of novel compounds, and Tables S1–S7: Crystal data, atomic coordinates from X-ray crystallographic determination of (+)-**34**.

## Abbreviations

MOR	mu-opioid receptor
DOR	delta-opioid receptor
KOR	kappa-opioid receptor
cAMP	cyclic adenosine monophosphate
cAMP assay	Inhibition of forskolin-induced cAMP accumulation assay
