# Supplementary material for: Potent MOR Agonists from 2′-Hydroxy-5,9-dimethyl-N-phenethyl Substituted-6,7-benzomorphans and from C8-Hydroxy, Methylene and Methyl Derivatives of N-Phenethylnormetazocine
Source: Molecules. 2023 Nov 22;28(23):7709. doi: 10.3390/molecules28237709 (PMC10708259; doi:10.3390/molecules28237709)
Supplement: Supplementary file 1 [file molecules-28-07709-s001.zip › molecules-2697473-supplementary.pdf]

Supplemental Materials

Potent MOR Agonists from 2'-Hydroxy-5,9-dimethyl-*N*-phenethyl substituted-6,7-benzomorphans and from C8-Hydroxy, Methylene and Methyl Derivatives of *N*-Phenethylnormetazocine

Madhurima Das [1], George W. Ward [1], Agnieszka Sulima [1], Dan Luo [2], Thomas Edward Prisinzano [2], Gregory H. Imler [3], Andrew T. Kerr [3], Arthur E. Jacobson [1,*] and Kenner C. Rice [1,*]

1 Drug Design and Synthesis Section, Molecular Targets and Medications Discovery Branch, Intramural Research Program, National Institute on Drug Abuse and the National Institute on Alcohol Abuse and Alcoholism, National Institutes of Health, Department of Health and Human Services, 9800 Medical Center Drive, Bethesda, MD 20892, USA; madhurima.das.91@gmail.com (M.D.); george.ward@fda.hhs.gov (G.W.W.); agnieszka.sulima@nih.gov (A.S.)

2 Department of Pharmaceutical Sciences, College of Pharmacy, University of Kentucky, 789 S. Limestone Street, Lexington, KY 40536, USA; dan.luo@uky.edu (D.L.); prisinzano@uky.edu (T.E.P.)

3 Center for Biomolecular Science and Engineering, Naval Research Laboratory, Washington, DC 20375, USA; greg.imler@gmail.com (G.H.I.); andrew.kerr@nrl.navy.mil (A.T.K.)

* Correspondence: arthurj@nida.nih.gov (A.E.J.); kennerr@nida.nih.gov (K.C.R.); Tel.: +1-301-451-5028 (A.E.J.); +1-301-451-4799 (K.C.R.)

Table of Contents

Figures S1-S40 ^{1}H and ^{13}C NMR S3-S42

Optical resolution of *rac*-**34** S34

X-ray crystallographic analysis data for 1*R*,5*S*,9*S*)-(+)-36.HBr S43-S50

^{1}H NMR and ^{13}C NMR of (–)-3

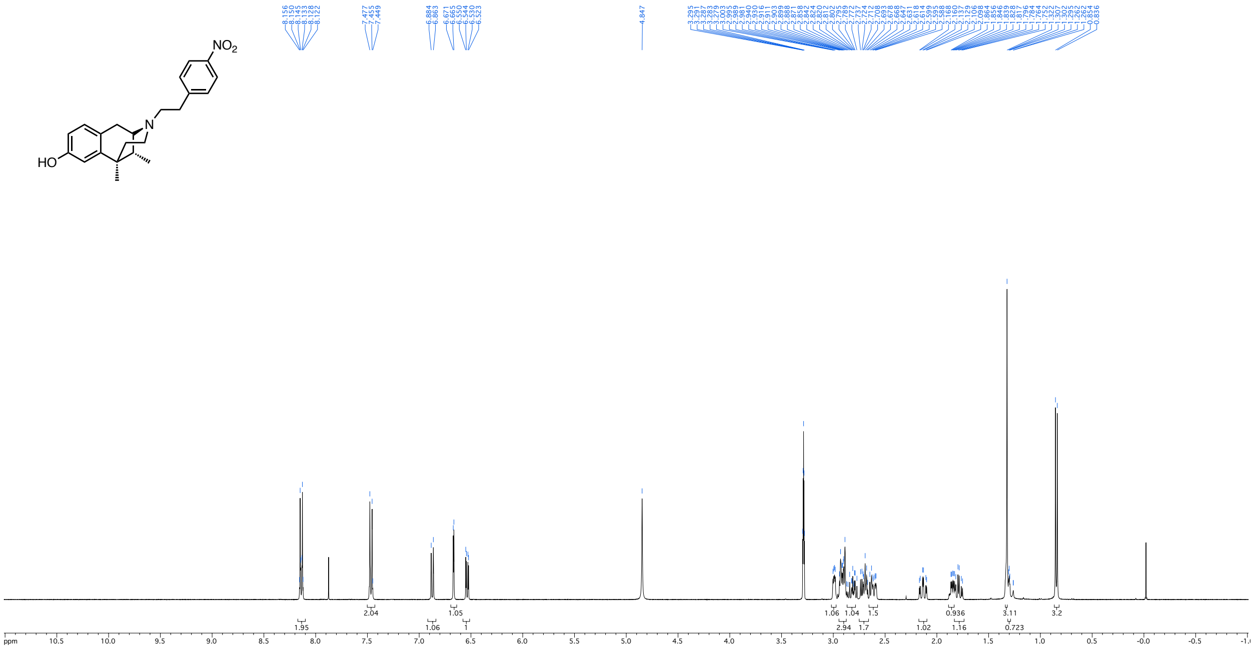

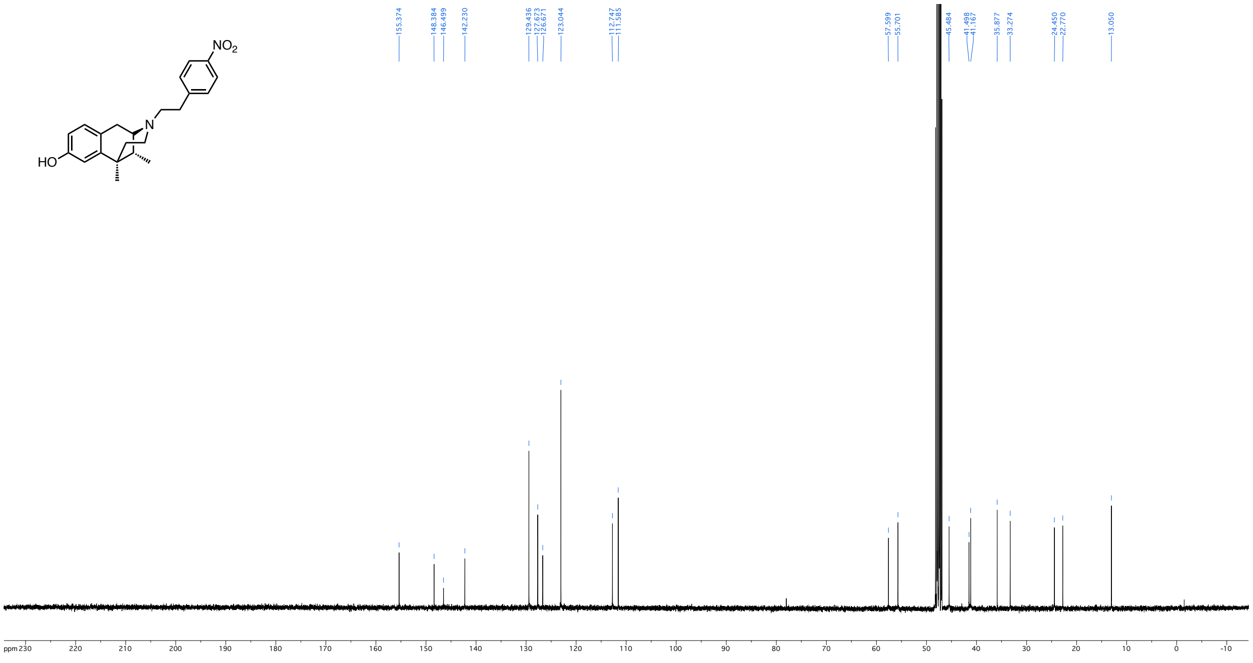

^{1}H NMR and ^{13}C NMR of (–)-4

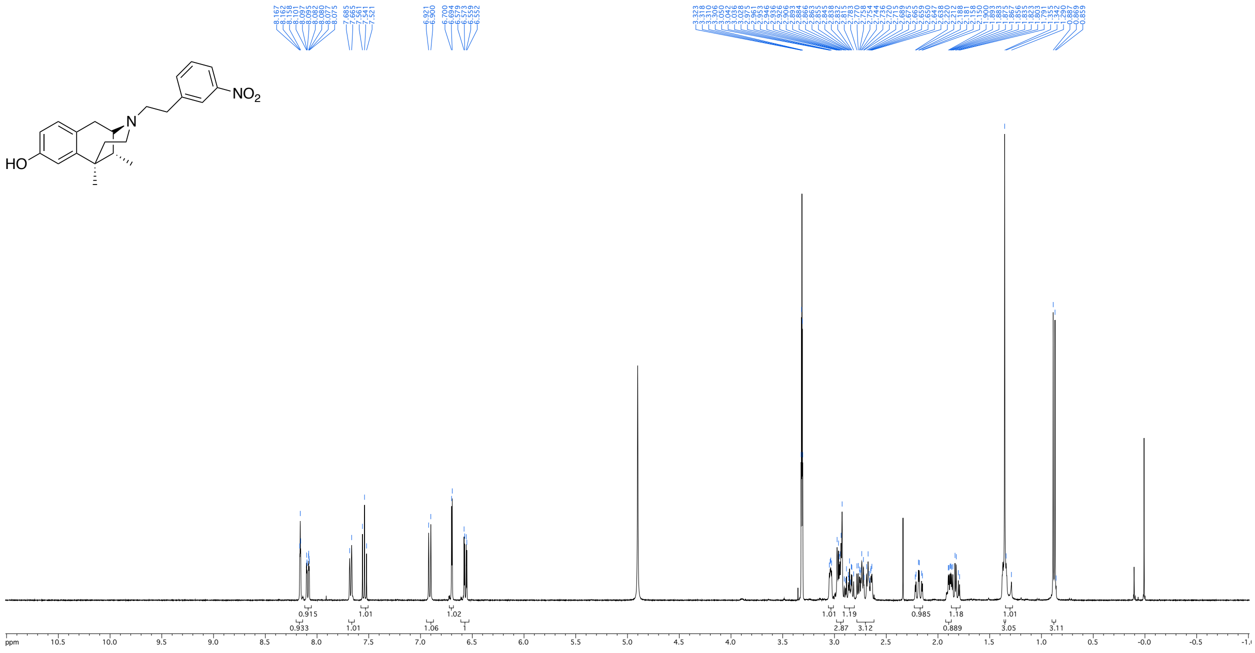

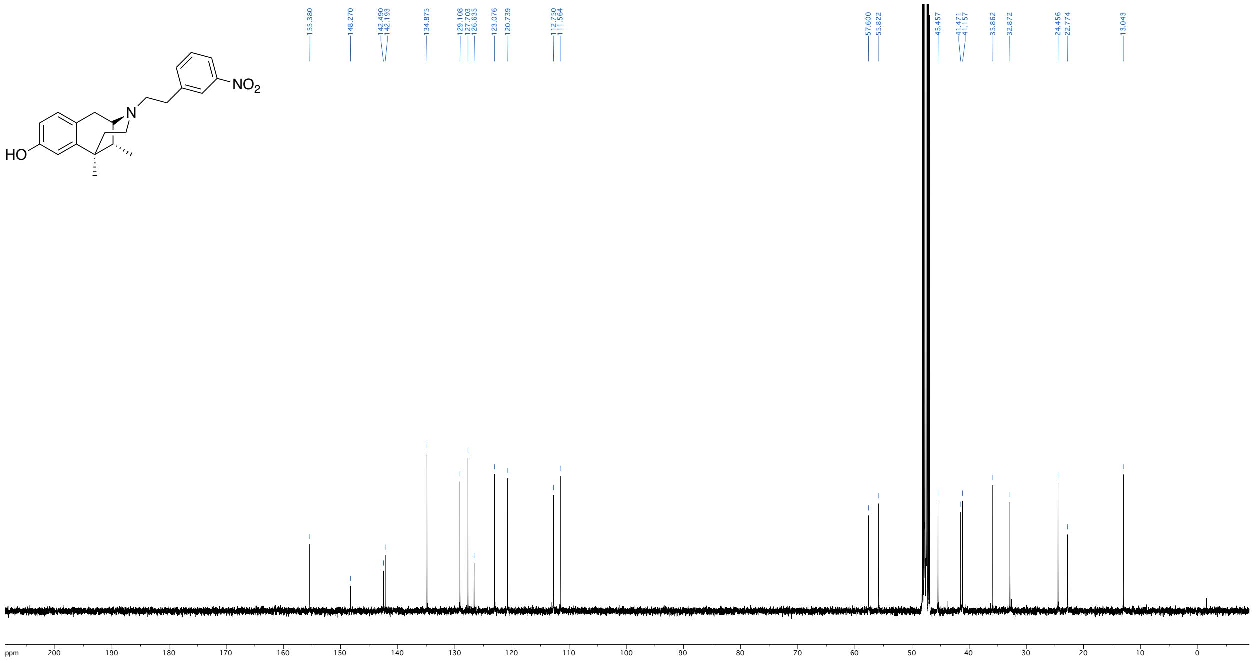

^{1}H NMR and ^{13}C NMR of (–)-5

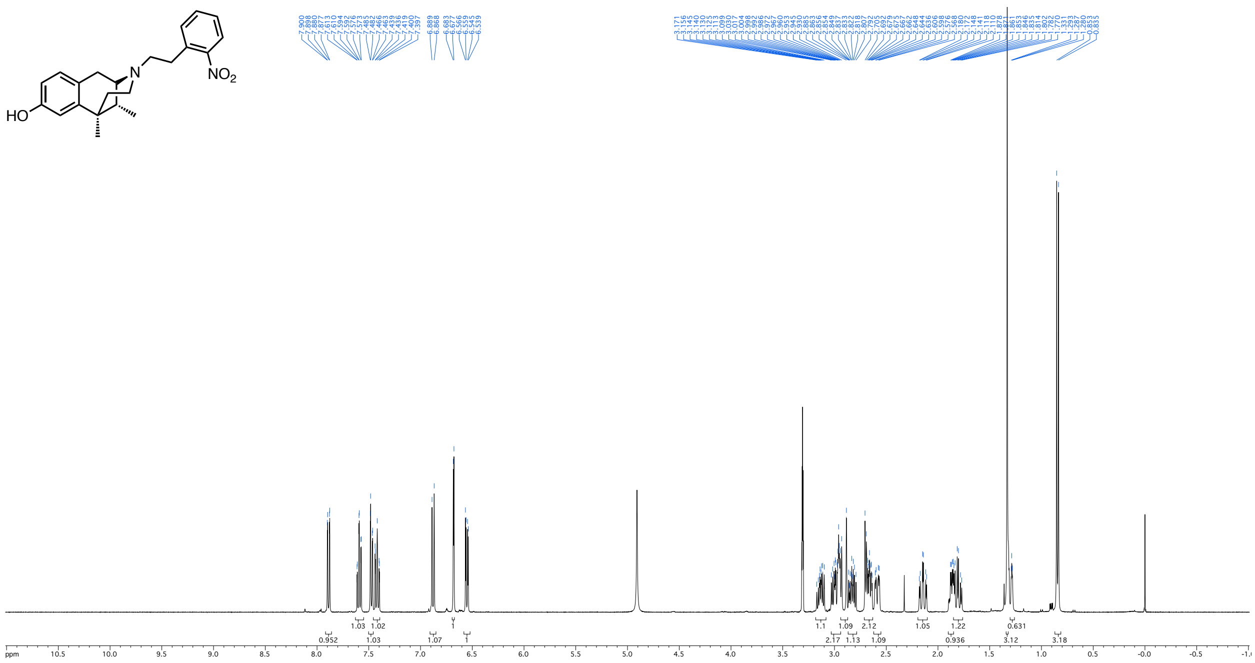

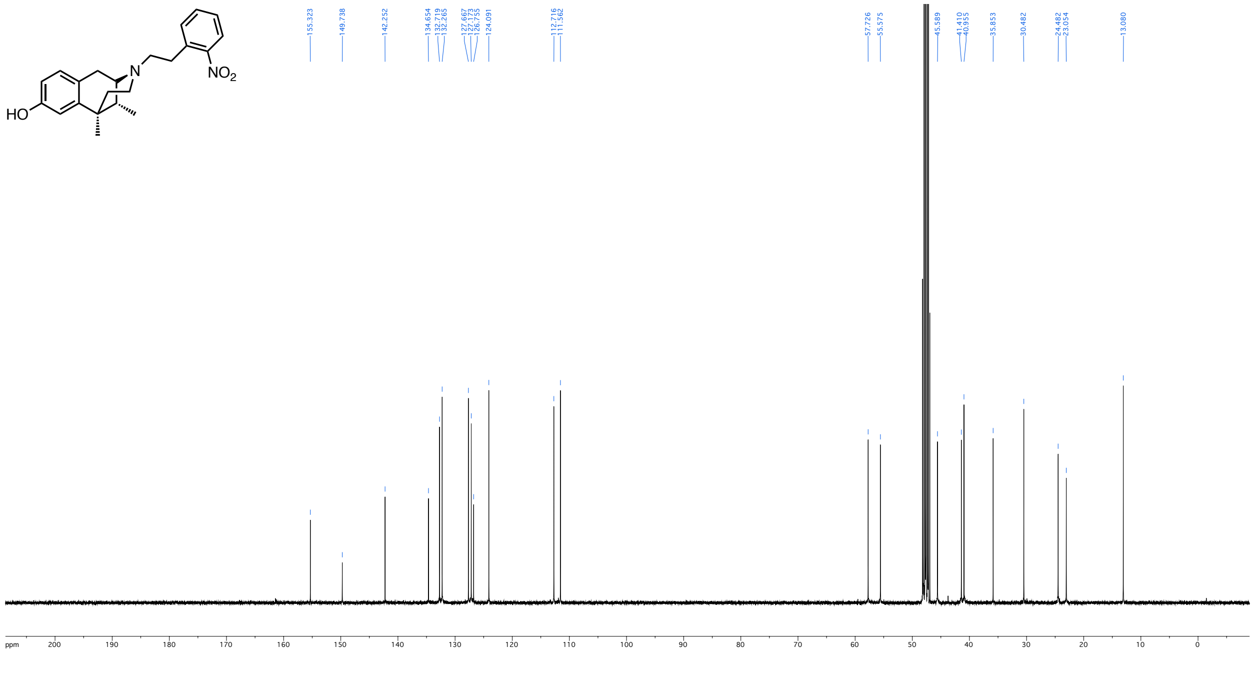

^{1}H NMR and ^{13}C NMR of (–)-6

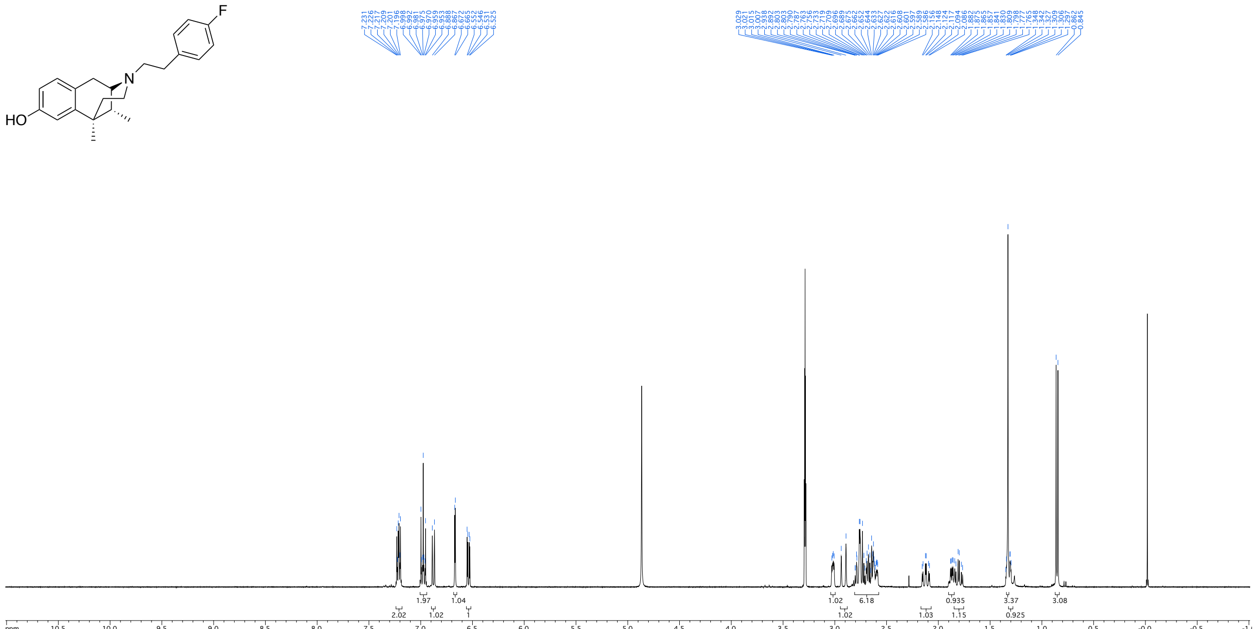

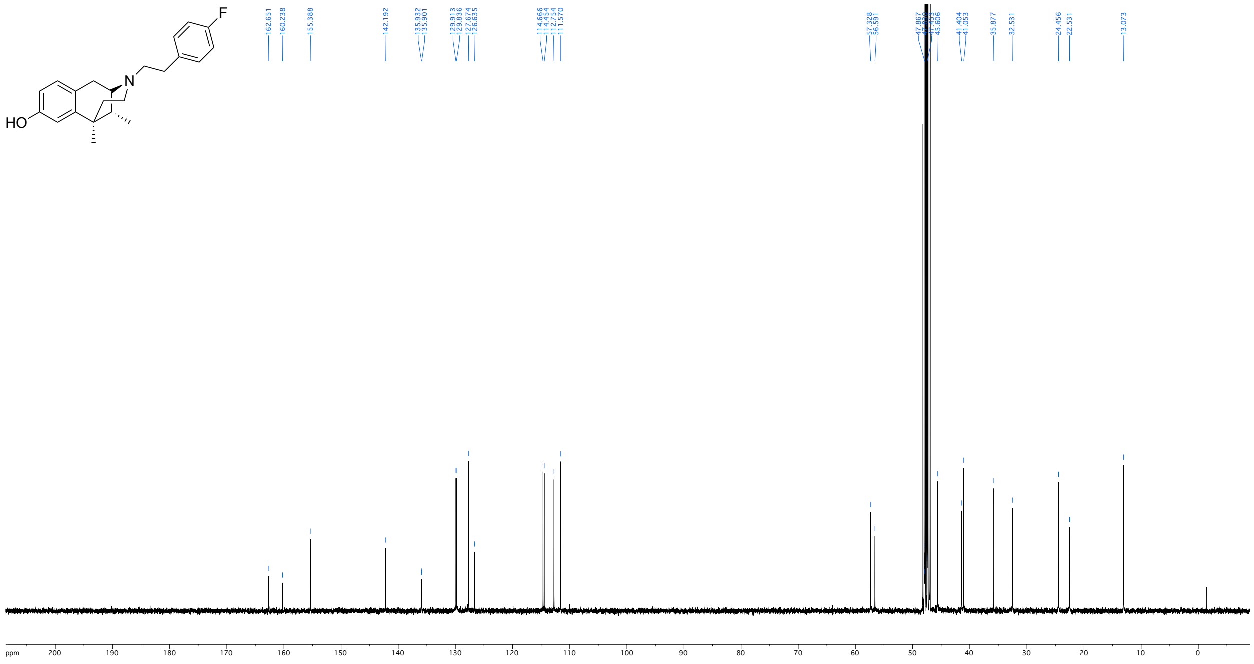

^{1}H NMR and ^{13}C NMR of (–)-7

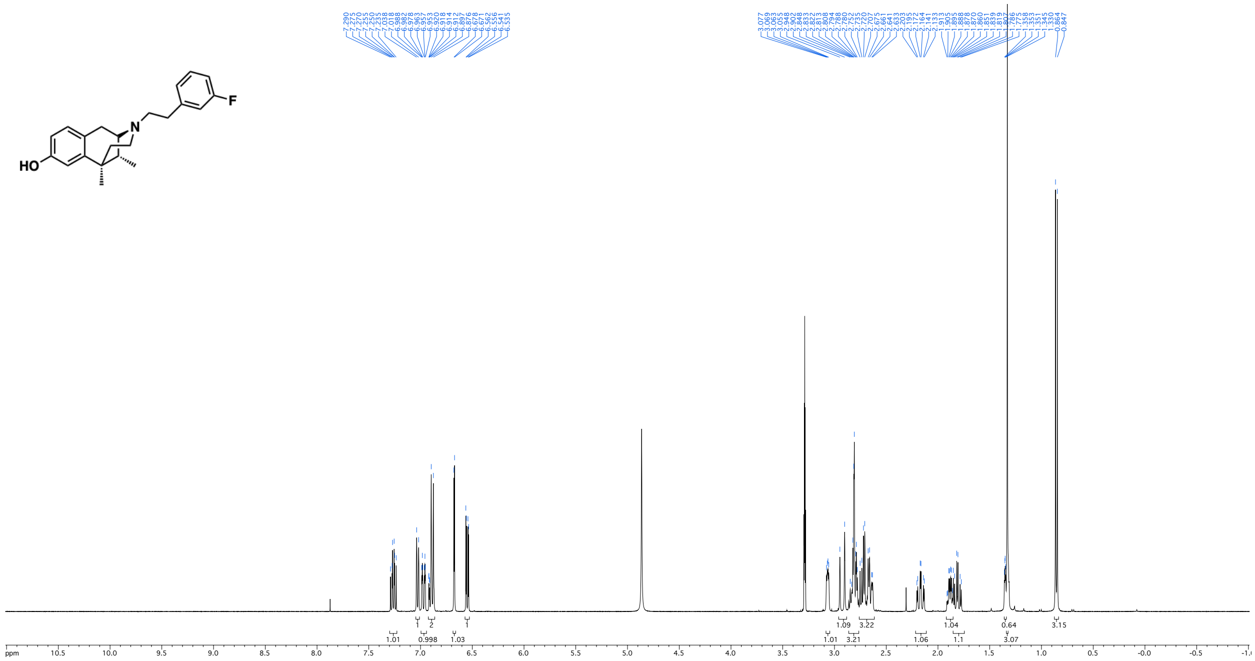

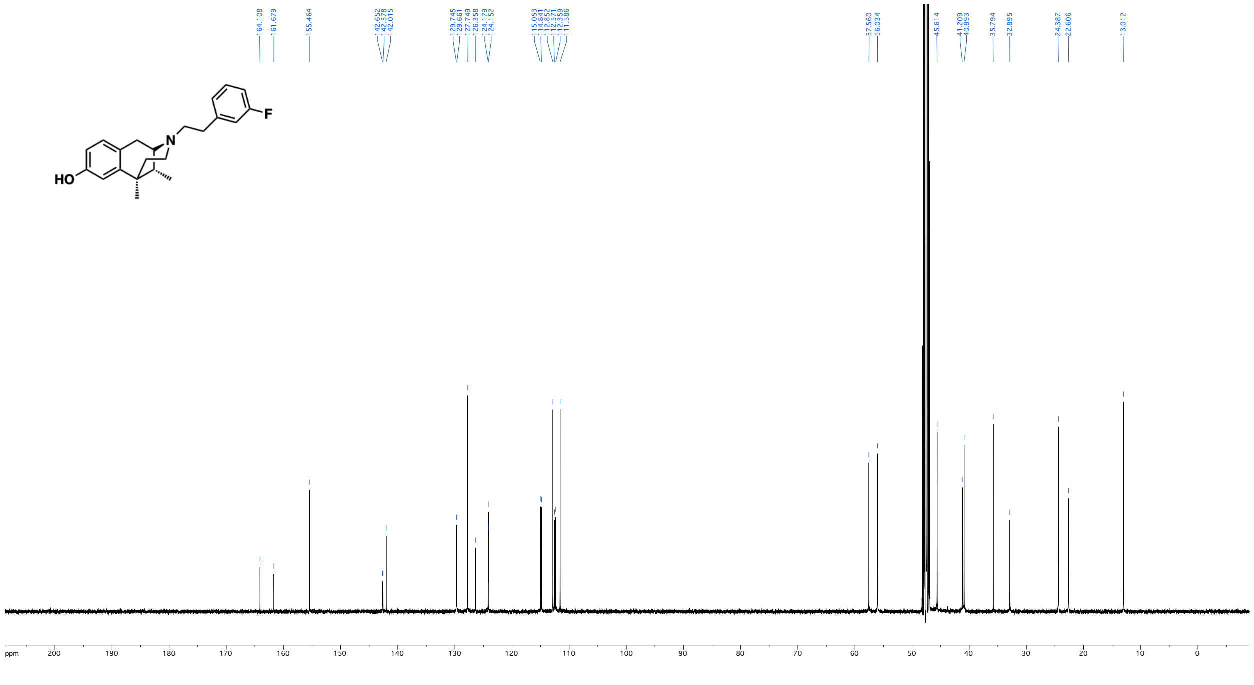

^{1}H NMR and ^{13}C NMR of (–)-8

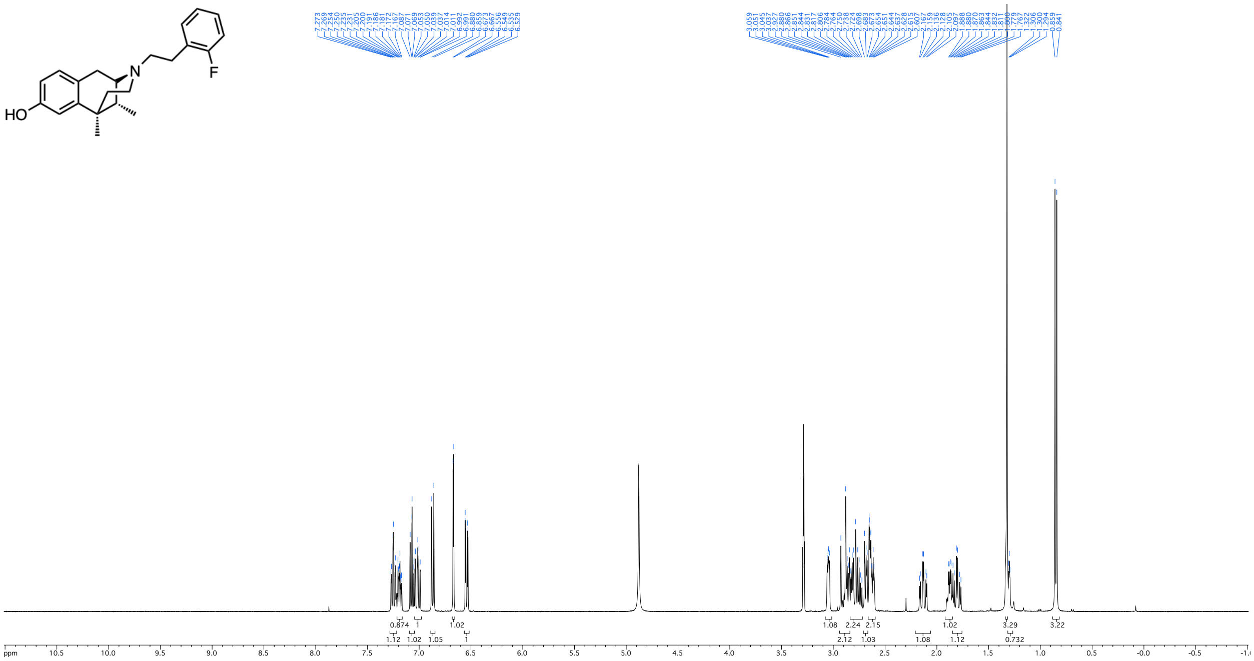

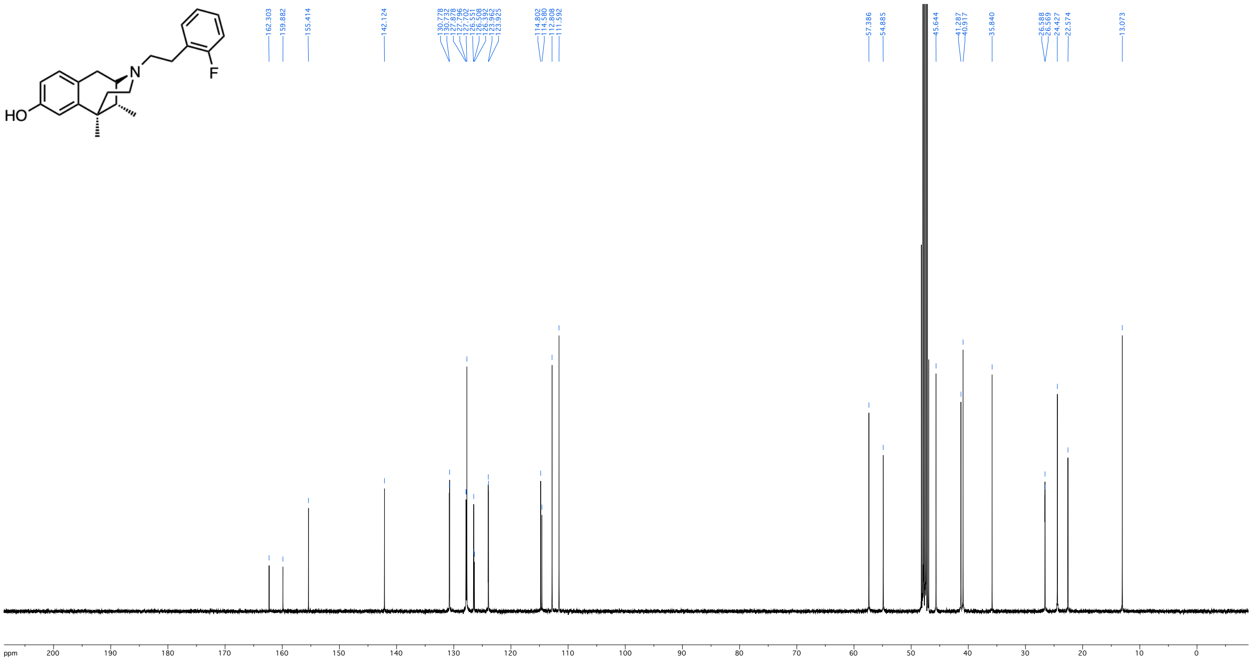

^{1}H NMR and ^{13}C NMR of (–)-9

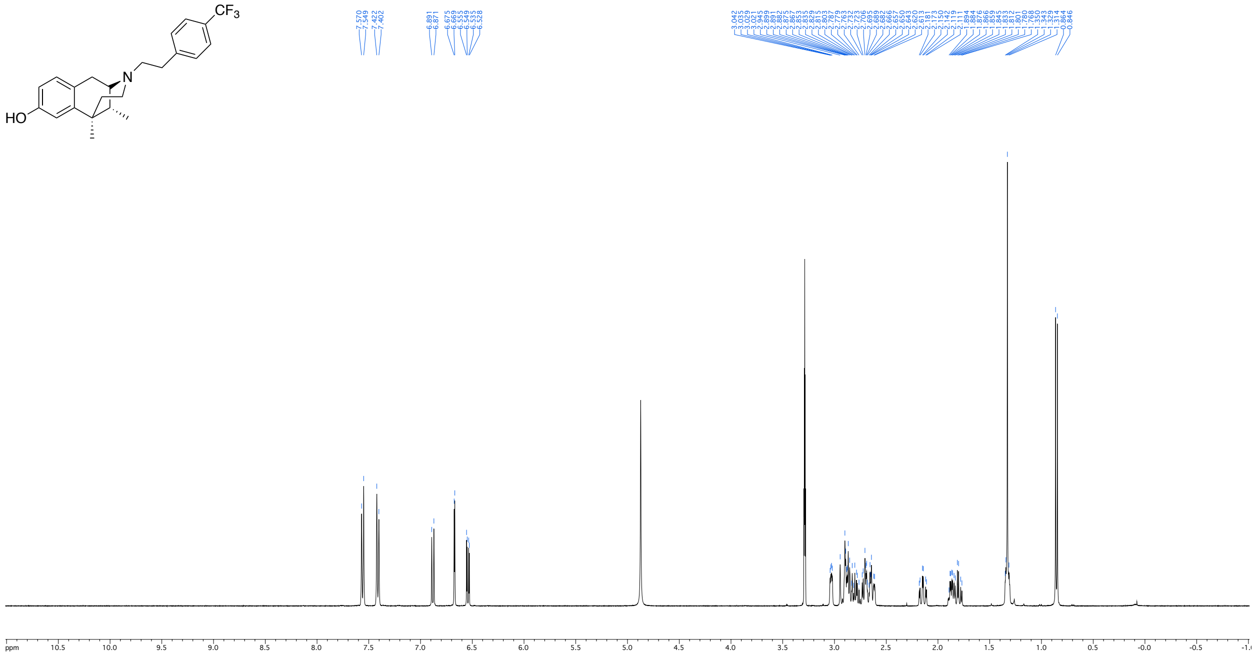

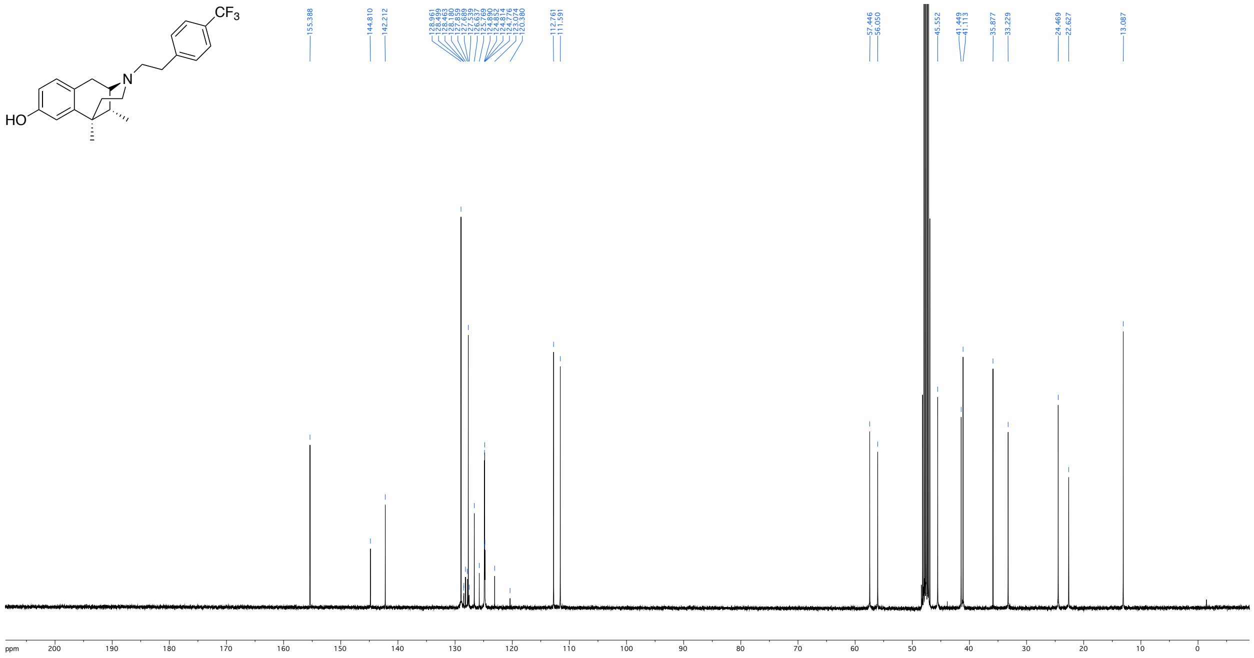

^{1}H NMR and ^{13}C NMR of (–)-10

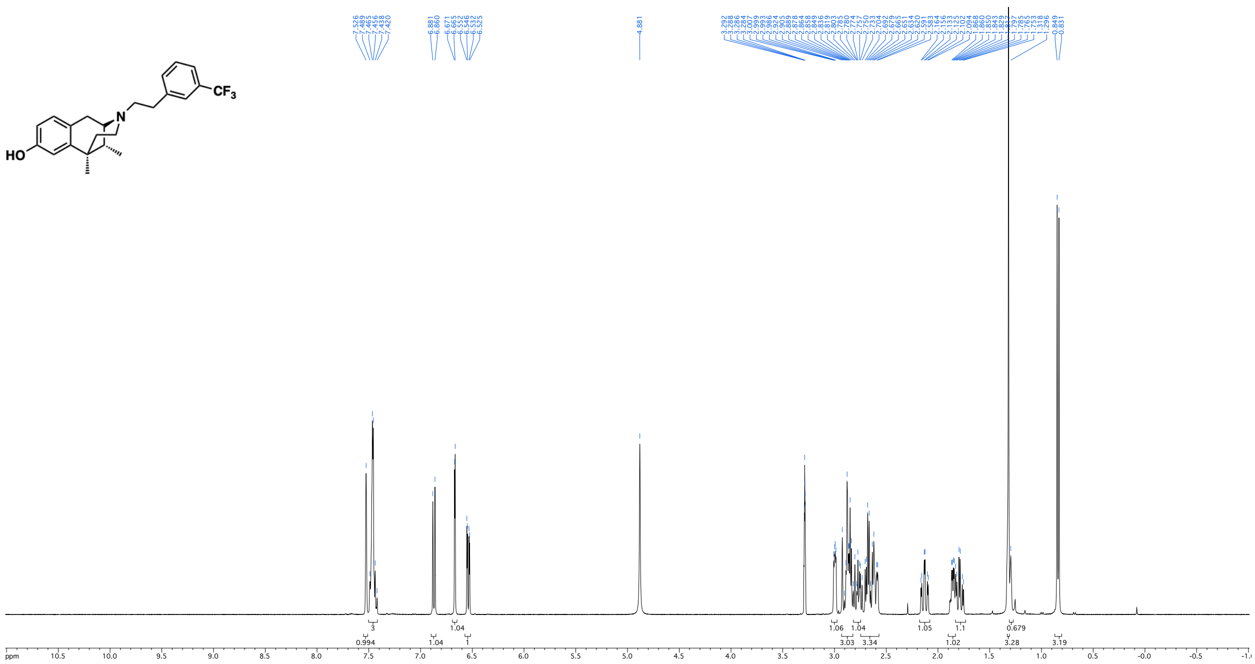

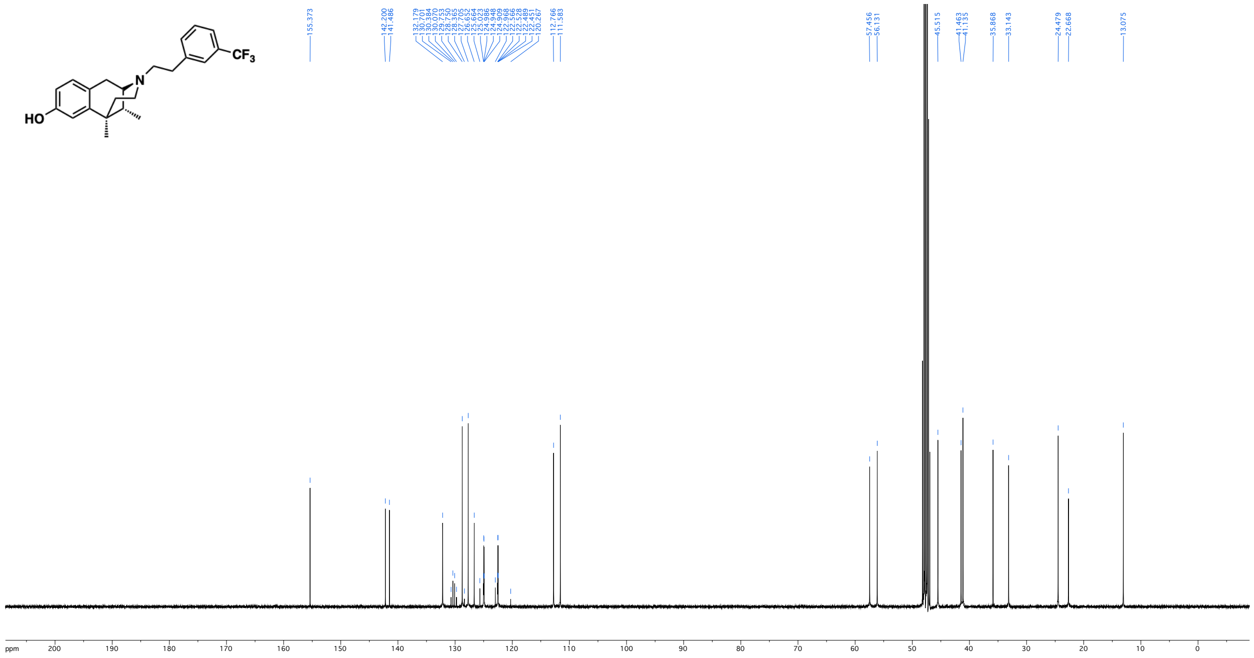

^{1}H NMR and ^{13}C NMR of (–)-11

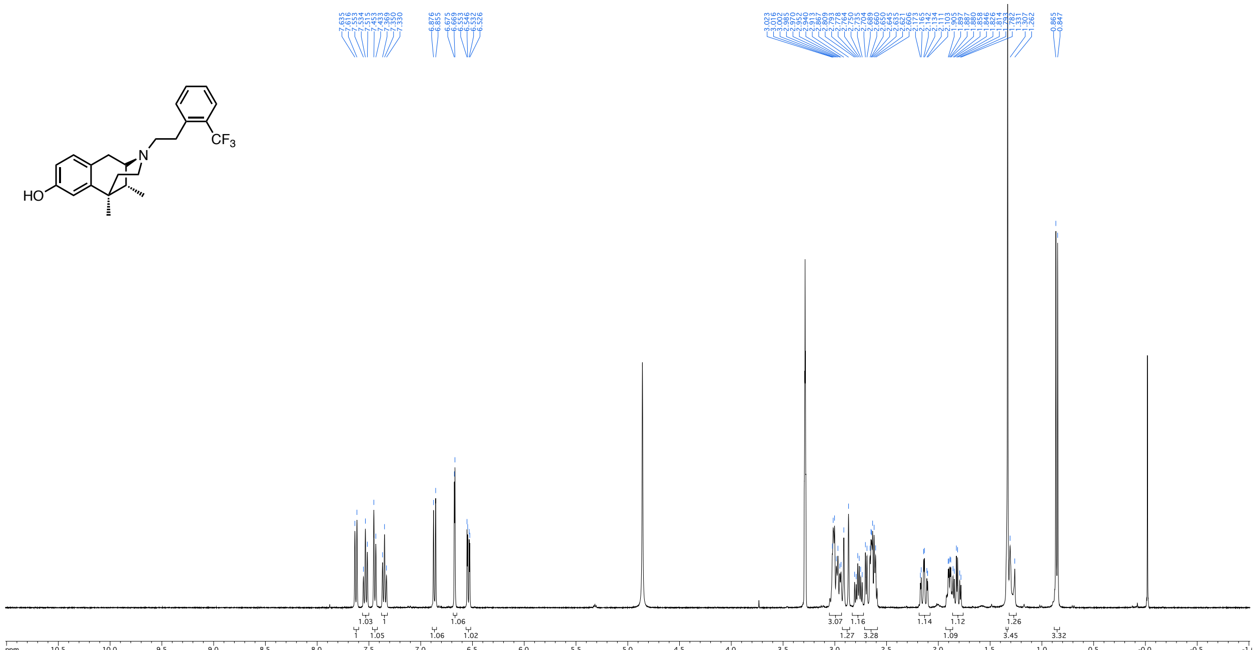

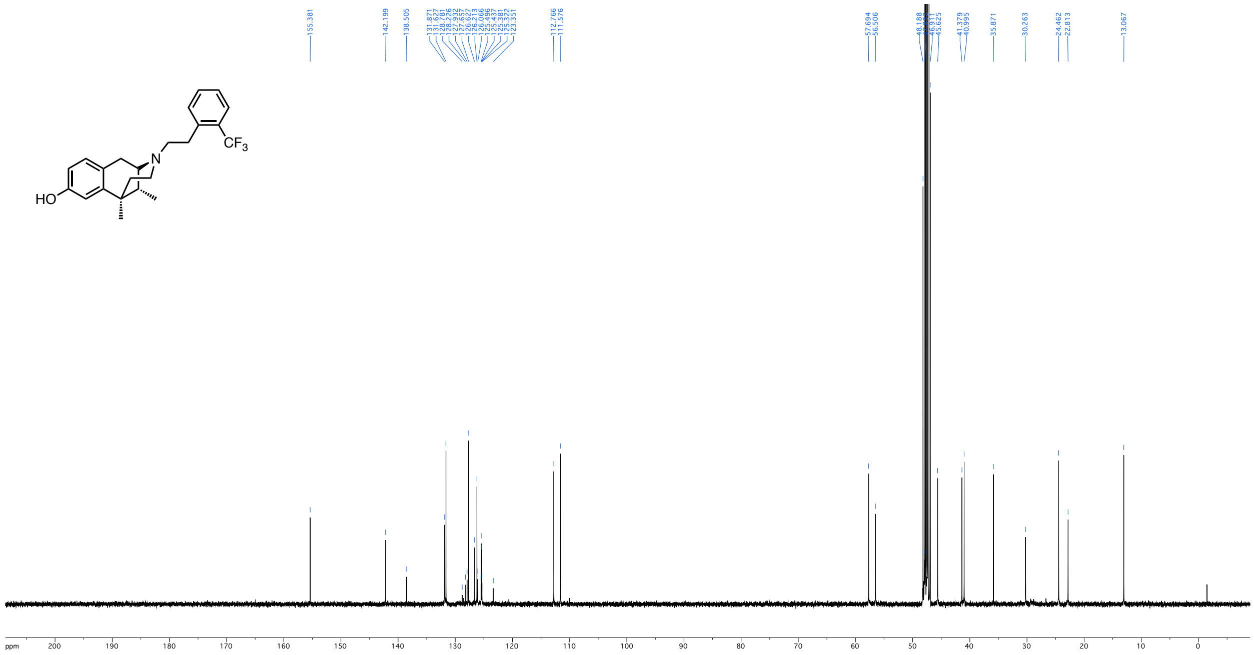

^{1}H NMR and ^{13}C NMR of (–)-12

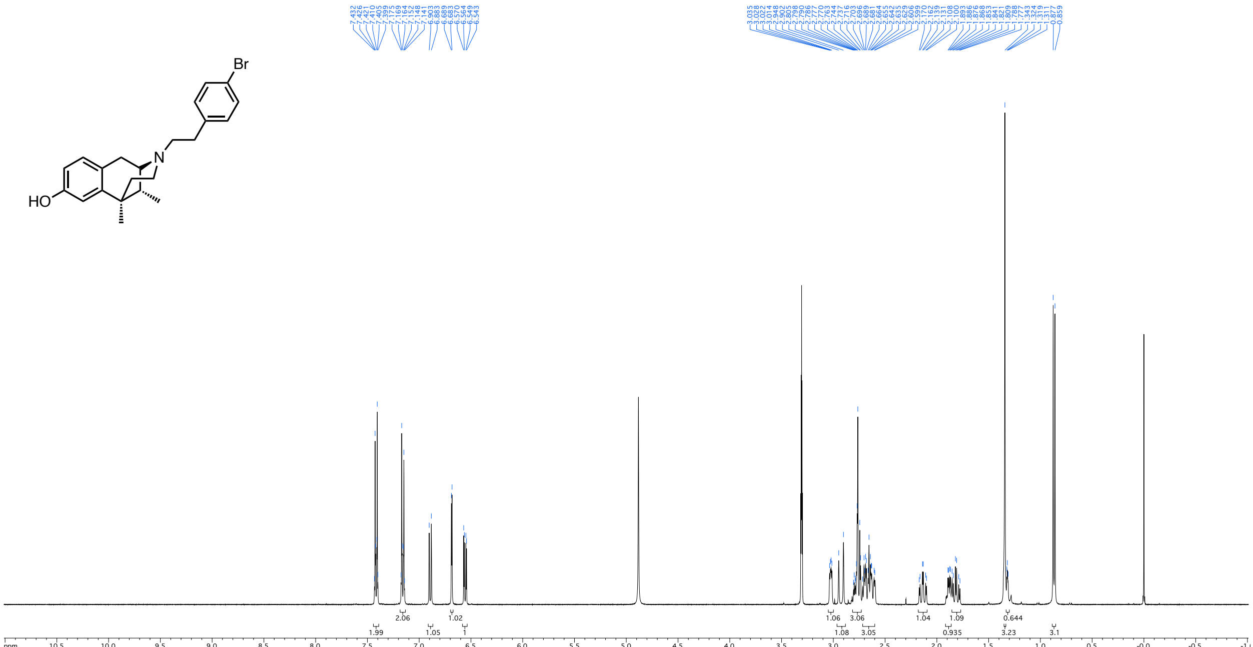

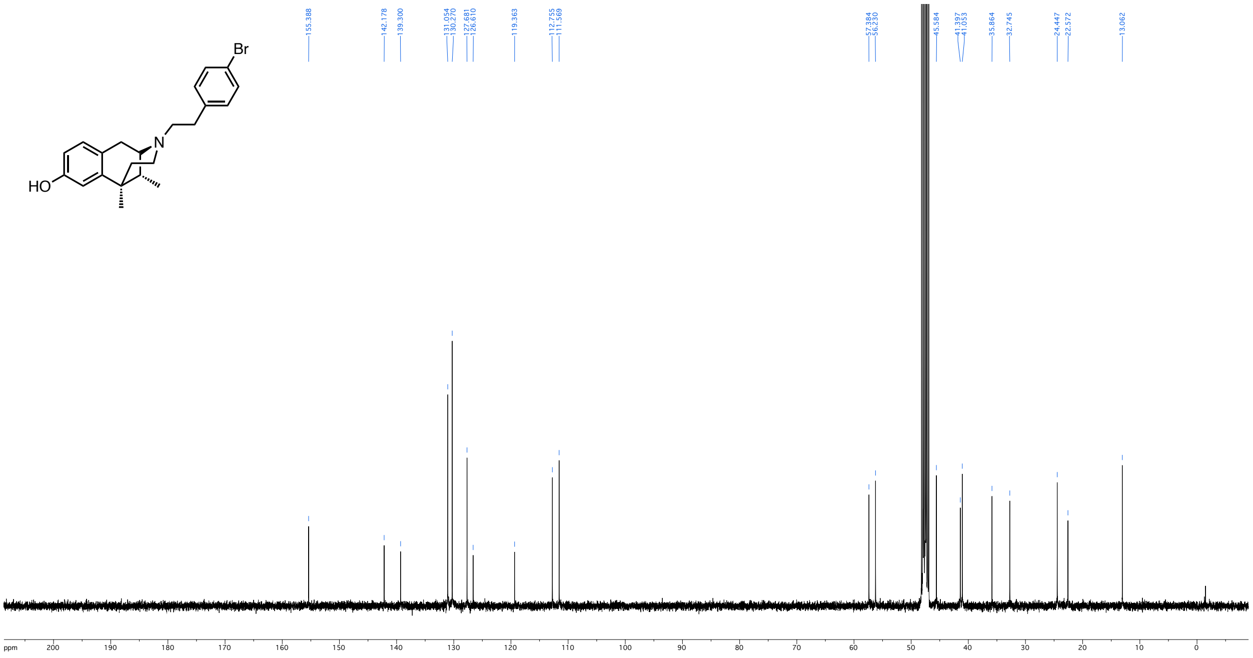

^{1}H NMR and ^{13}C NMR of (–-13

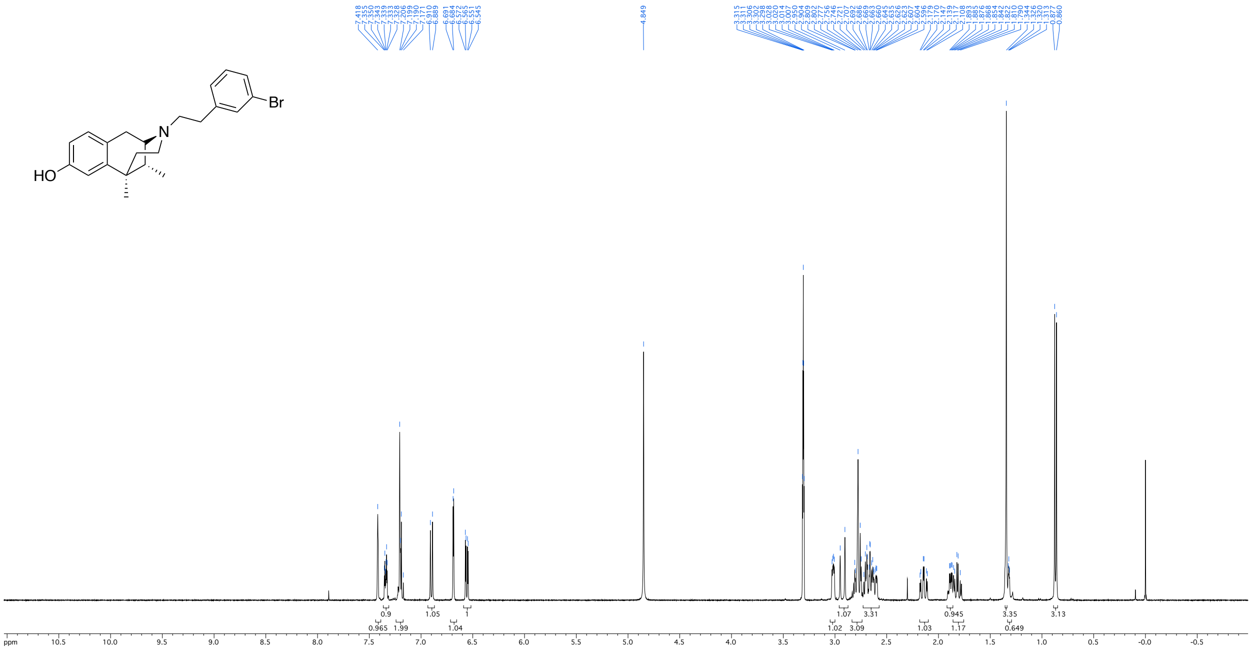

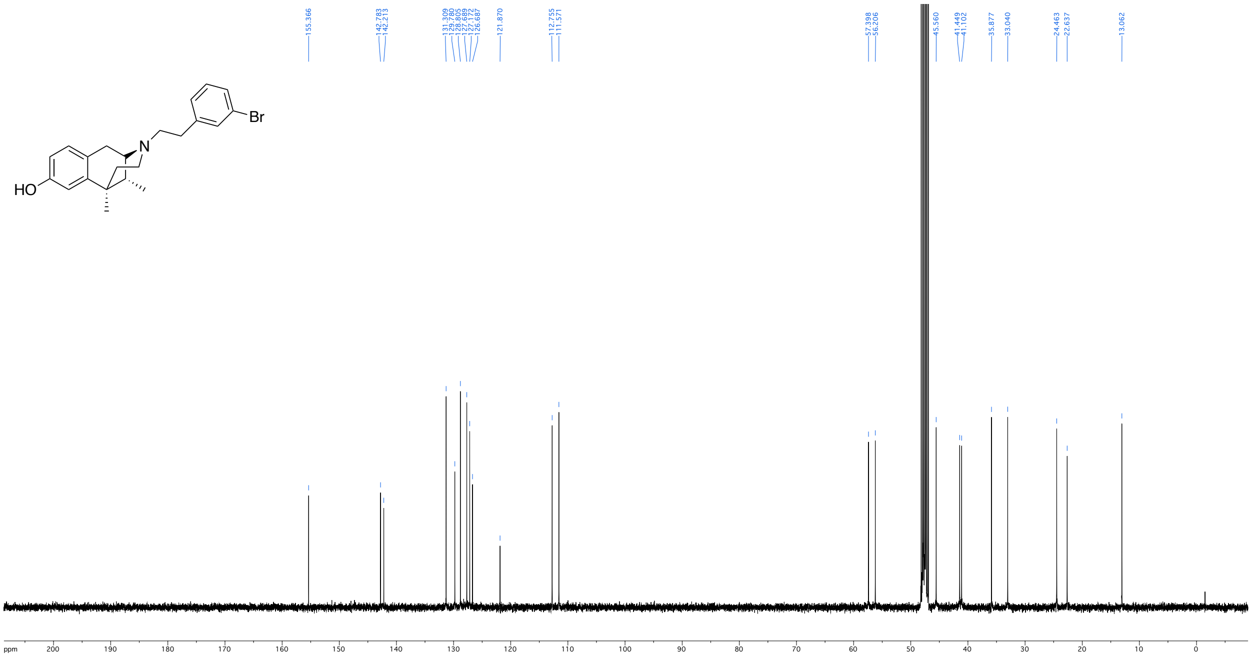

^{1}H NMR and ^{13}C NMR of (–)-14

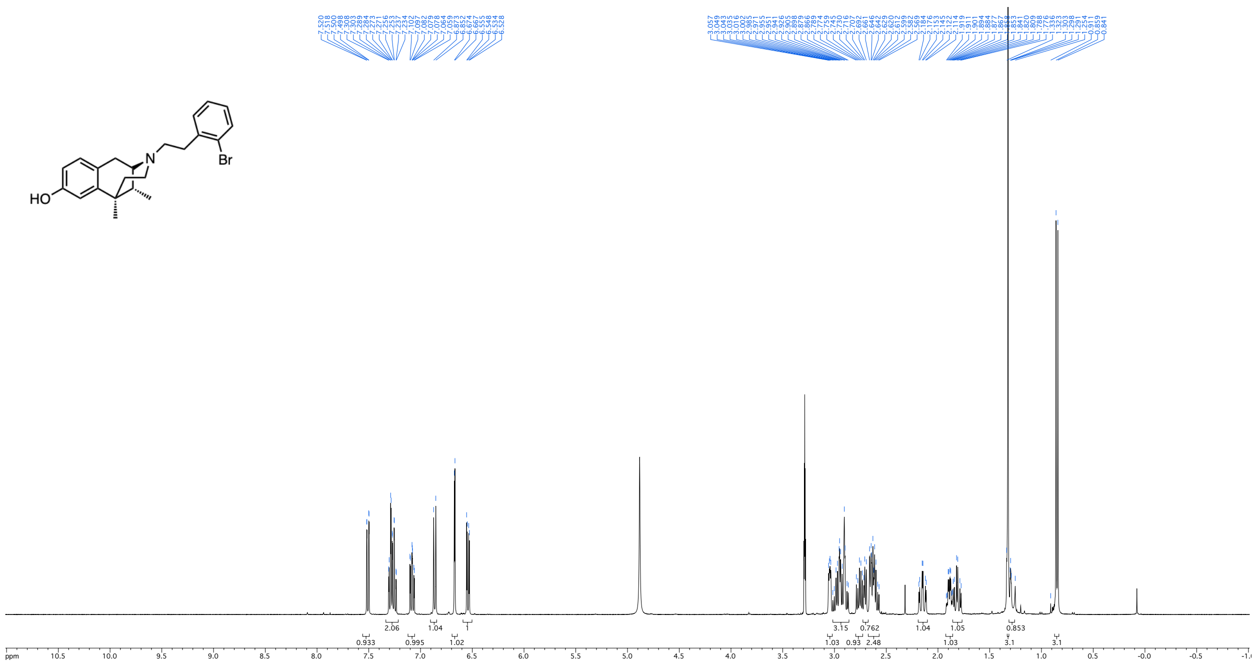

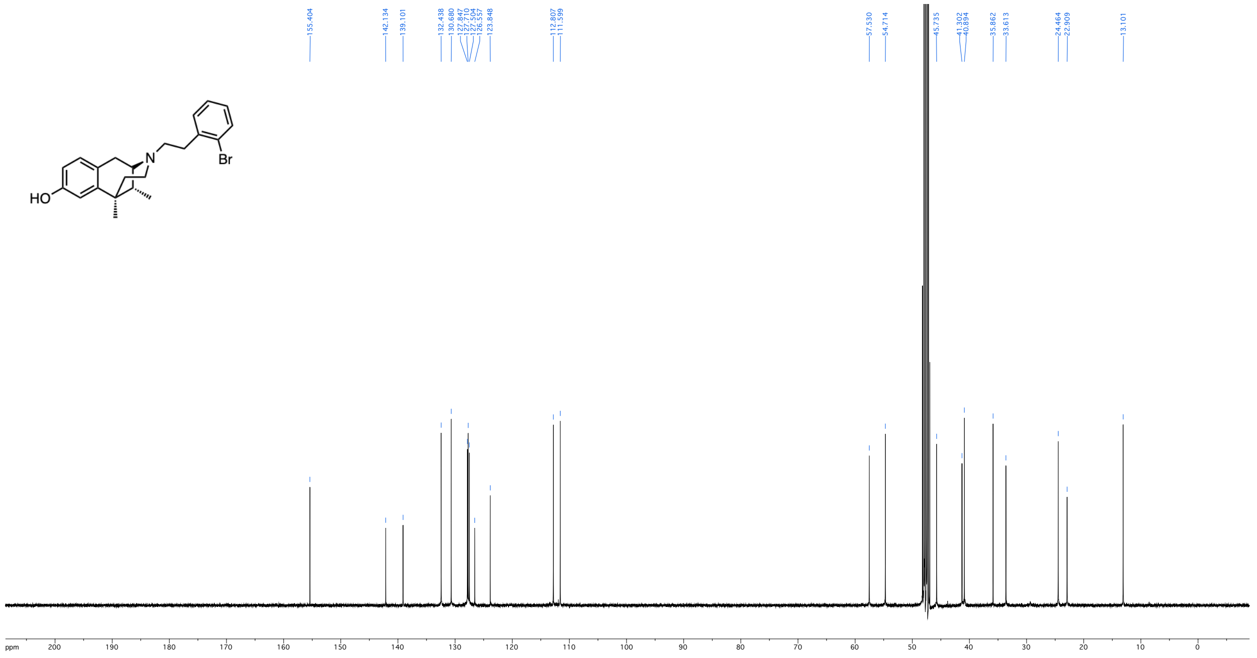

1H NMR and 13C NMR of (–)-15

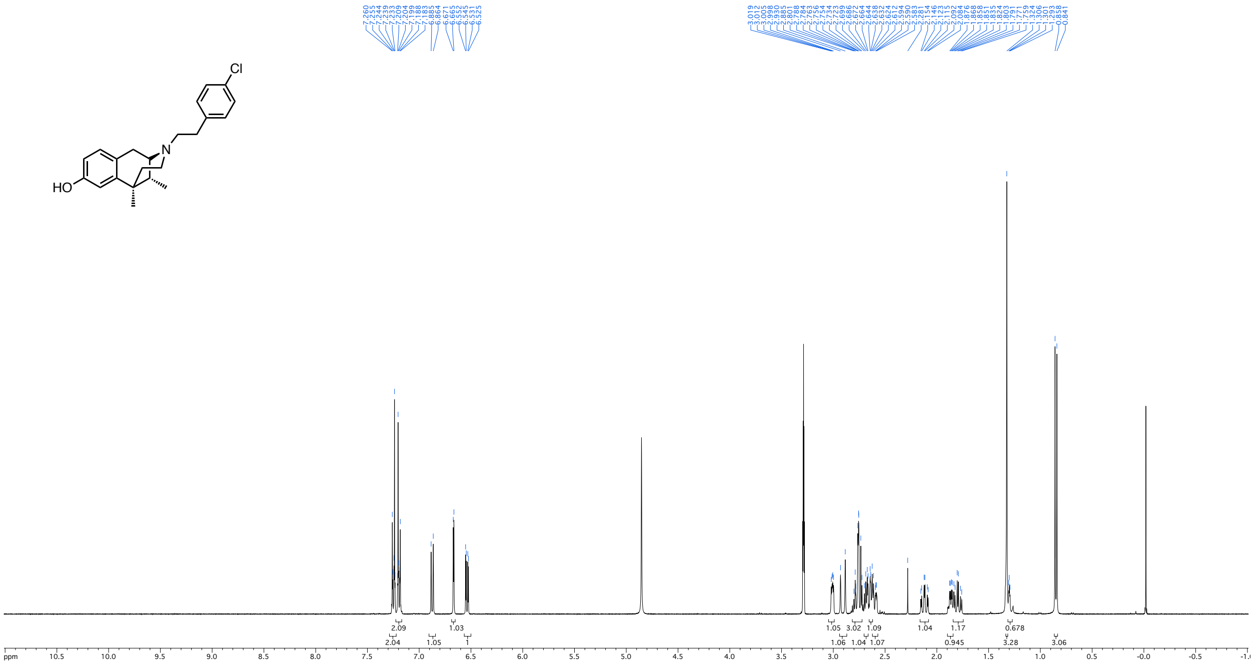

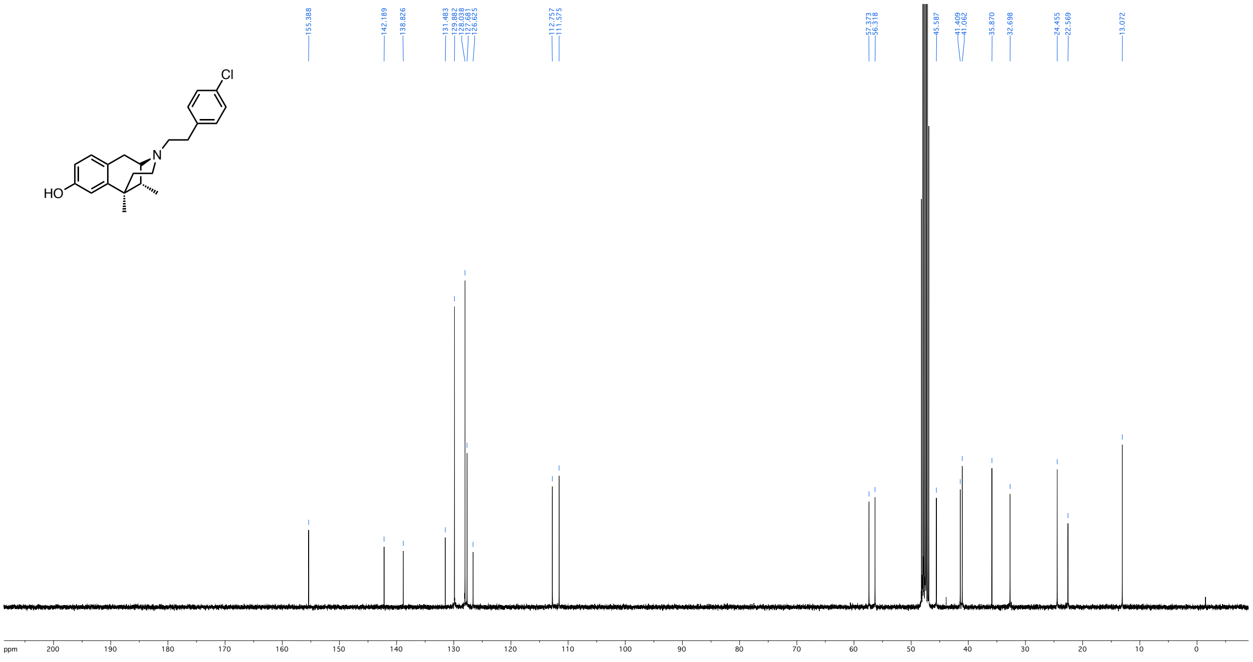

^{1}H NMR and ^{13}C NMR of (–)-16

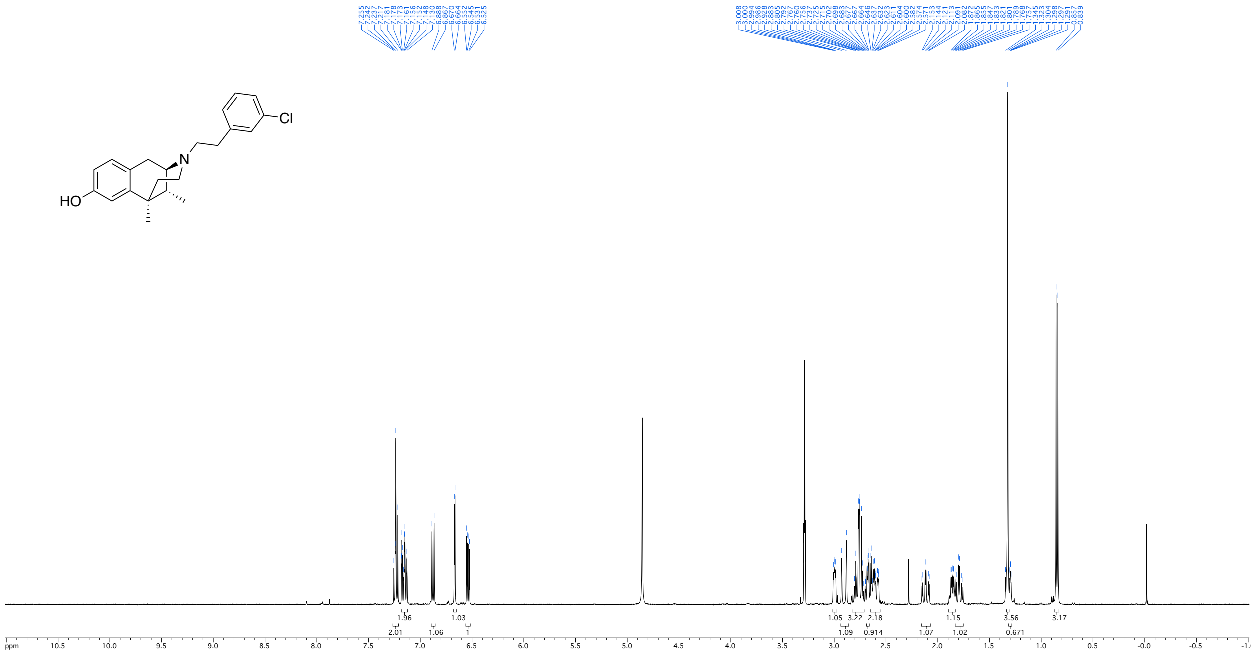

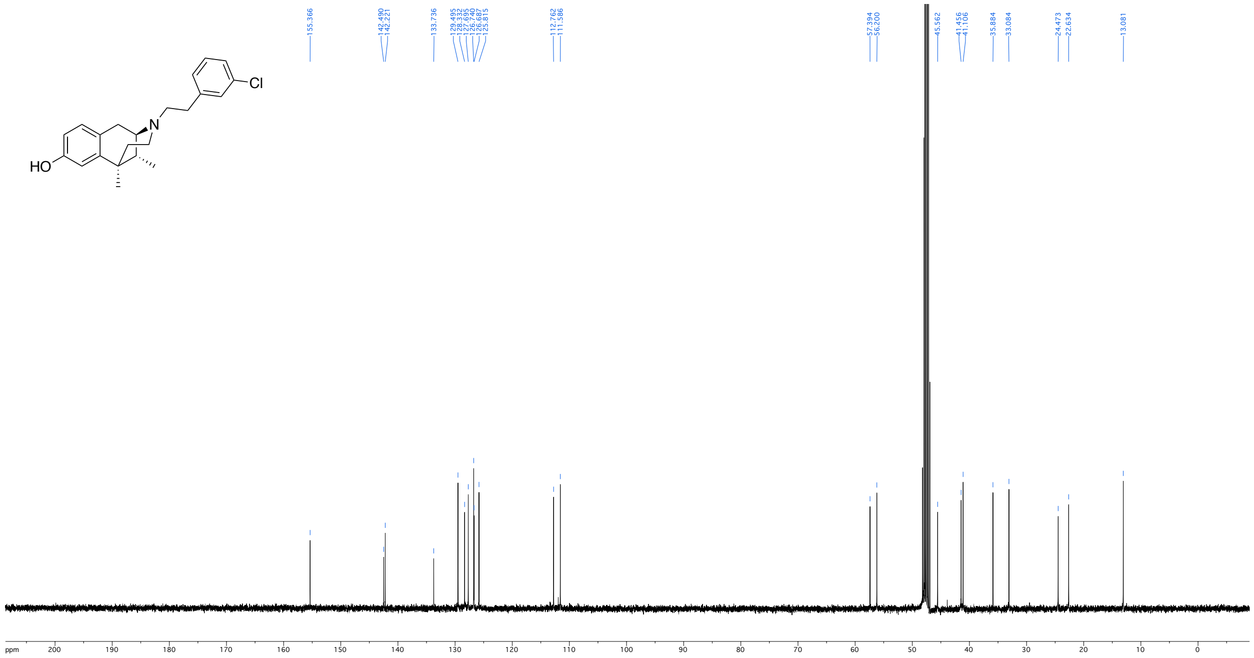

^{1}H NMR and ^{13}C NMR of (–)-17

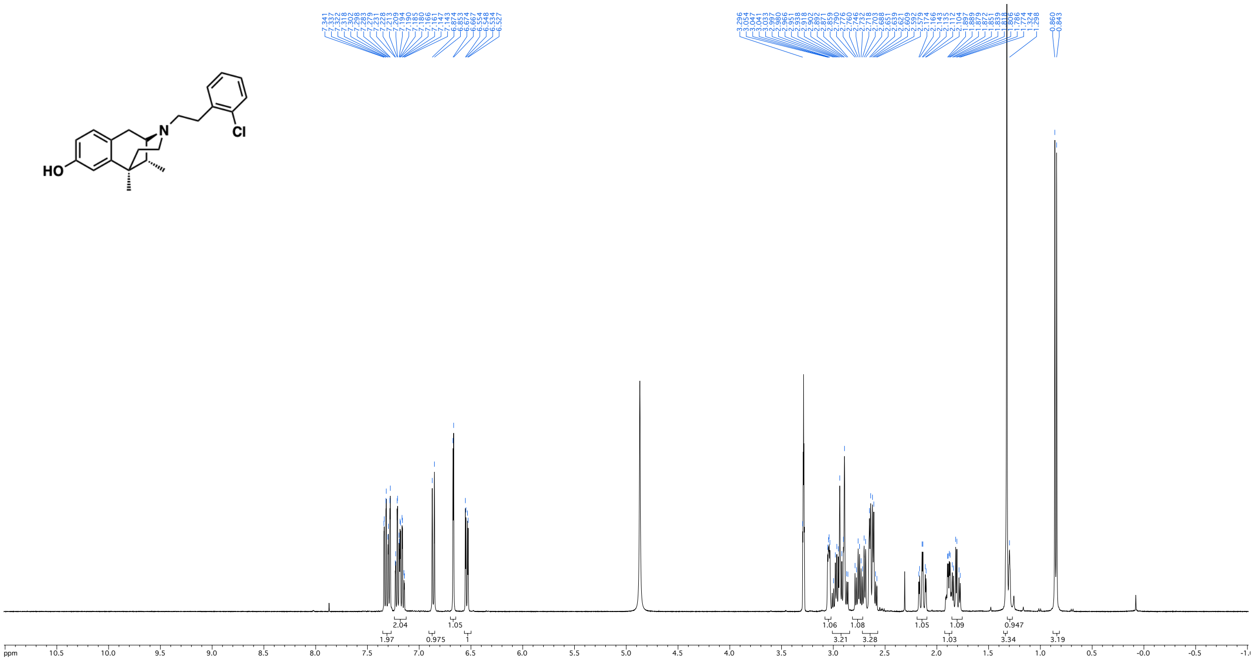

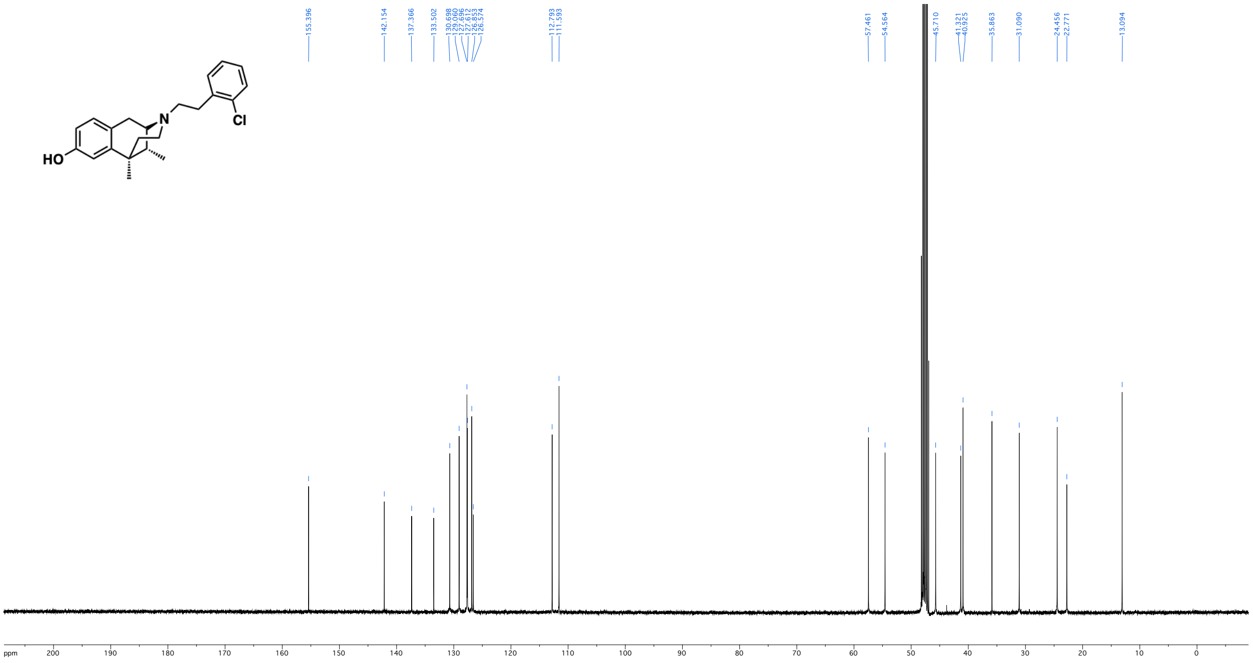

^{1}H NMR and ^{13}C NMR of (–)-18

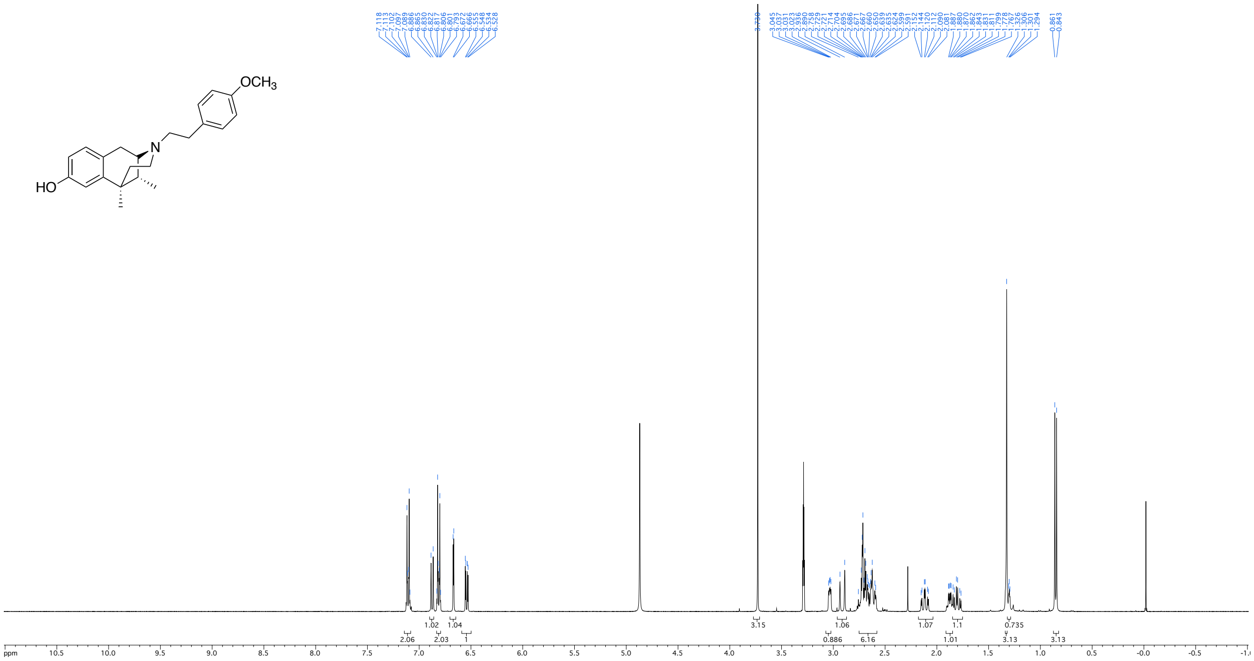

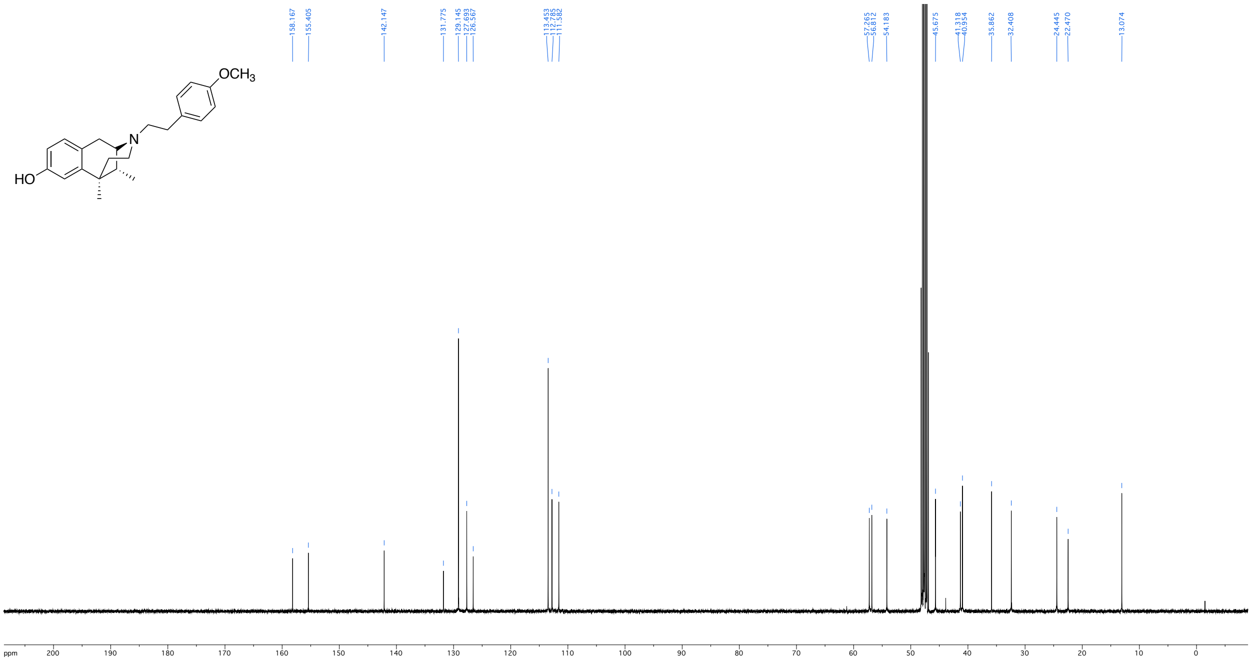

^{1}H NMR and ^{13}C NMR of (–)-19

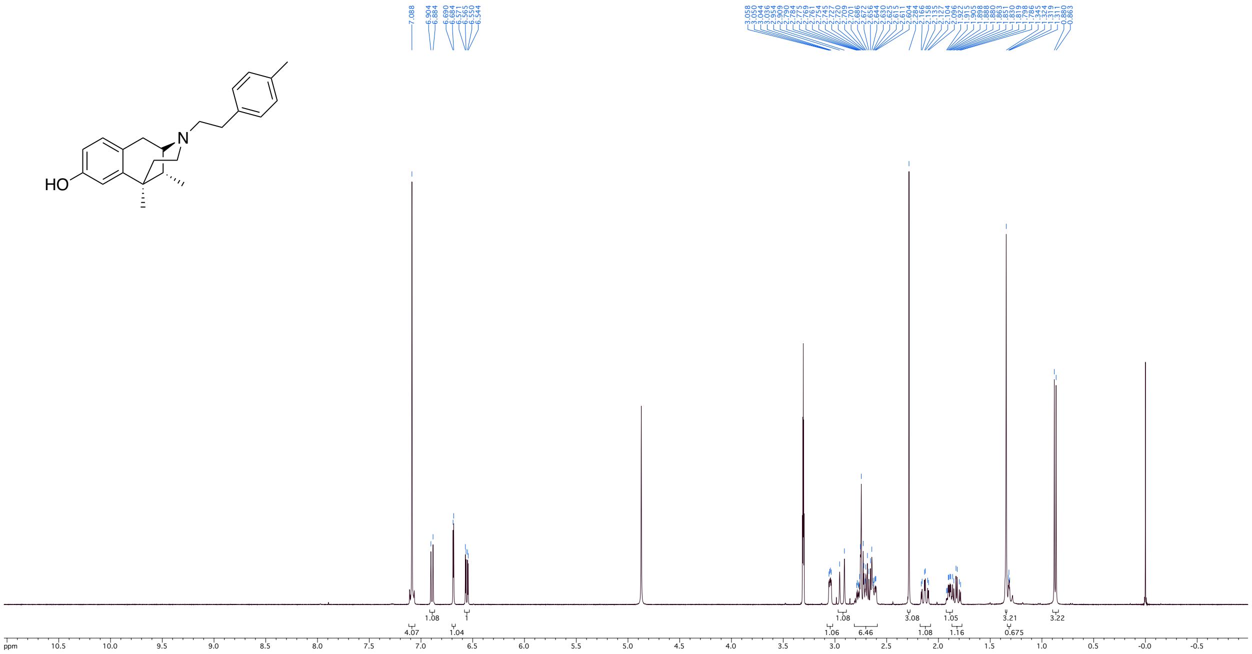

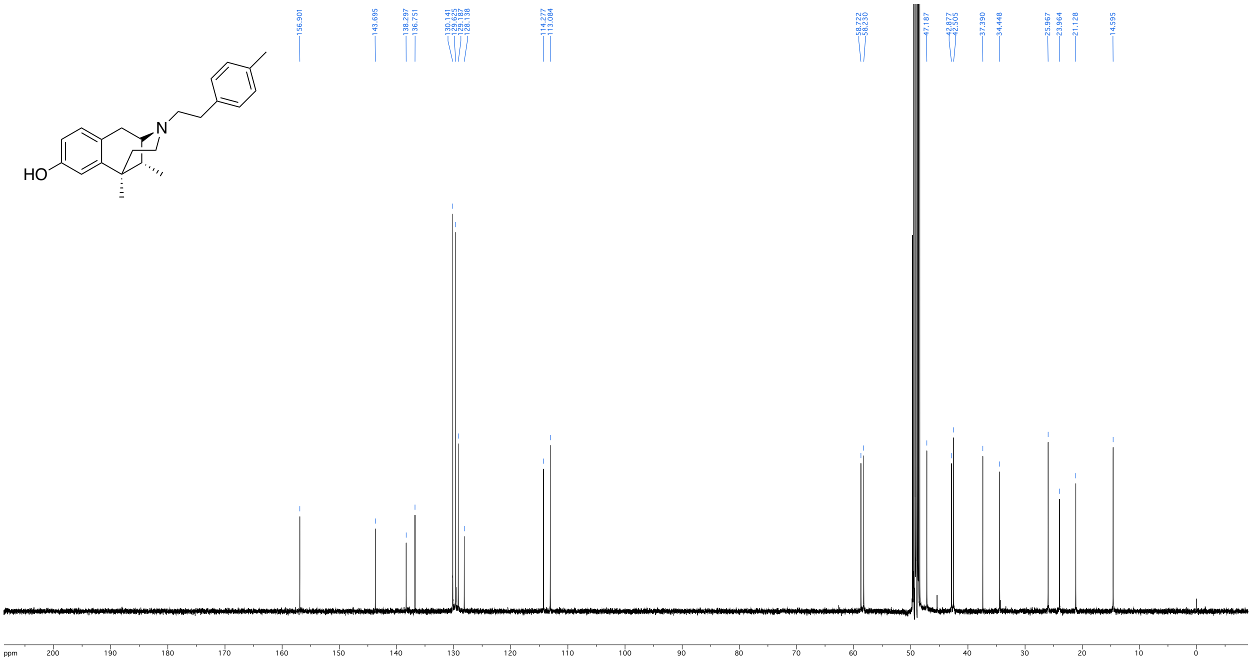

^{1}H NMR and ^{13}C NMR of (–)-20

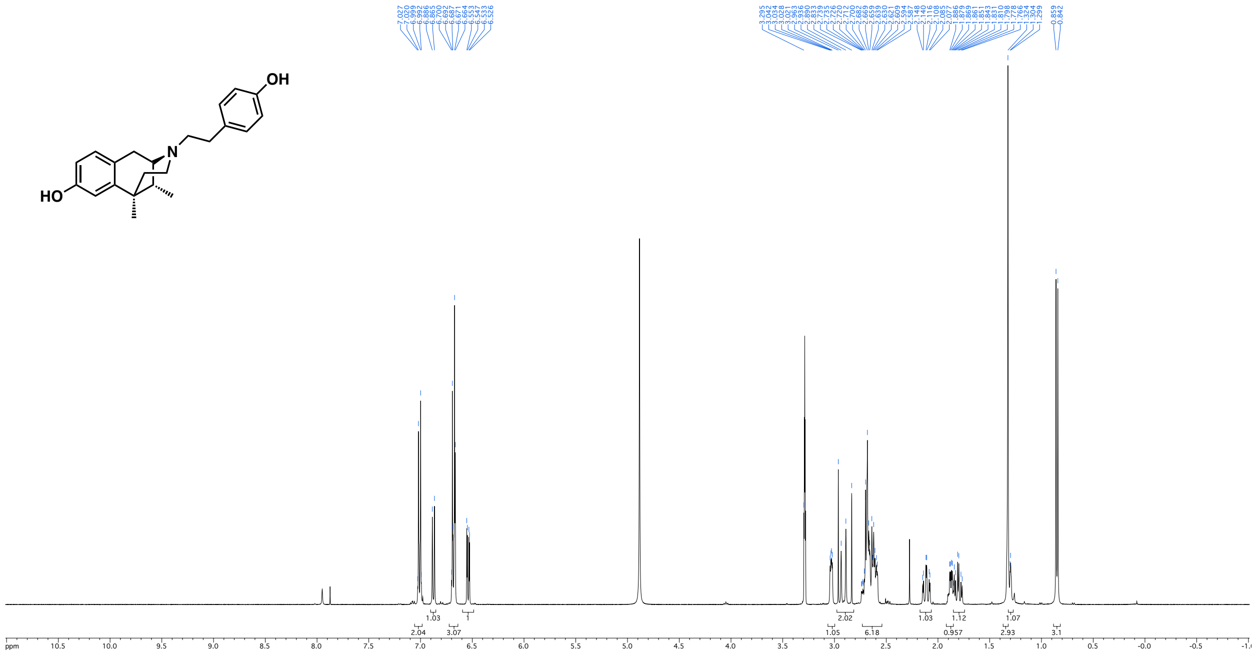

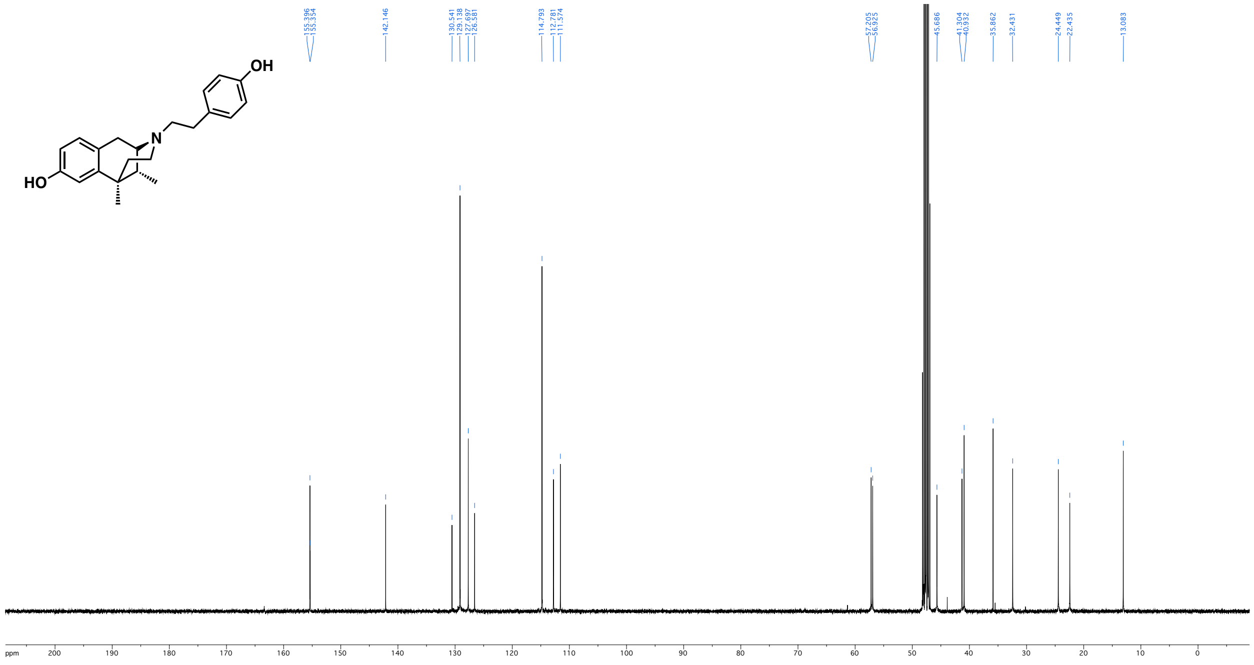

^{1}H NMR and ^{13}C NMR of (–)-21

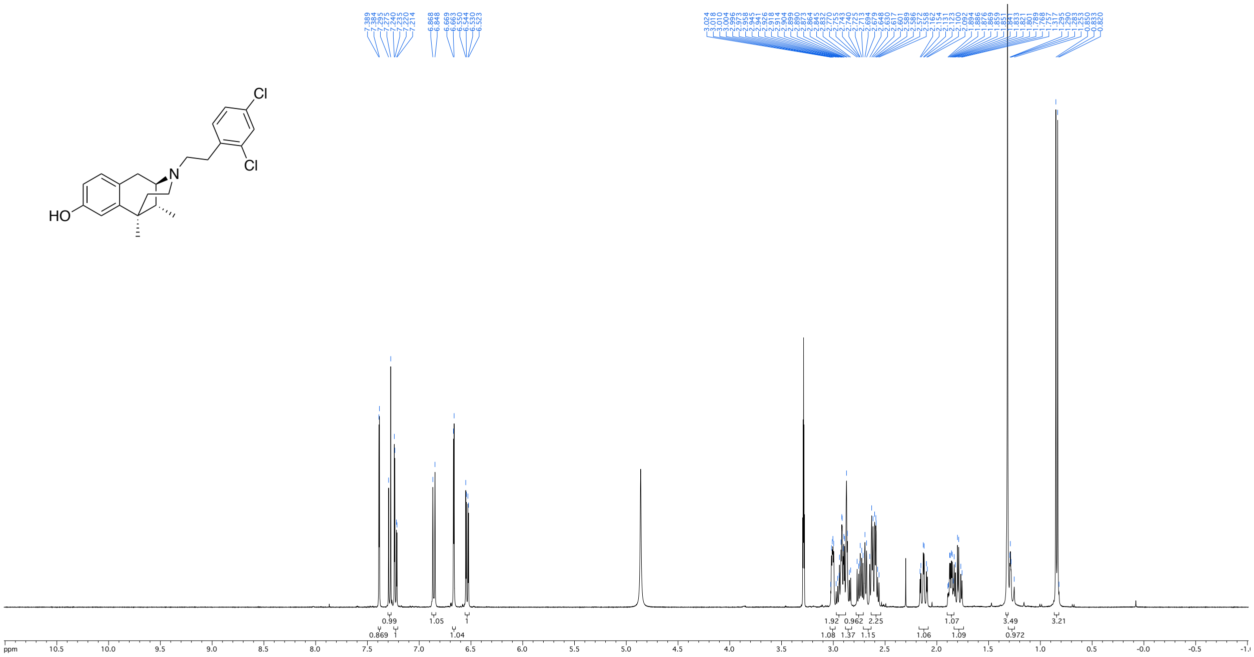

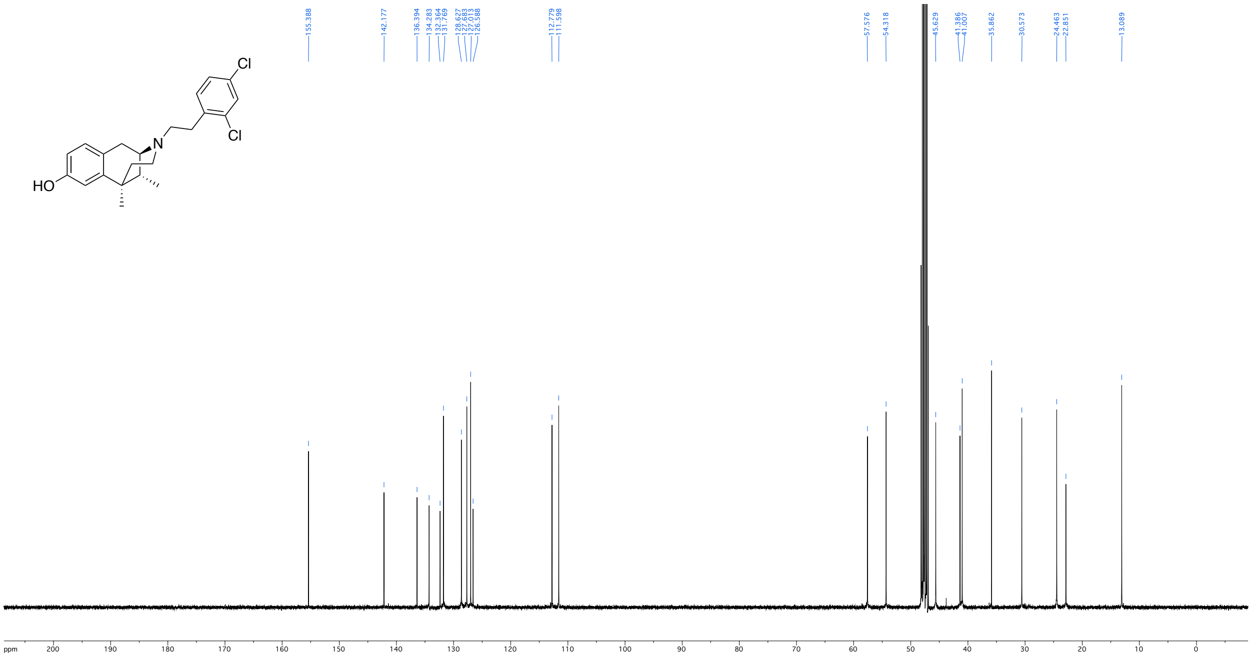

^{1}H NMR and ^{13}C NMR of (–)-22

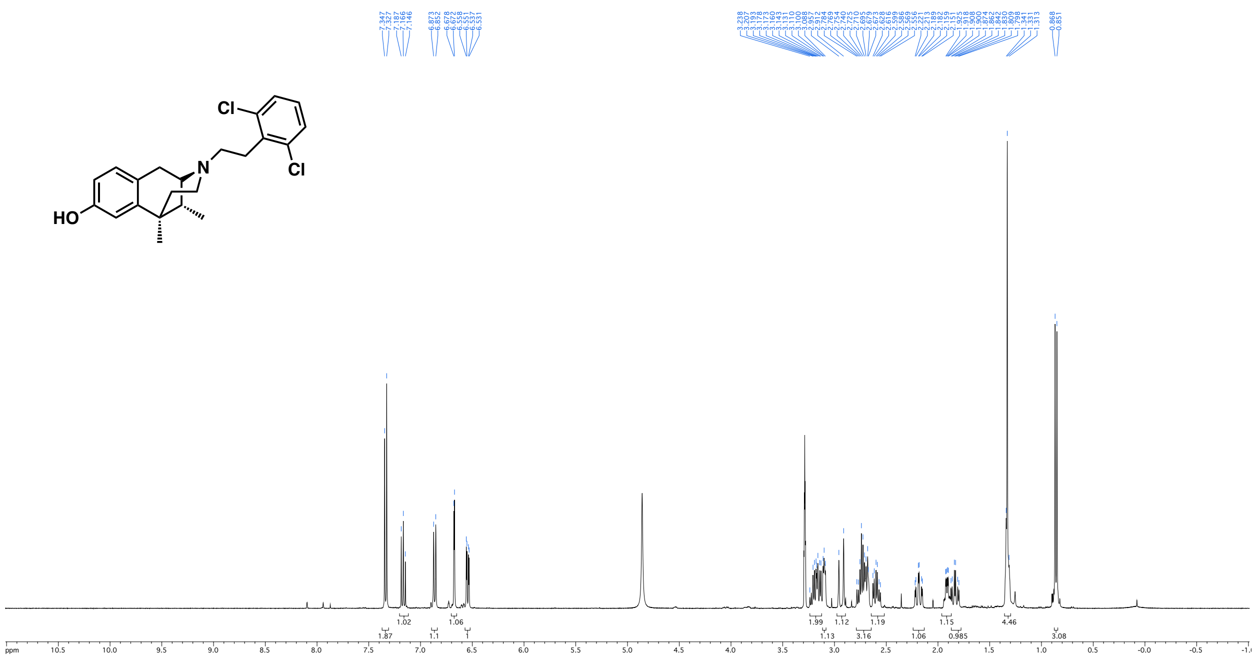

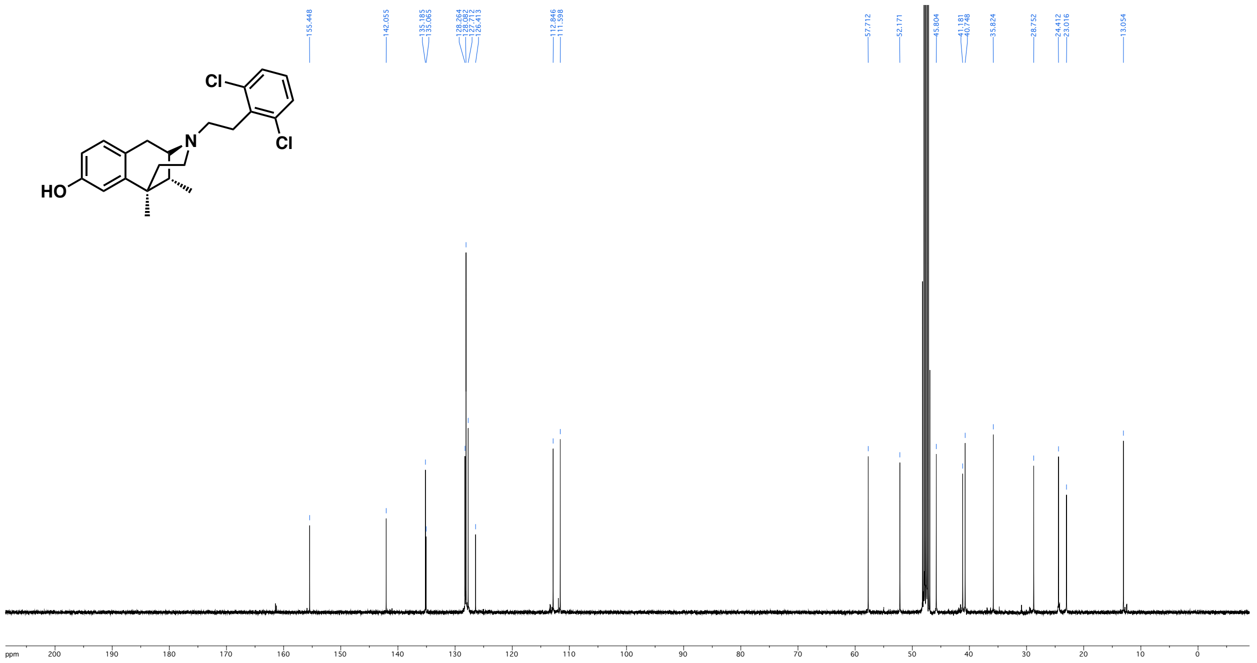

^{1}H NMR and ^{13}C NMR of (–)-23

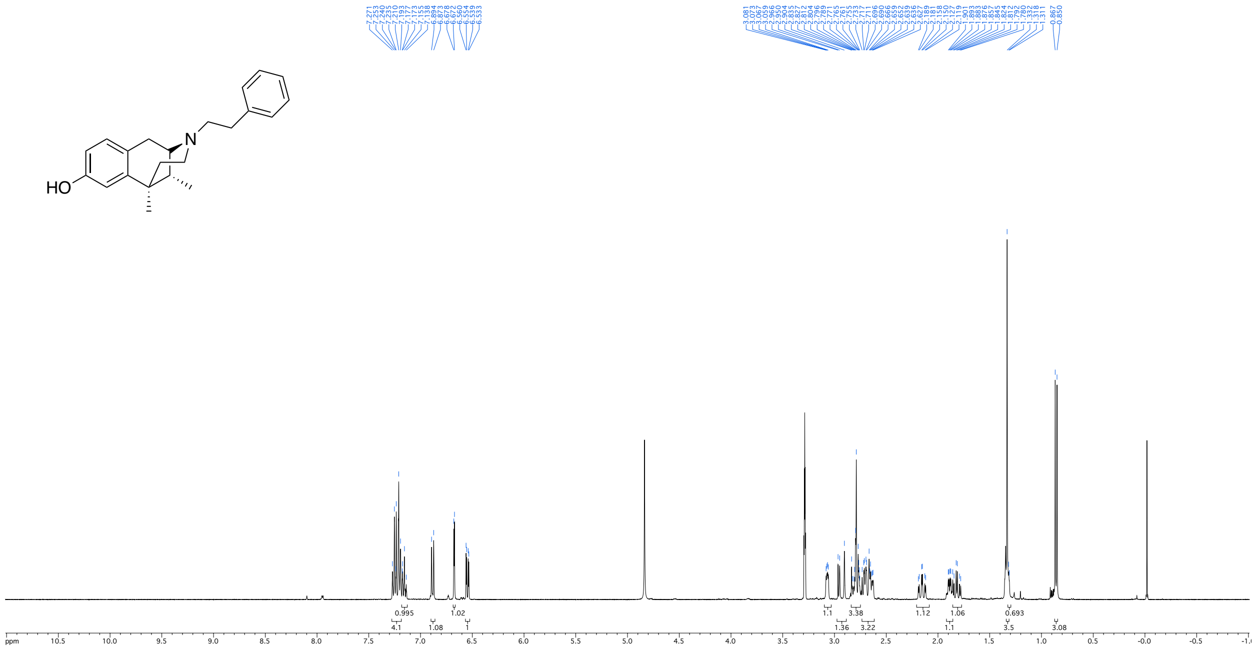

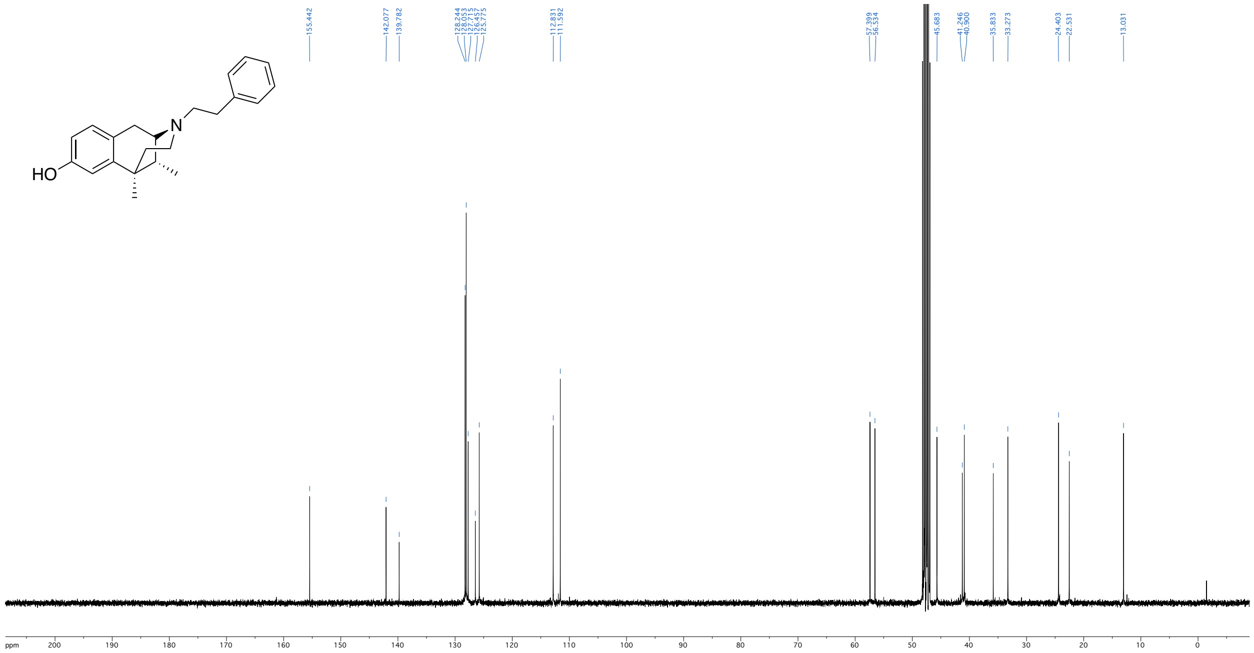

^{1}H NMR and ^{13}C NMR of (–)-24

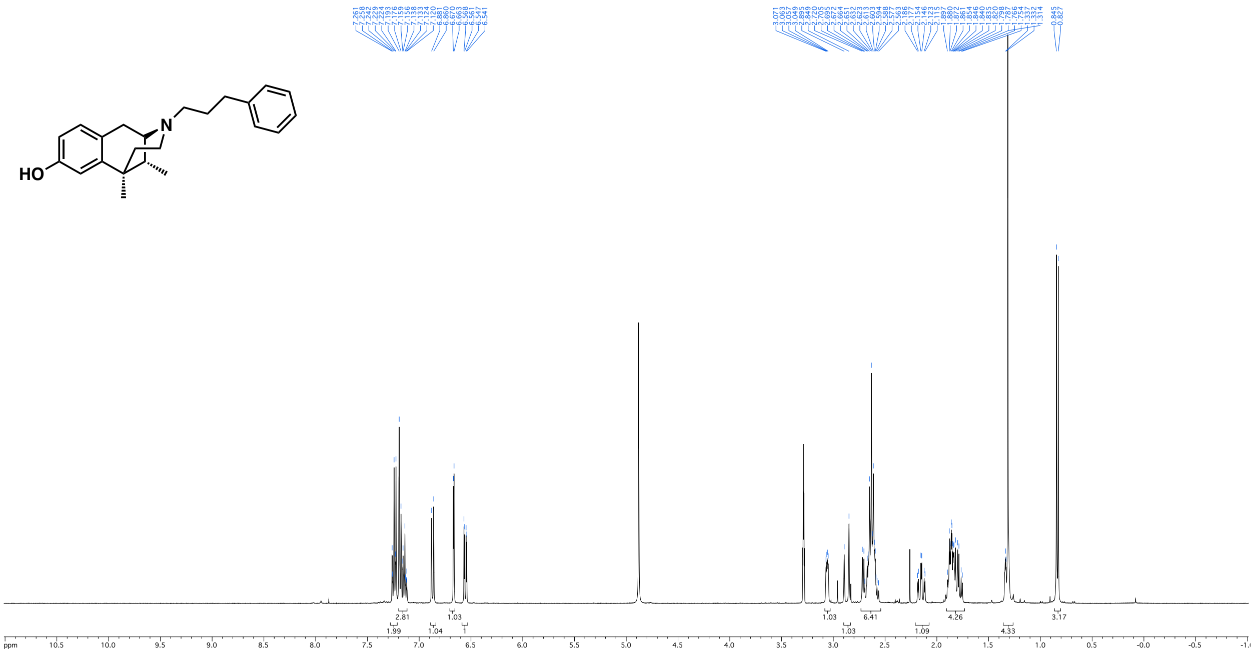

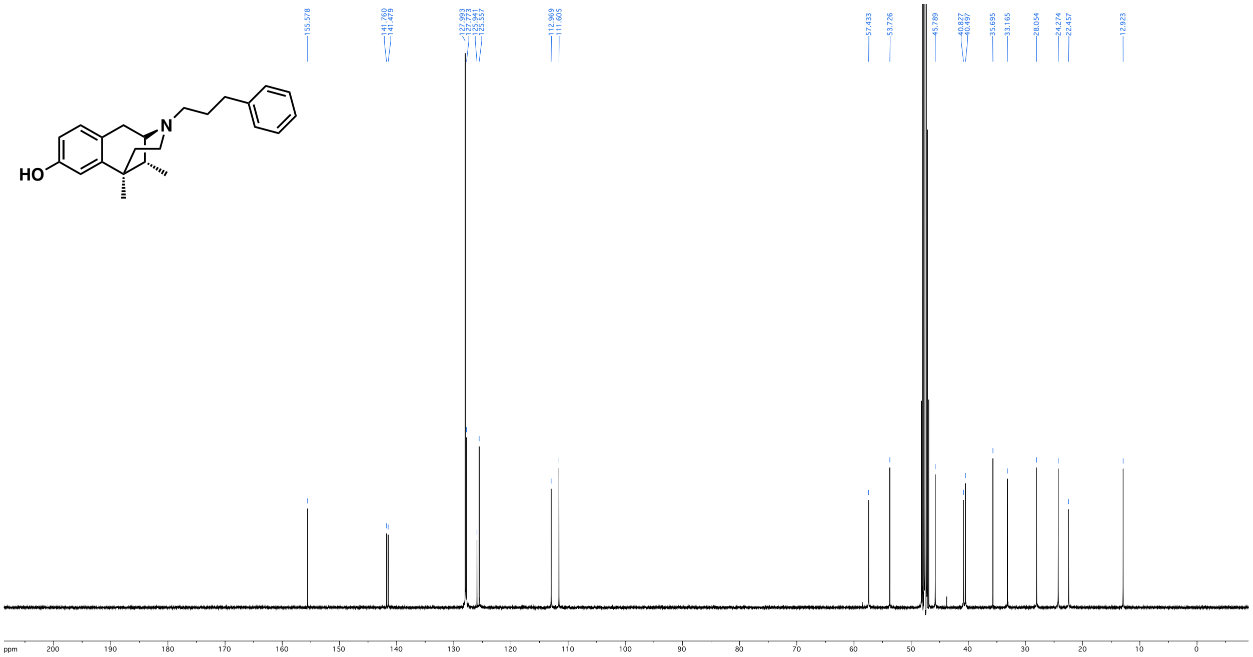

^{1}H NMR and ^{13}C NMR of (–)-25

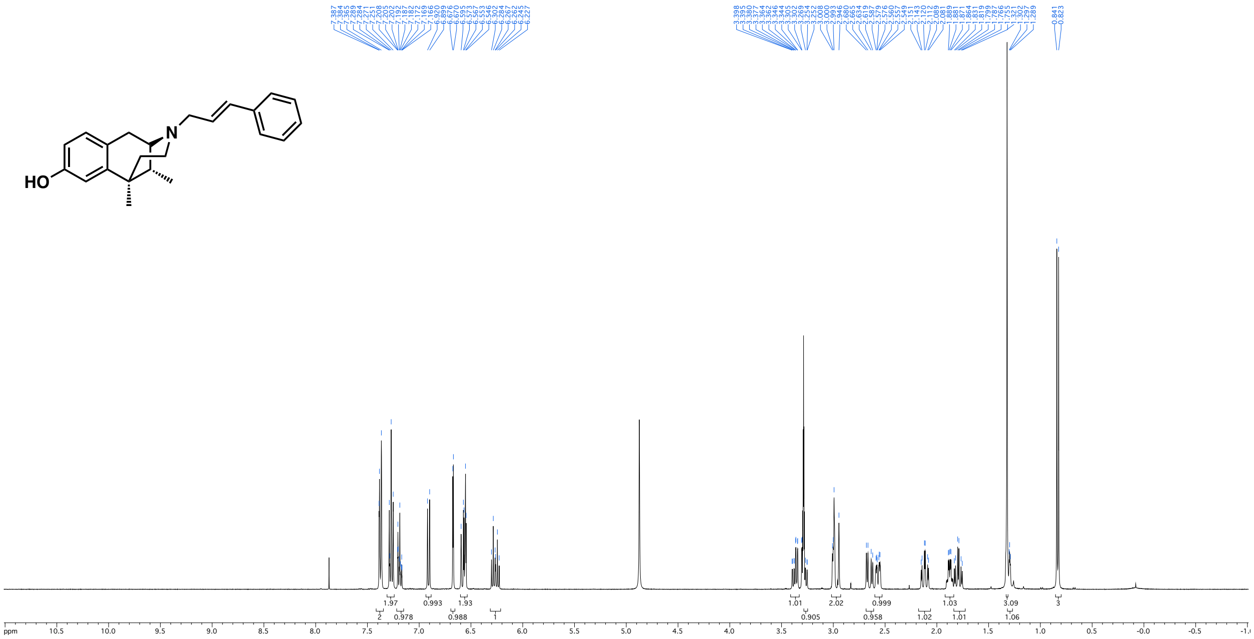

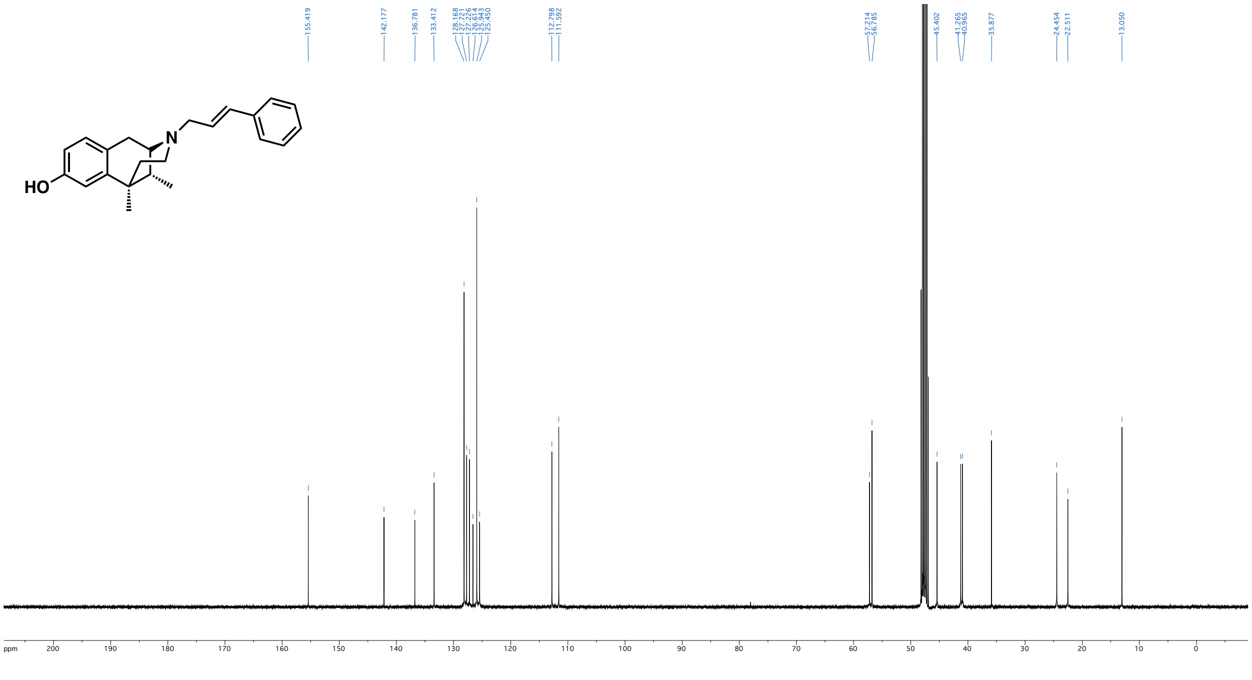

^{1}H NMR and ^{13}C NMR of (–)-26

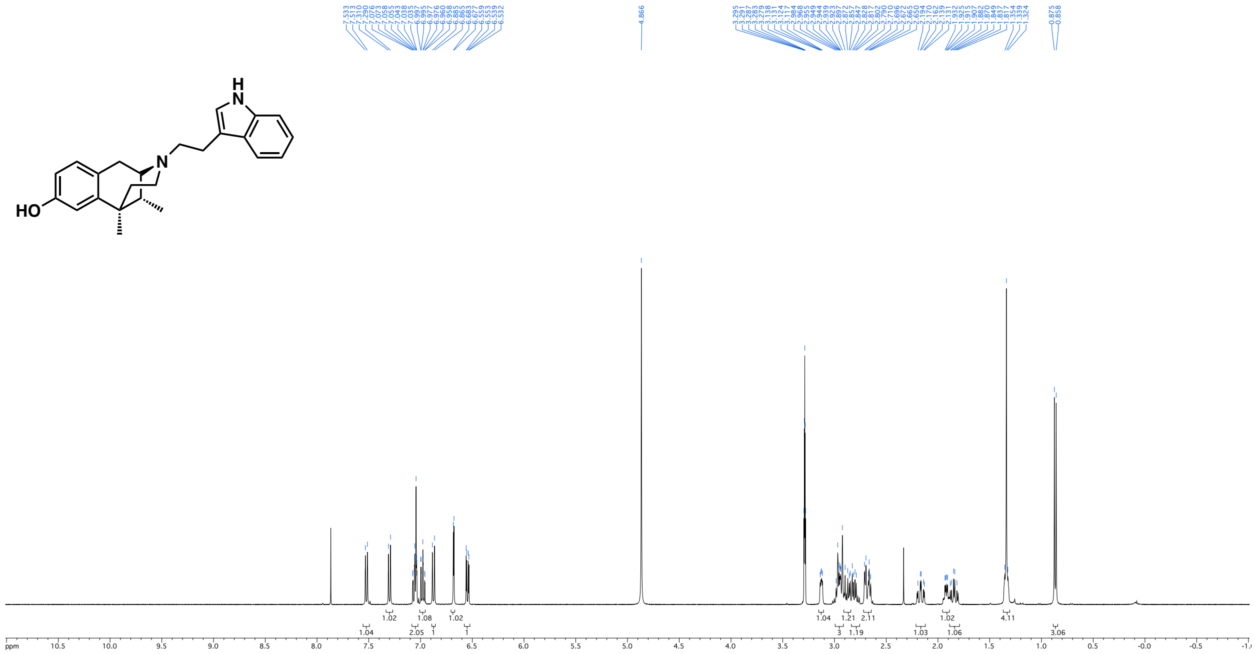

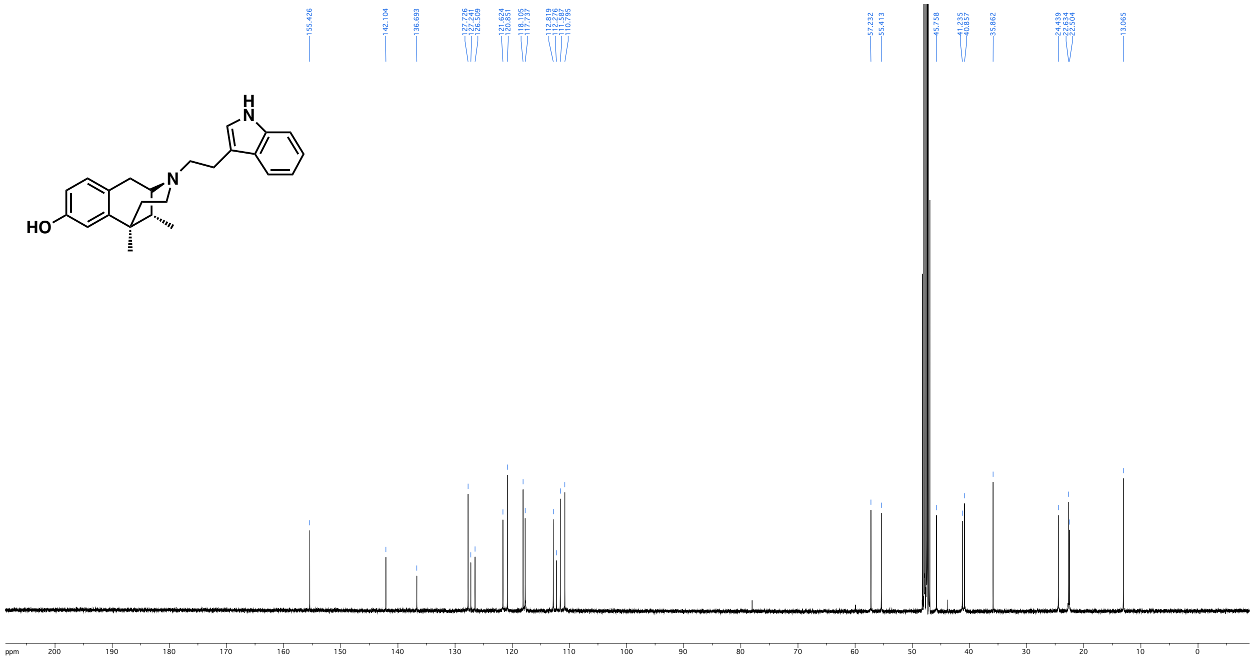

^{1}H NMR and ^{13}C NMR of (–)-27

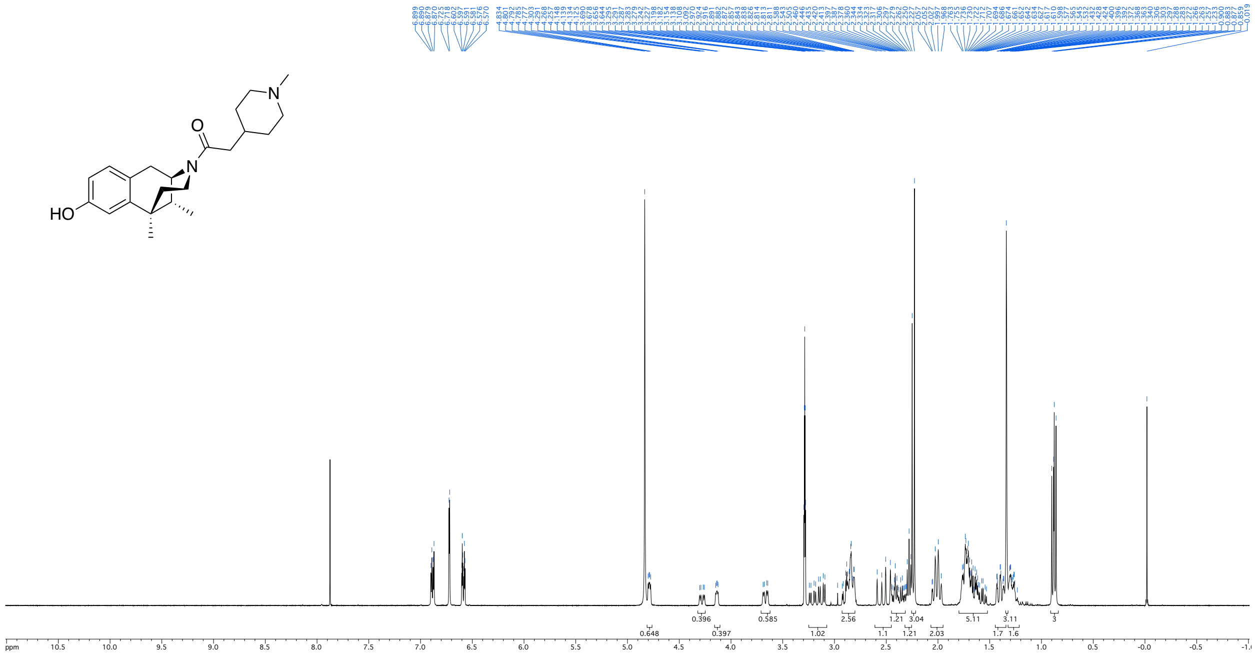

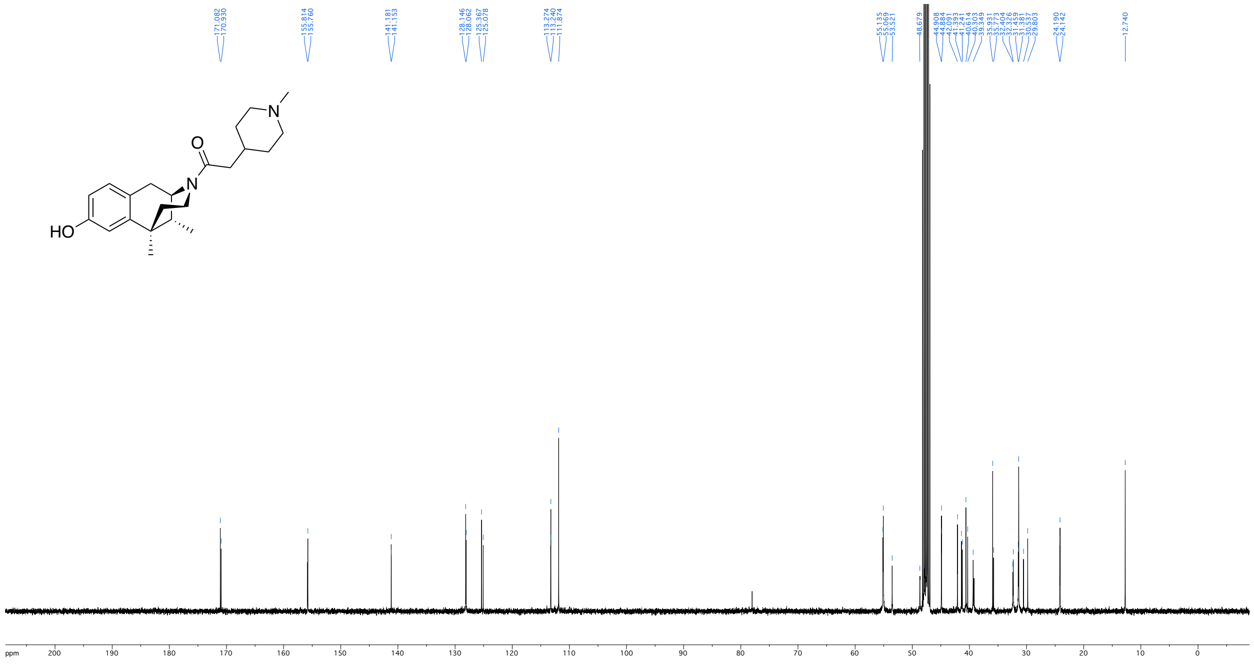

^{1}H NMR and ^{13}C NMR of (–)-28

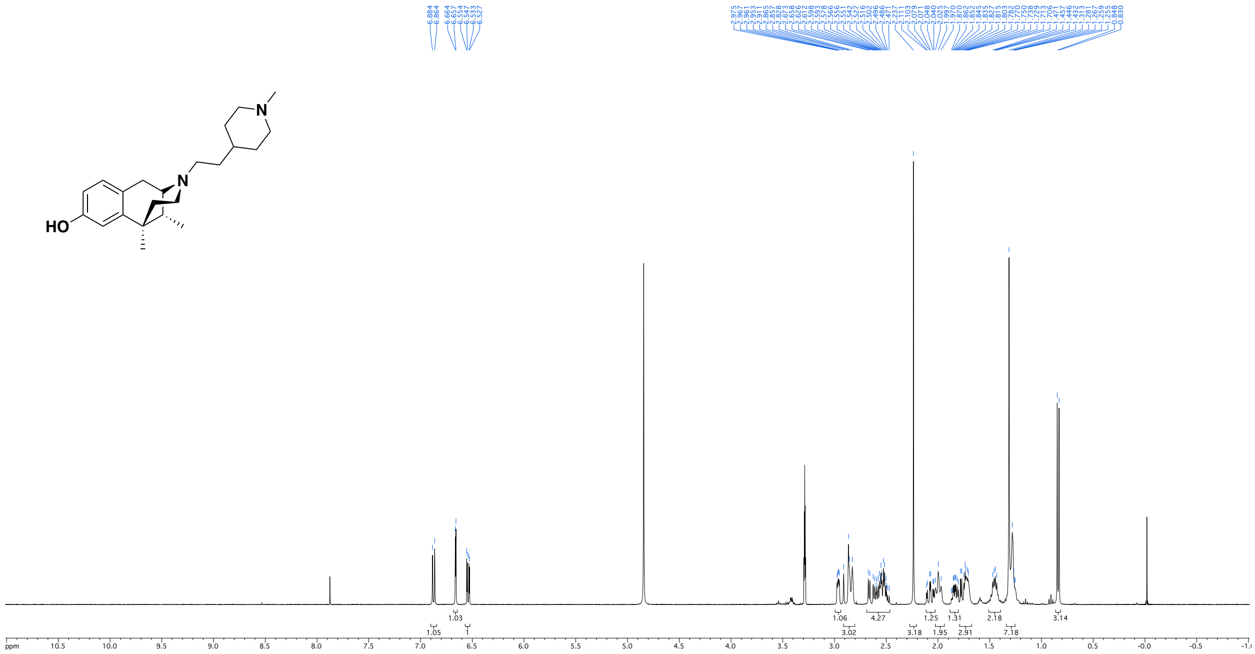

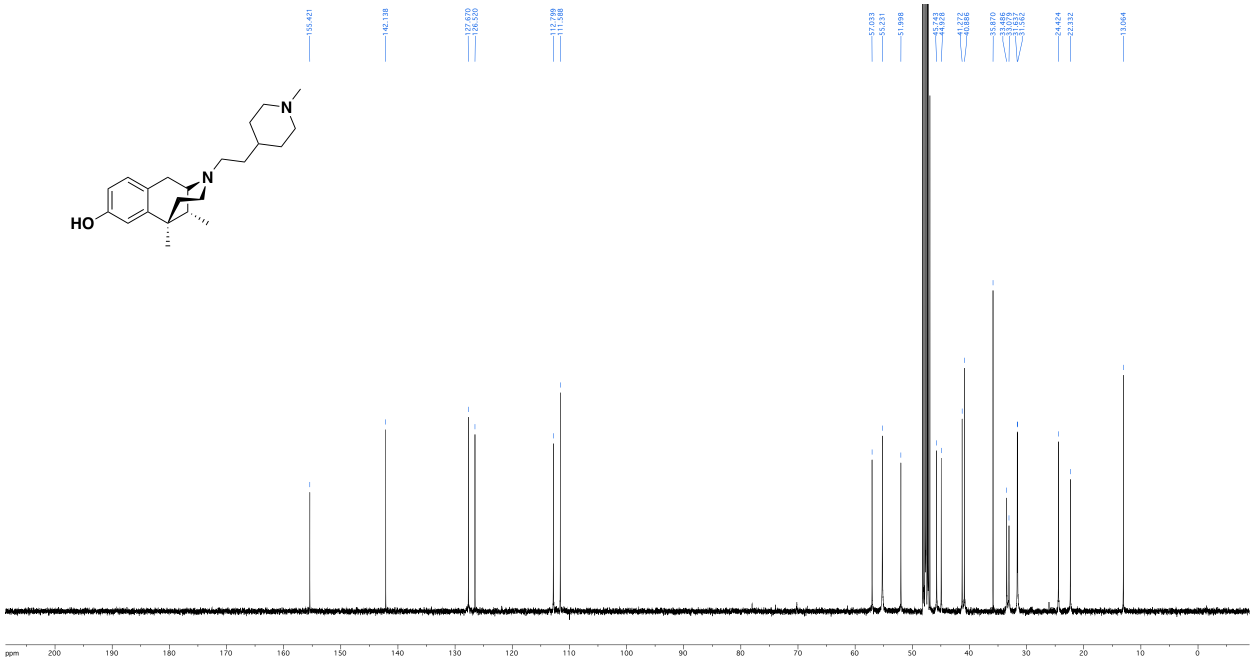

^{1}H NMR and ^{13}C NMR of 30

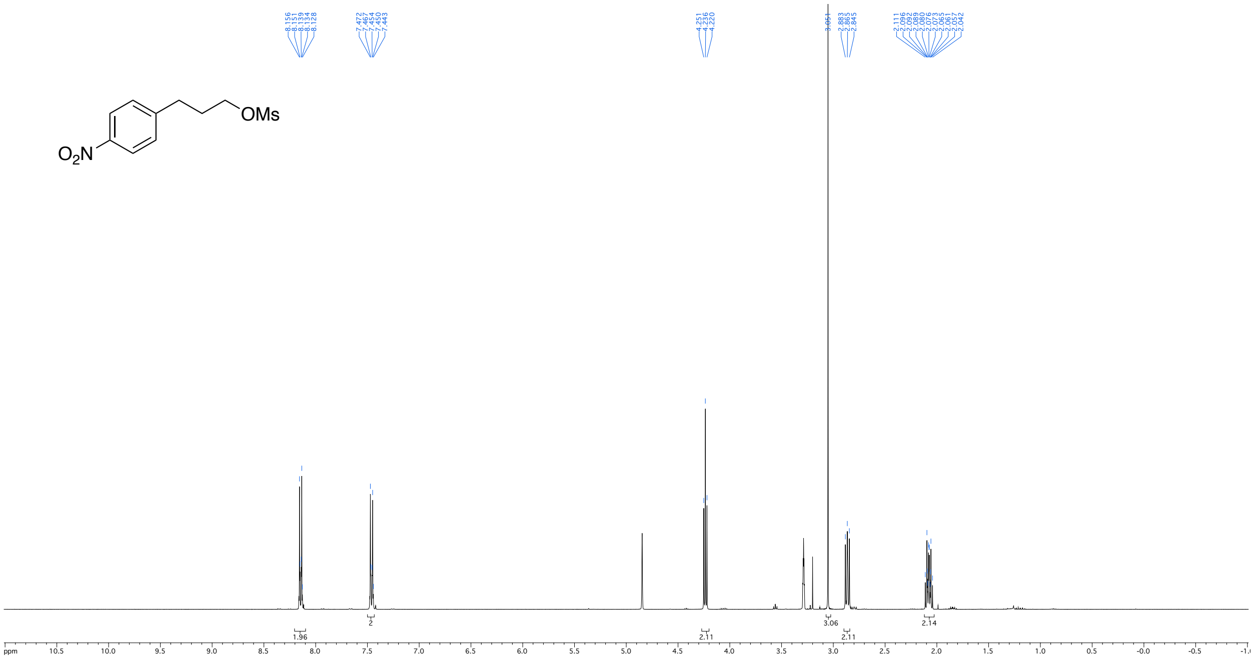

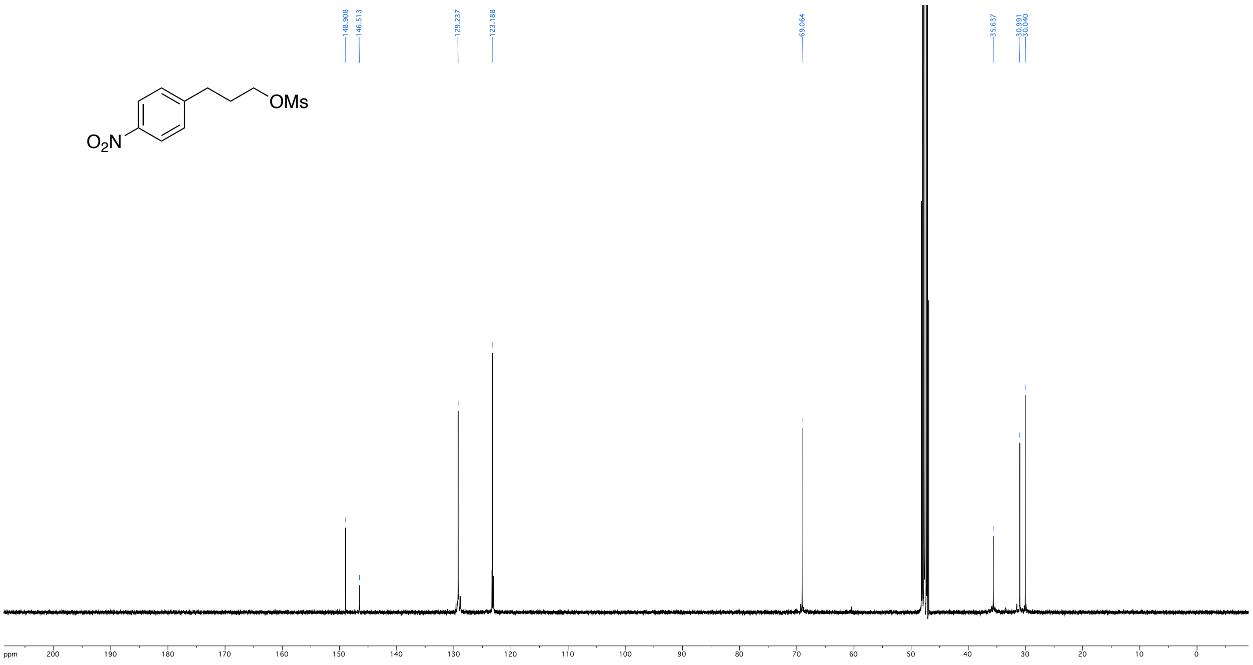

^{1}H NMR and ^{13}C NMR of (–)-31

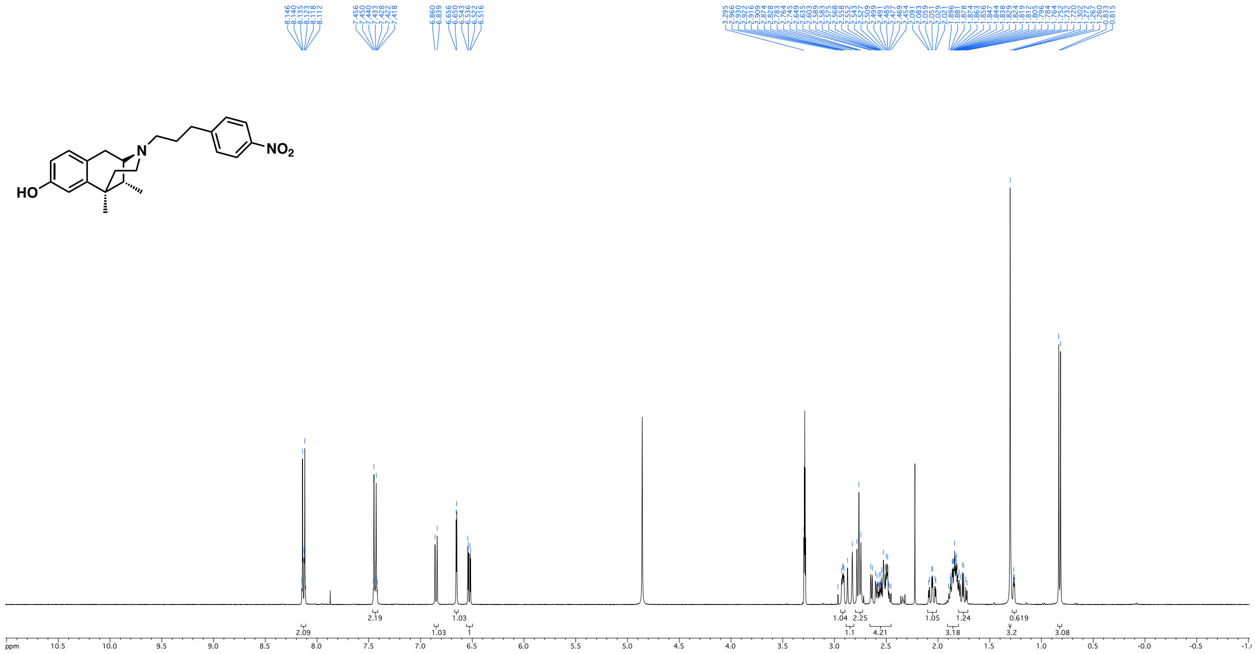

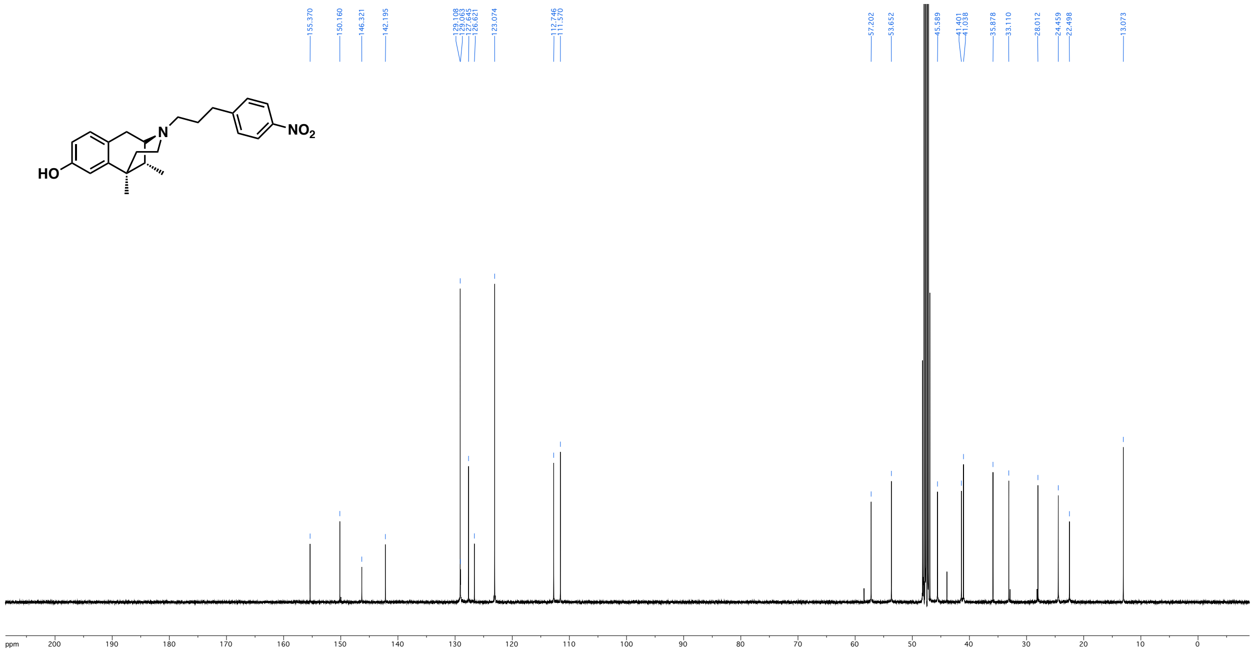

^{1}H NMR and ^{13}C NMR of 32

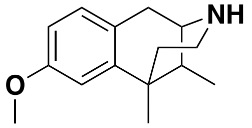

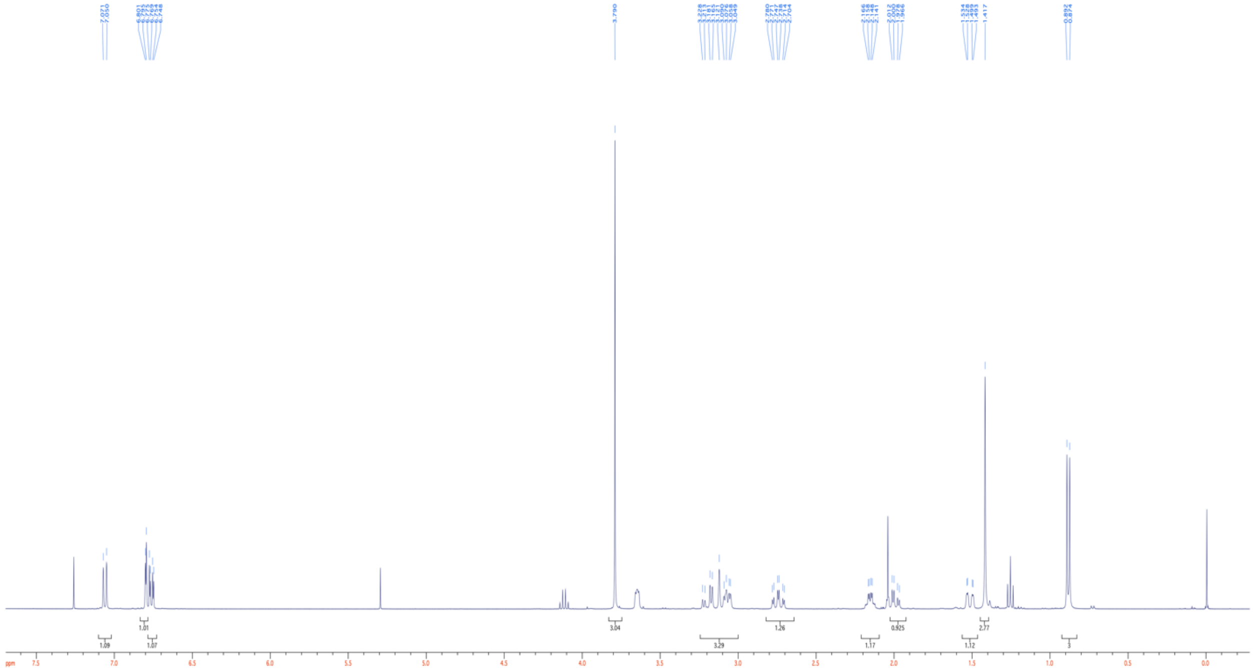

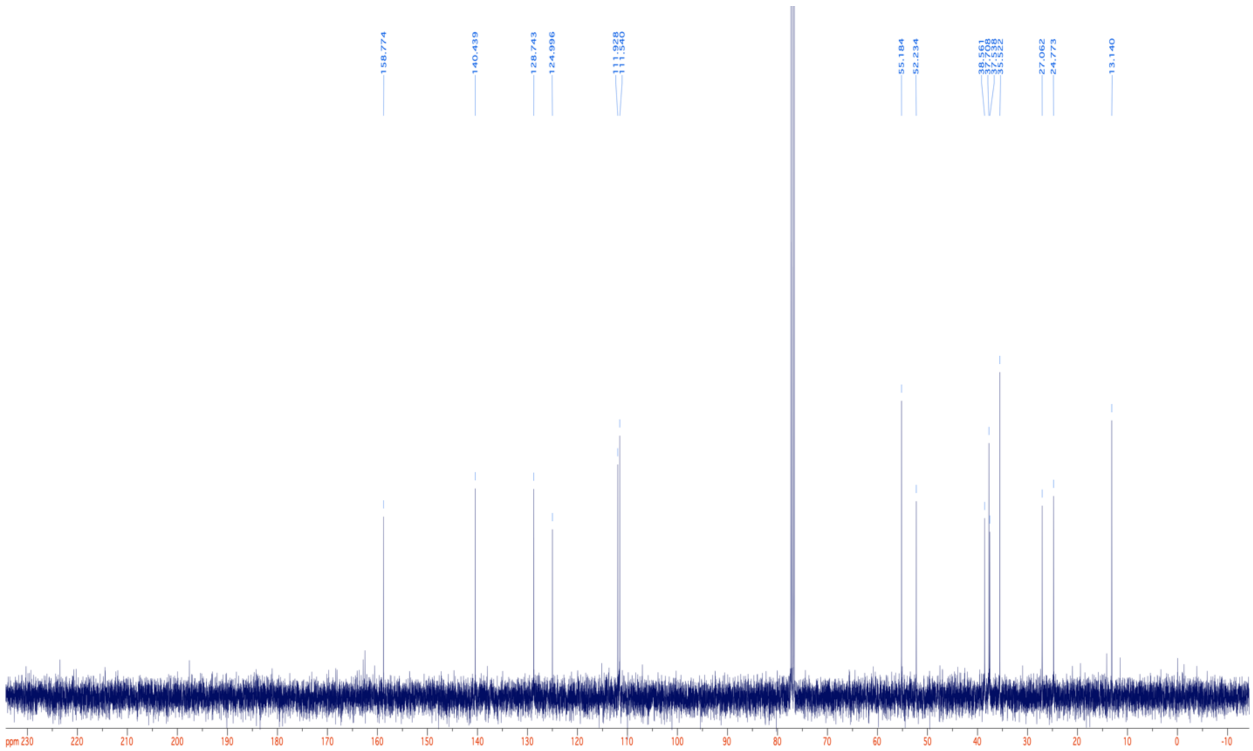

^{1}H NMR and ^{13}C NMR of 33

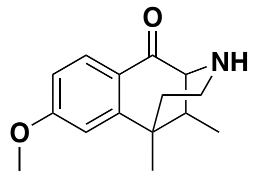

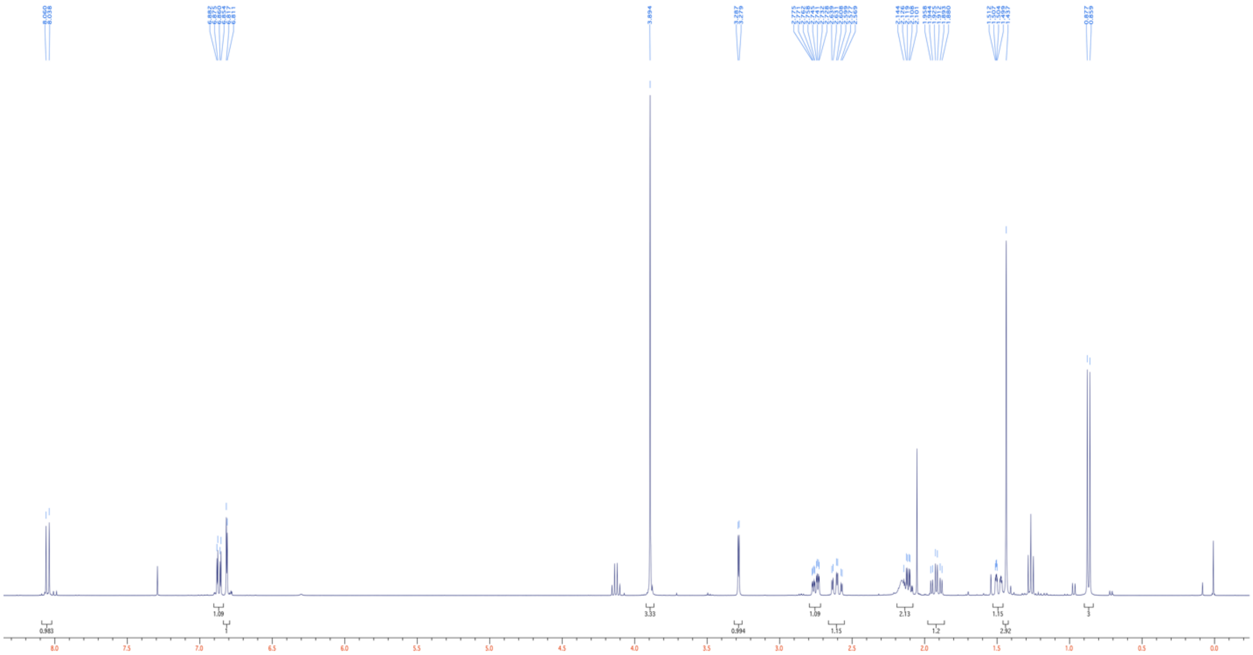

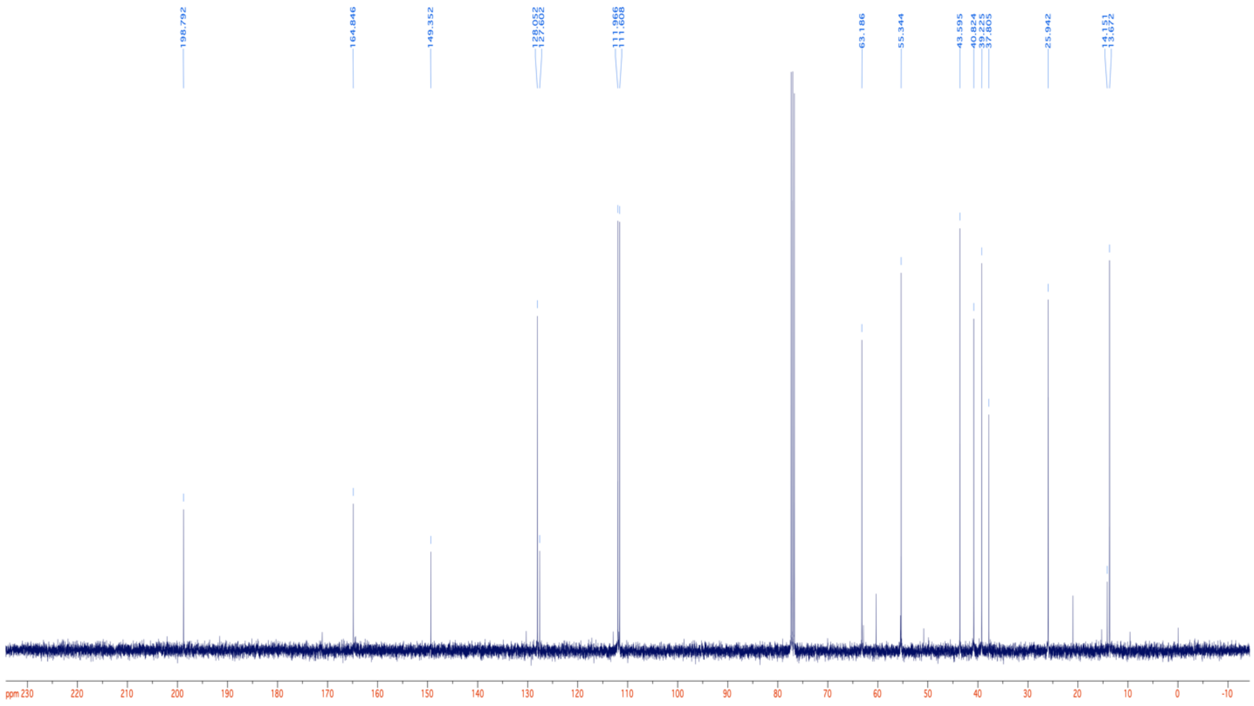

^{1}H NMR and ^{13}C NMR of *rac*-34

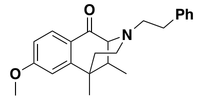

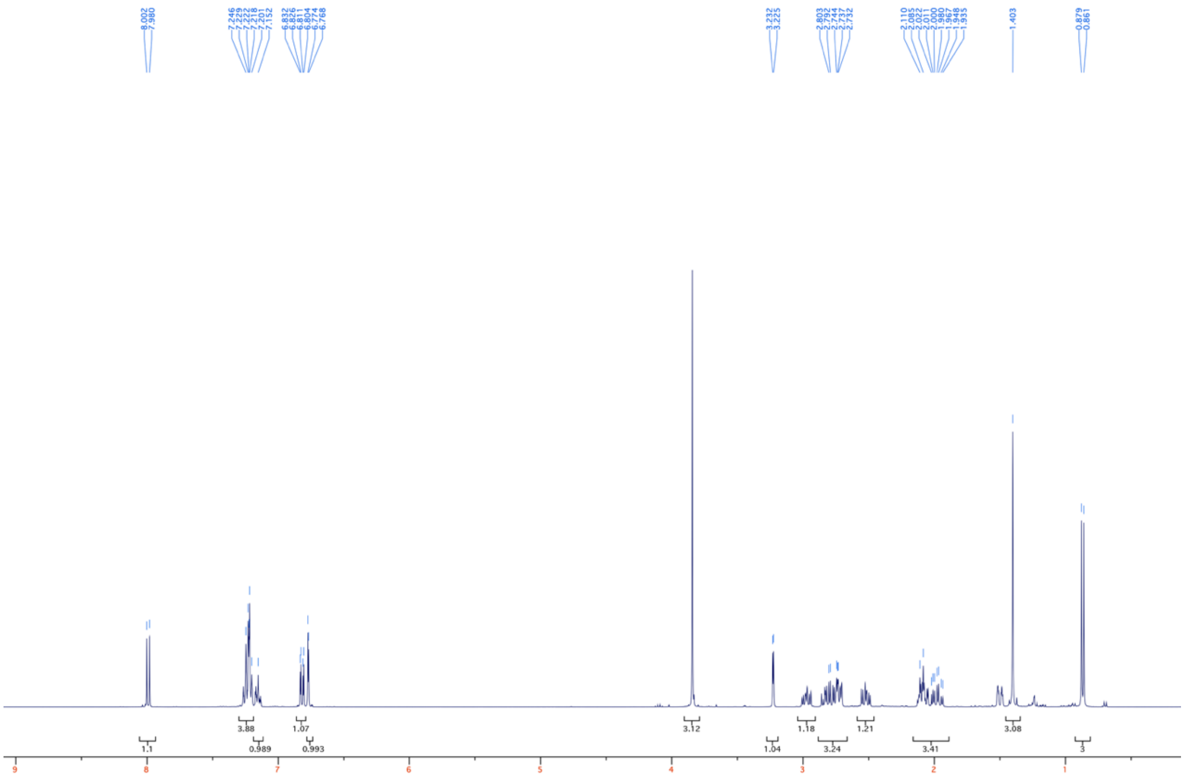

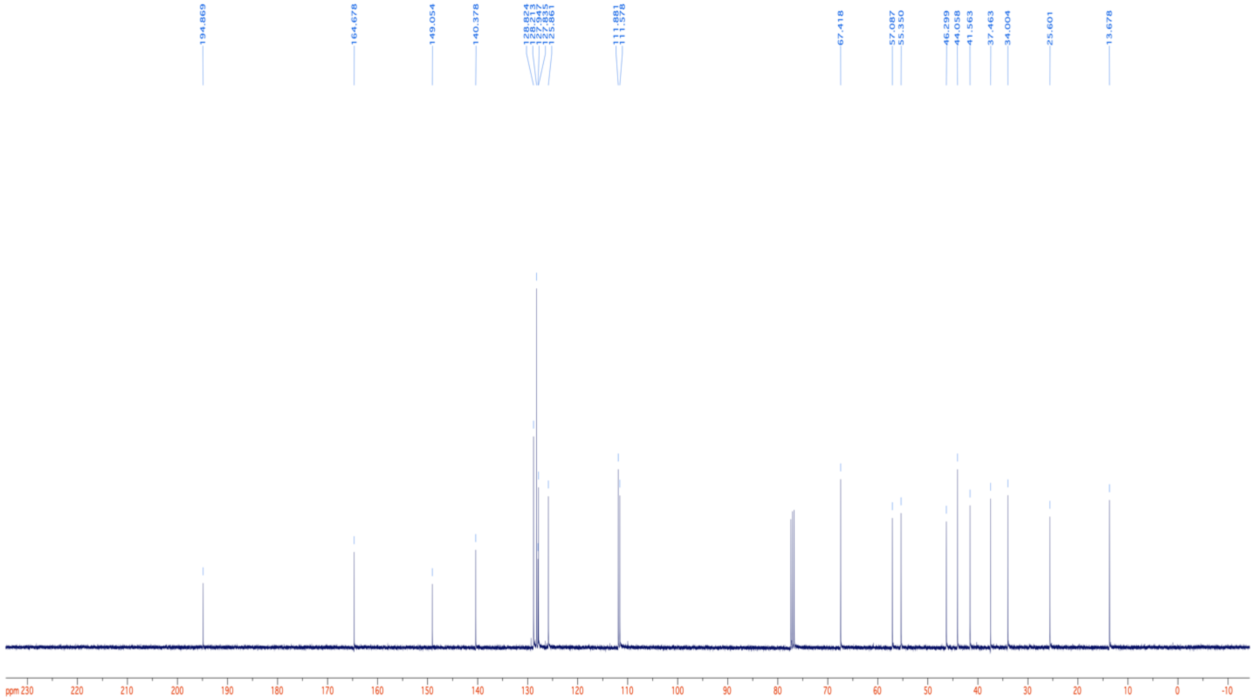

Optical resolution of *rac*-34

Using **1** as resolving agent

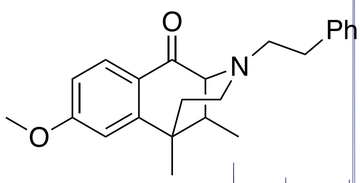

Racemic

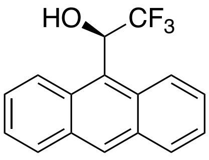

1

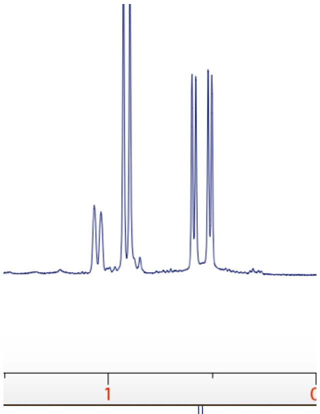

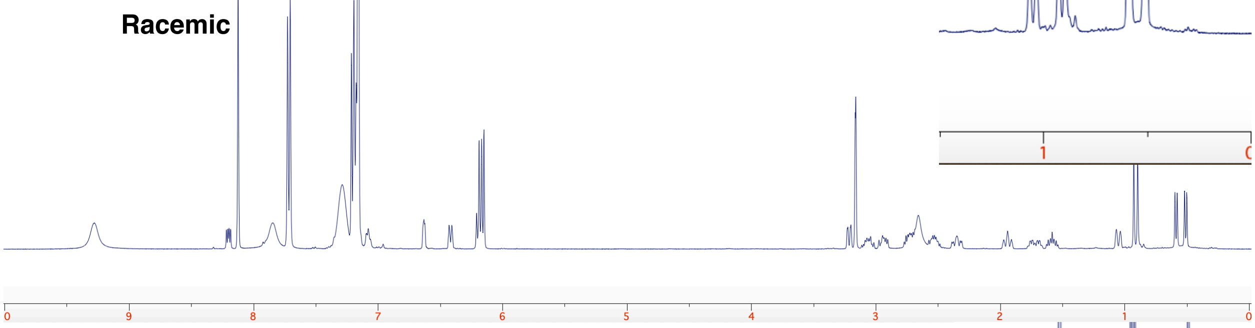

(–)

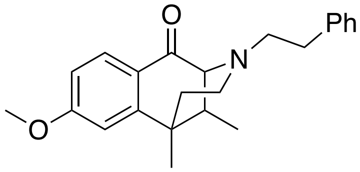

(-) -41.3

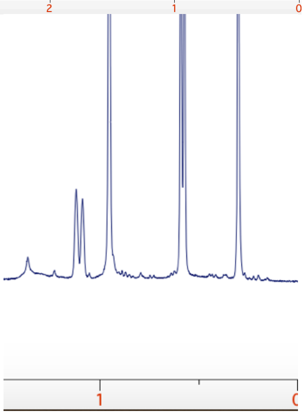

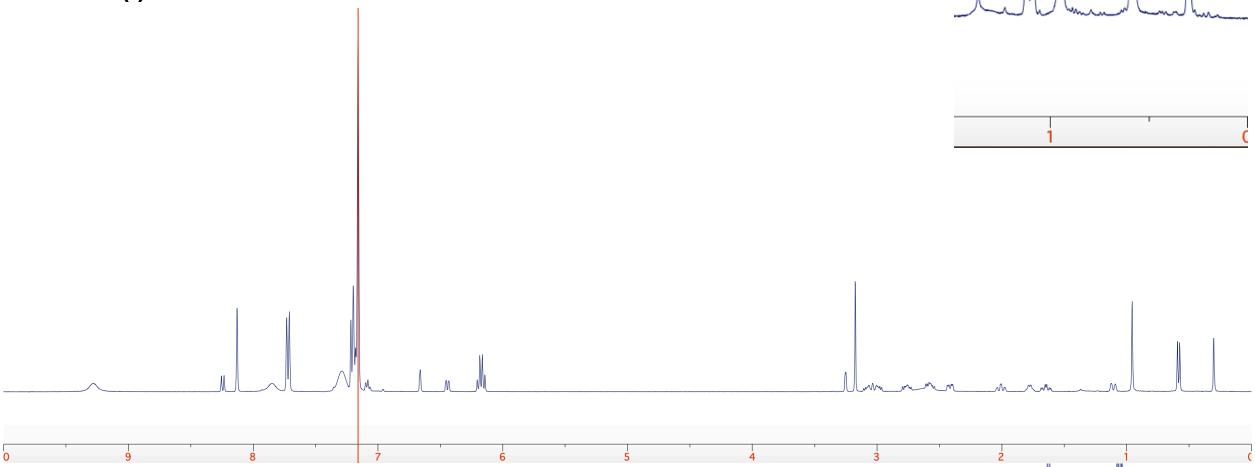

(+)

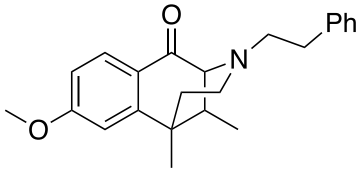

(+) +44.4

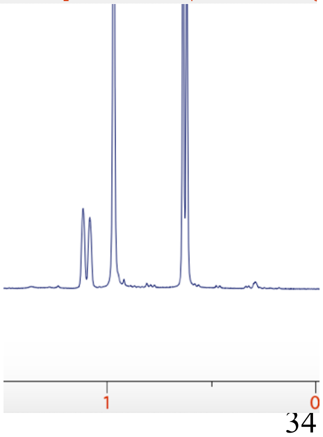

^{1}H NMR and ^{13}C NMR of (–)-34

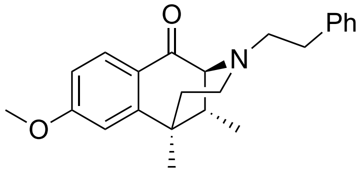

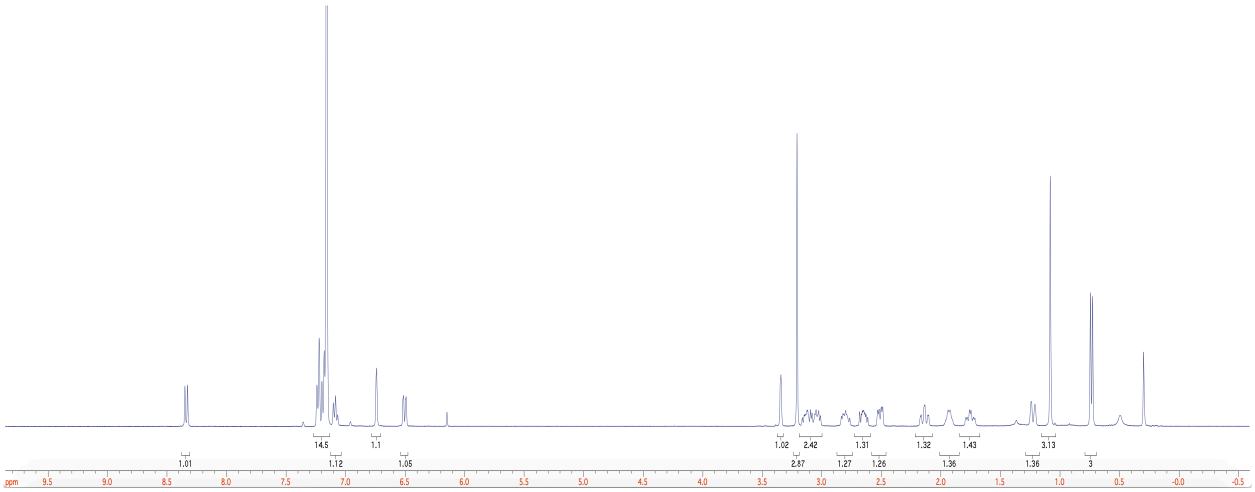

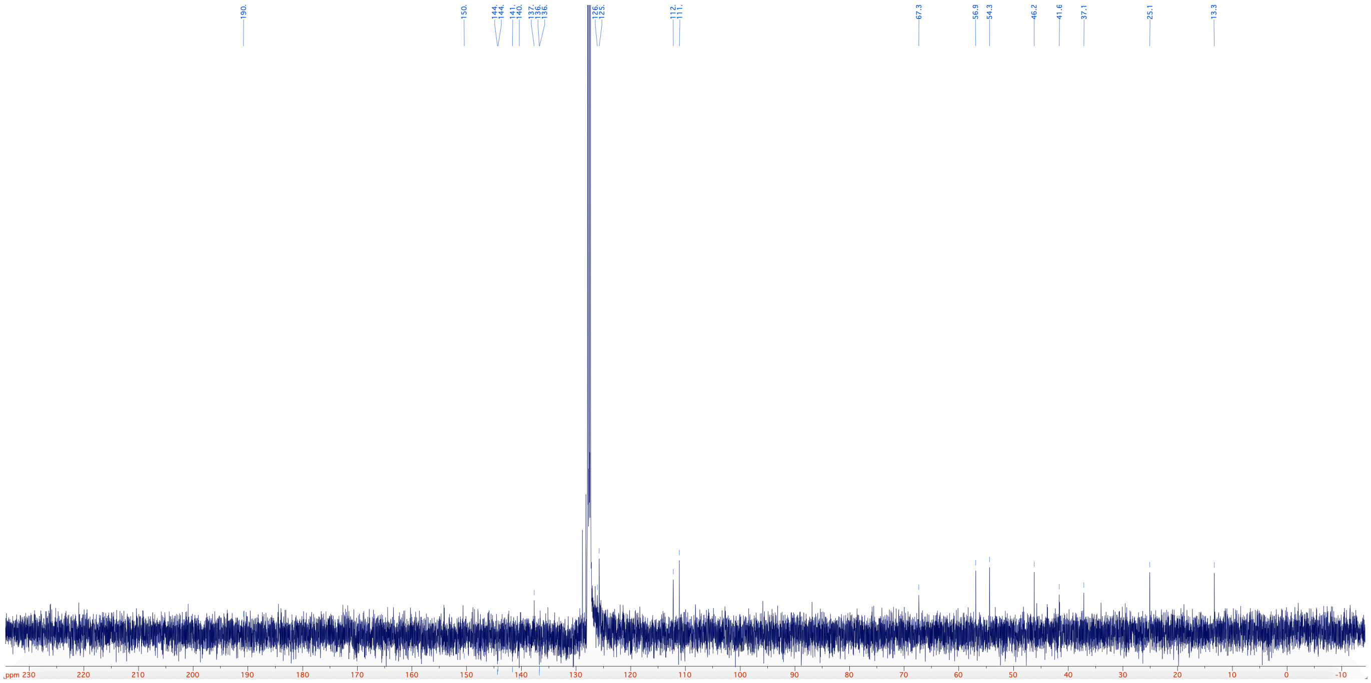

^{1}H NMR and ^{13}C NMR of (+)-34

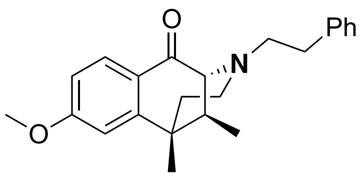

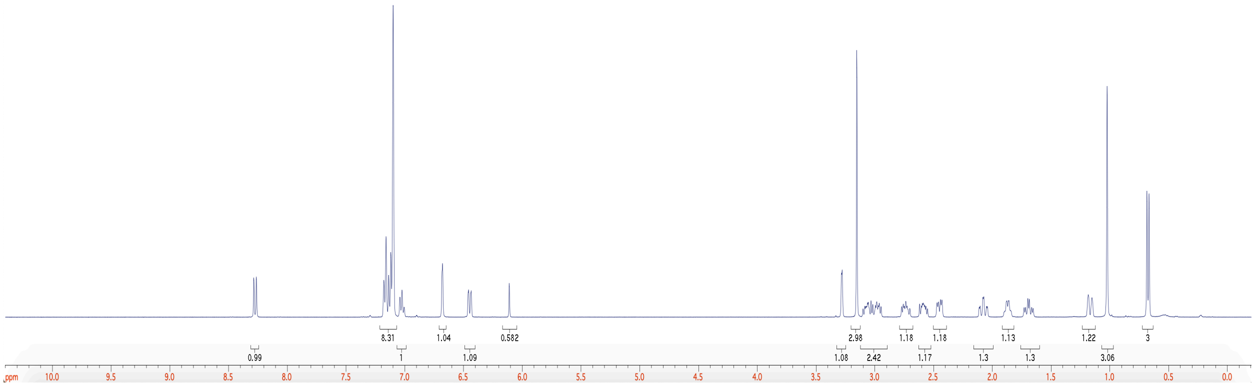

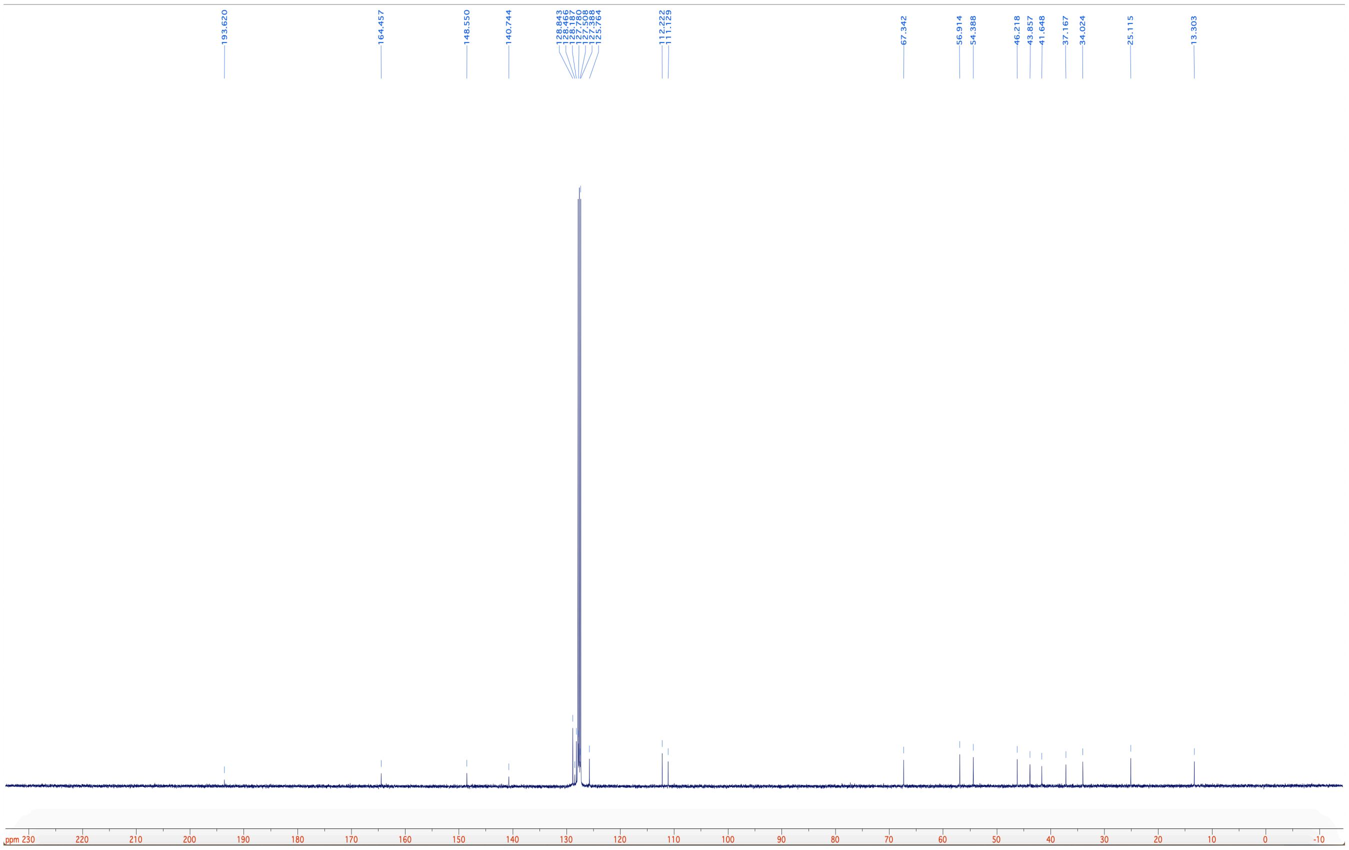

^{1}H NMR and ^{13}C NMR of (–)-37

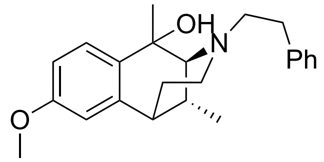

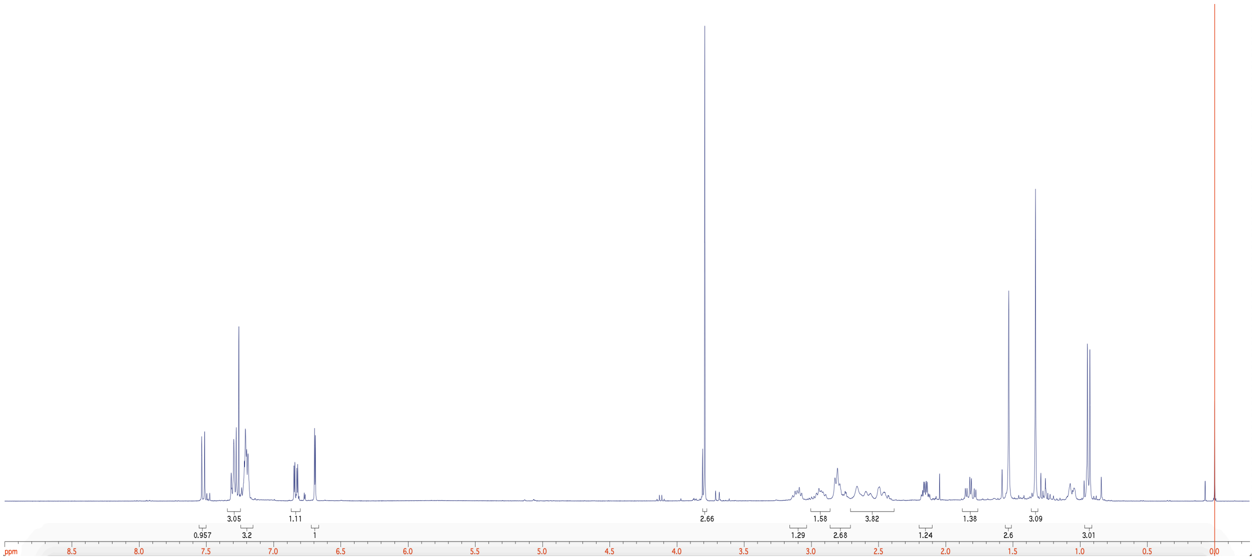

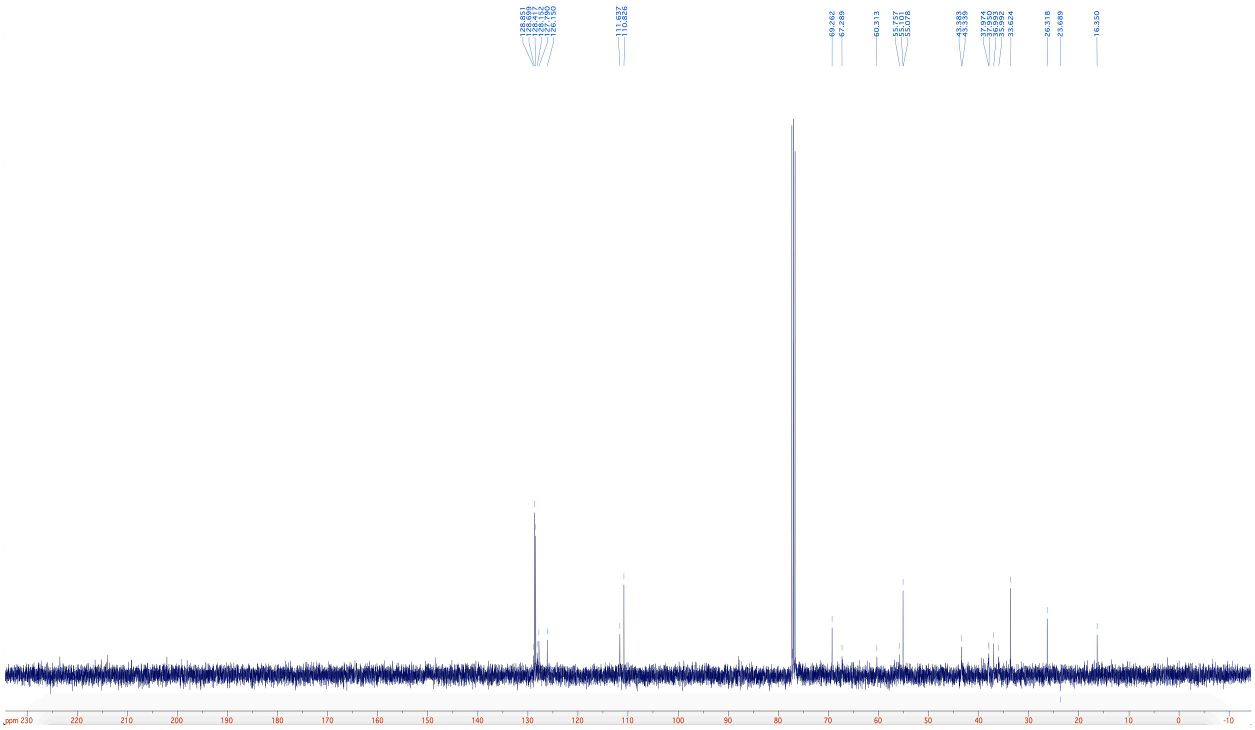

^{1}H NMR and ^{13}C NMR of (+)-37

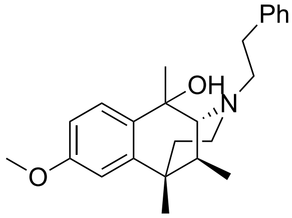

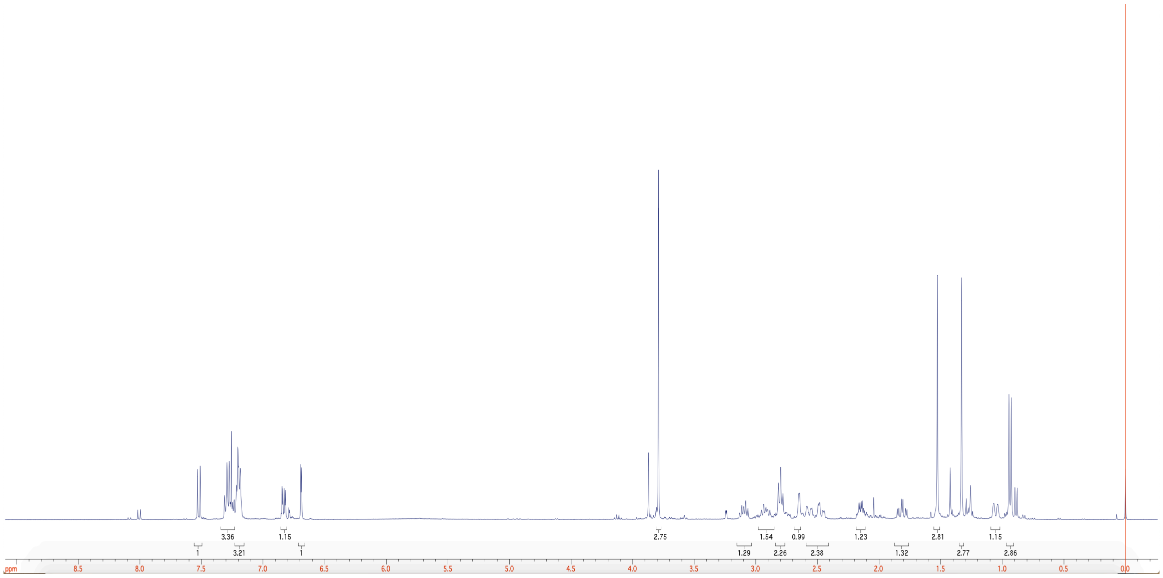

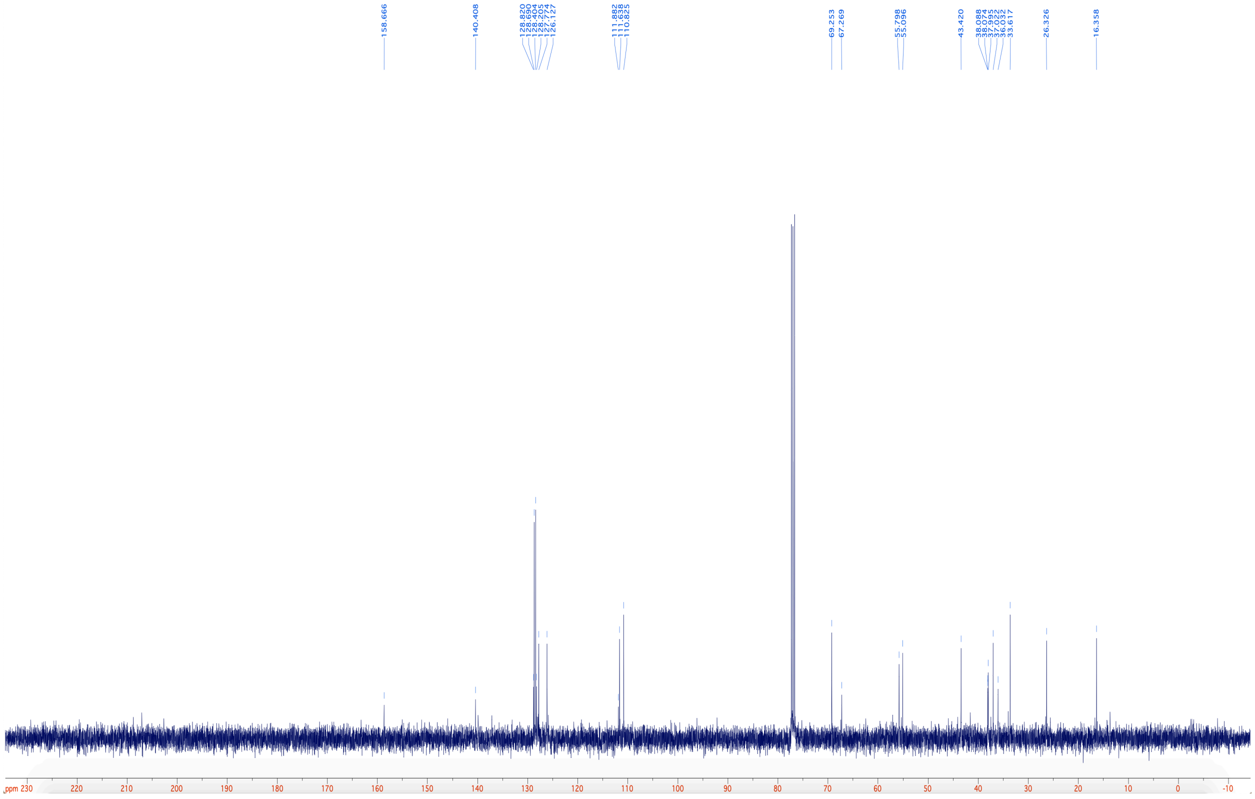

^{1}H NMR and ^{13}C NMR of (–)-38

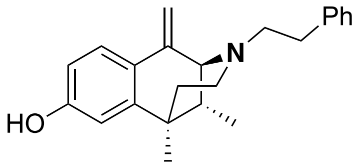

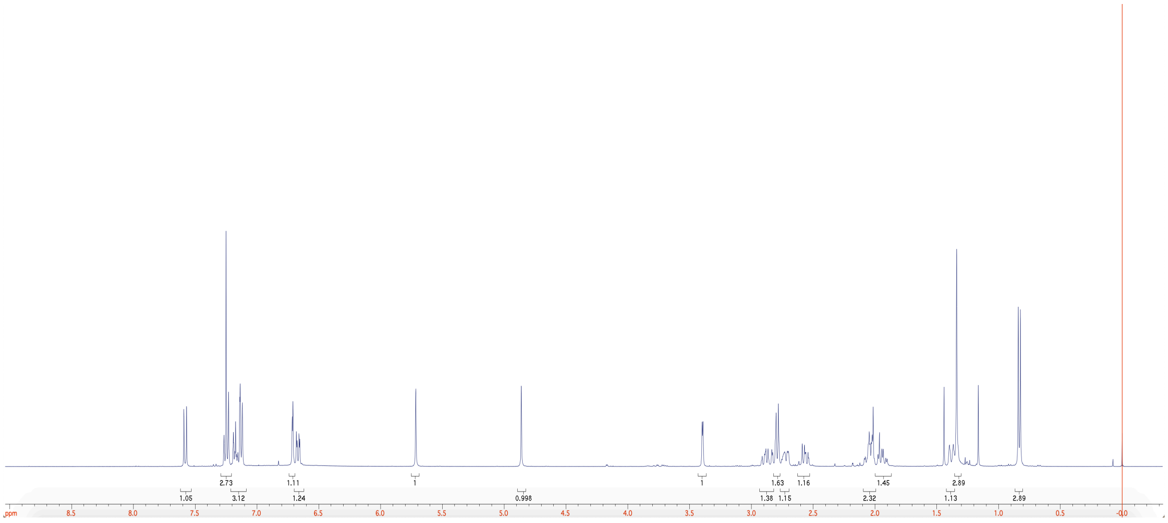

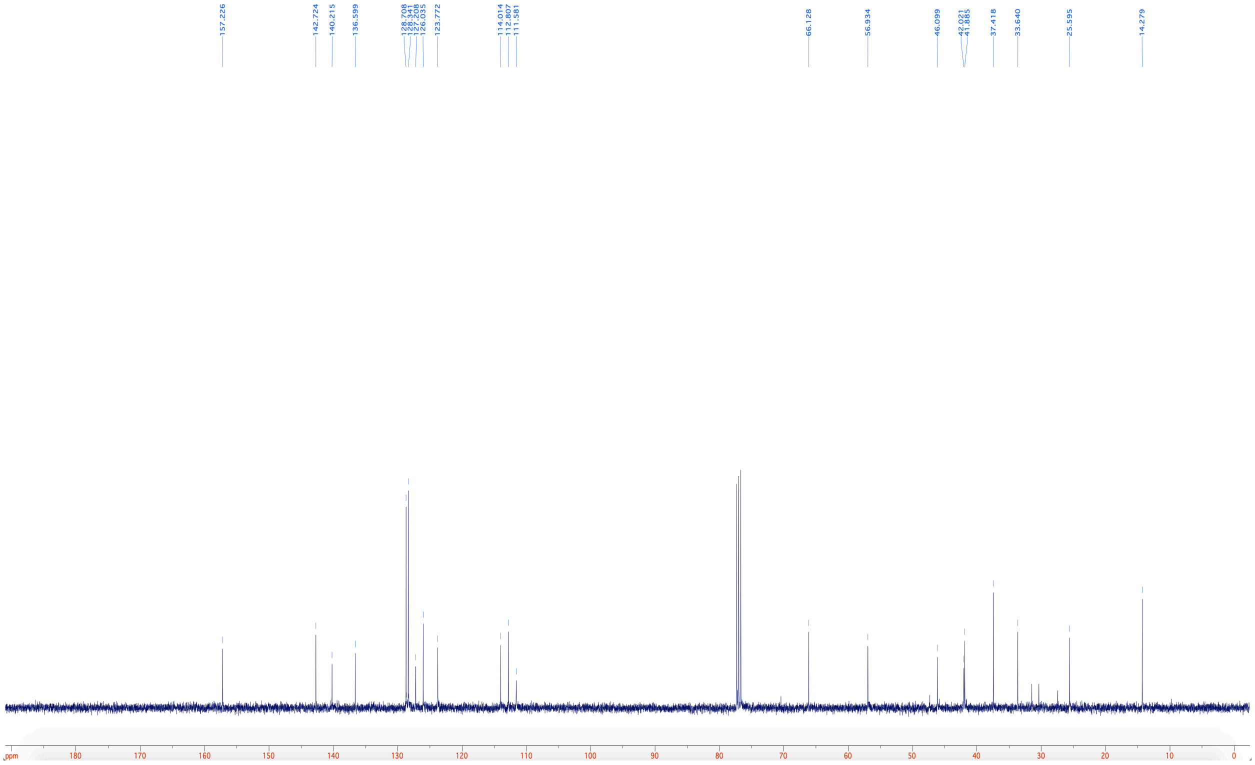

^{1}H NMR and ^{13}C NMR of (+)-38

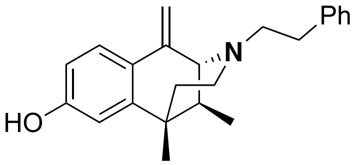

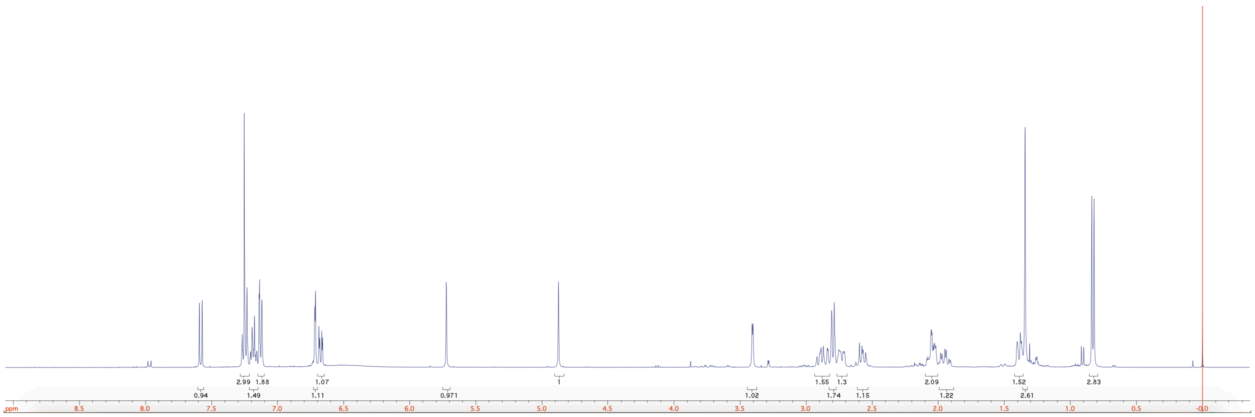

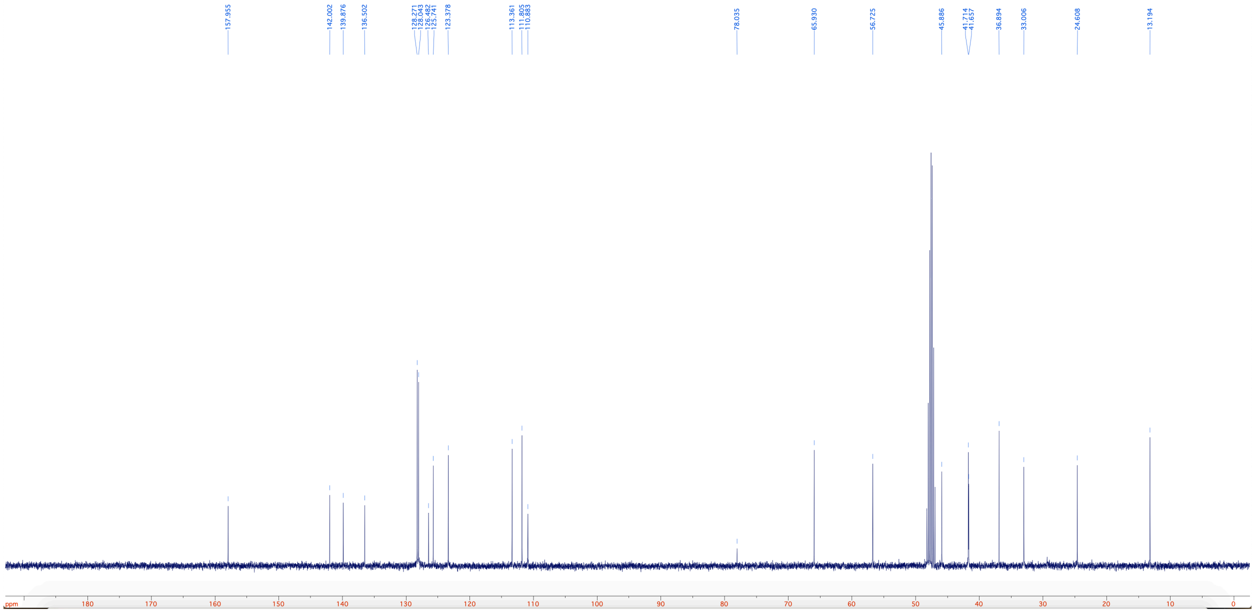

^{1}H NMR and ^{13}C NMR of (–)-39

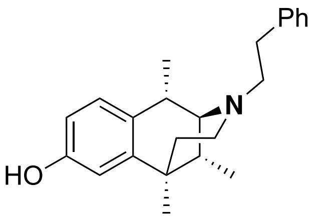

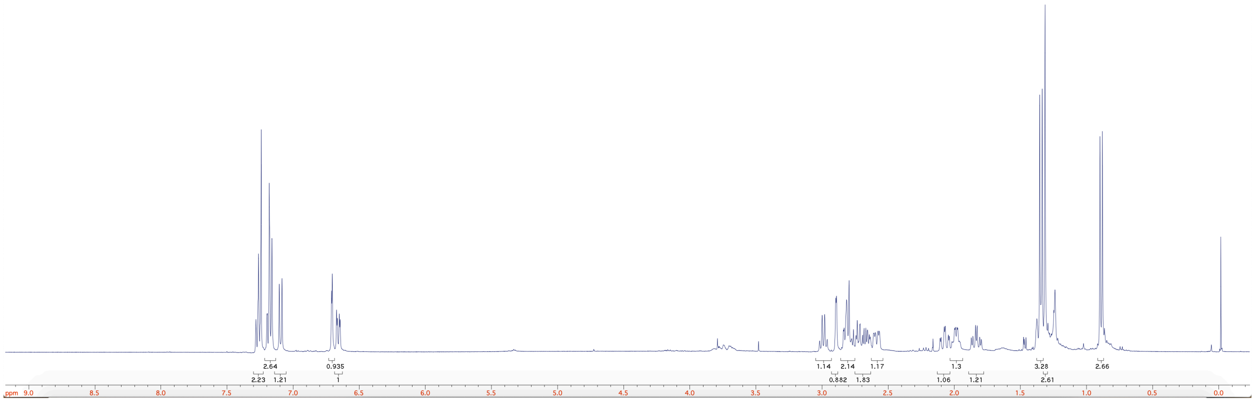

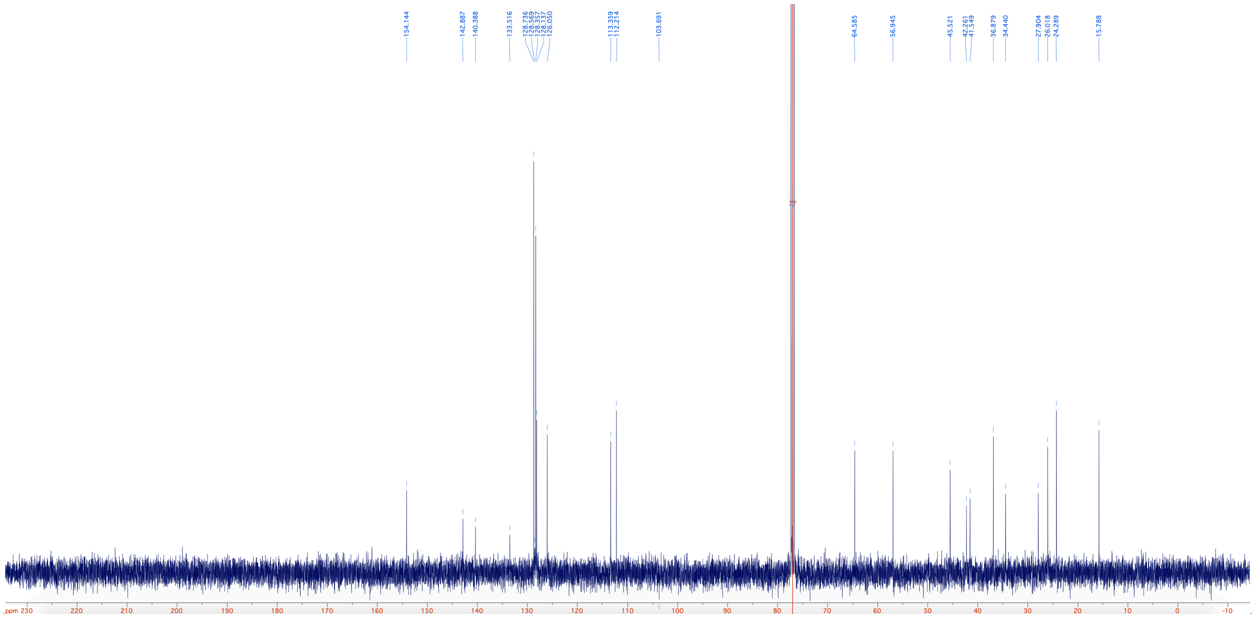

^{1}H NMR and ^{13}C NMR of (+)-39

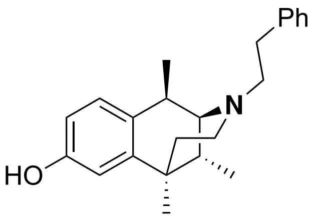

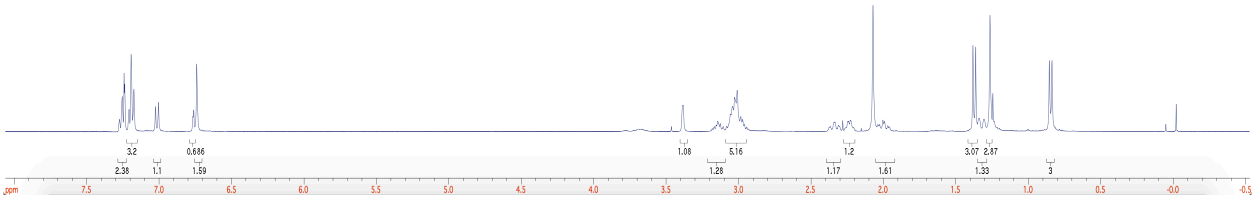

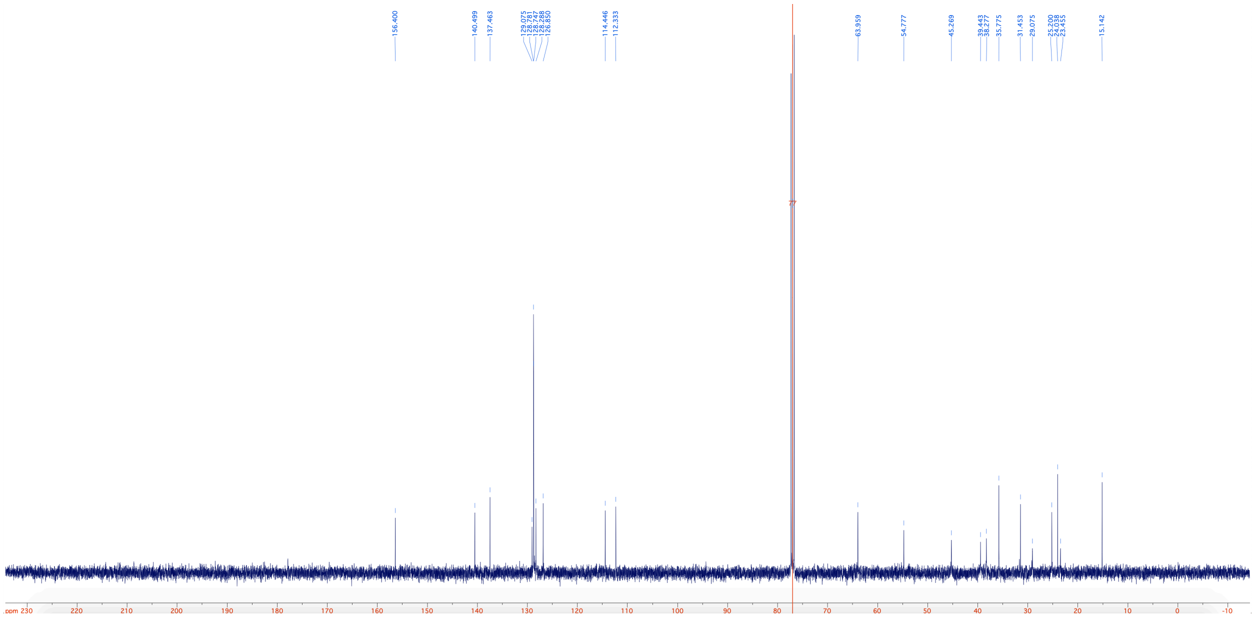

Table S1. Crystal data and structure refinement for (+)-**34**•HBr

Identification code	knih131	
Empirical formula	$C_{24}H_{32}BrNO_3$	
Formula weight	462.41	
Temperature	293(2) K	
Wavelength	1.54178 Å	
Crystal system	Monoclinic	
Space group	$P2_1$	
Unit cell dimensions	a = 11.2668(6) Å	α= 90°.
	b = 7.8053(4) Å	β= 97.890(2)°.
	c = 13.0428(6) Å	γ = 90°.
Volume	1136.13(10) $Å^3$	
Z	2	
Density (20ºC)	1.352 Mg/m^3	
Absorption coefficient	2.652 mm^{-1}	
F(000)	484	
Crystal size	0.097 x 0.074 x 0.060 mm^3	
Theta range for data collection	3.421 to 74.642°.	
Index ranges	-13<=h<=14, -9<=k<=9, -16<=l<=16	
Reflections collected	17096	
Independent reflections	4606 [R_{int} = 0.0970]	
Completeness to theta = 67.679°	100.0 %	
Absorption correction	Semi-empirical from equivalents	
Max. and min. transmission	0.7516 and 0.6310	
Refinement method	Full-matrix least-squares on F^2	
Data / restraints / parameters	4606 / 4 / 272	
Goodness-of-fit on F^2	1.062	
Final R indices [I>2sigma(I)]	R_1 = 0.0573, wR_2 = 0.1474	
R indices (all data)	R_1 = 0.0586, wR_2 = 0.1501	
Absolute structure parameter	-0.02(2)	
Largest diff. peak and hole	1.083 and -0.458 e.$Å^{-3}$	

Table S2. Atomic coordinates (x 10^4) and equivalent isotropic displacement parameters ($Å^2$x 10^3) for (+)-**34**•HBr. U(eq) is defined as one third of the trace of the orthogonalized U^{ij} tensor.

	x	y	z	U(eq)
C(1)	6834(3)	4479(4)	4542(3)	50(1)
N(2)	7036(3)	2914(4)	3901(3)	50(1)
C(3)	7565(3)	1456(5)	4570(3)	50(1)
C(4)	8685(3)	2034(5)	5261(3)	54(1)
C(5)	8512(3)	3661(5)	5894(3)	50(1)
C(6)	7610(3)	3295(5)	6645(3)	51(1)
C(7)	6382(3)	3508(5)	6321(3)	52(1)
O(8)	4856(2)	4285(7)	4968(3)	75(1)
C(8)	5918(3)	4082(6)	5278(3)	54(1)
C(9)	8033(3)	5072(5)	5123(3)	51(1)
C(10)	7868(4)	6833(5)	5607(4)	65(1)
C(11)	9745(3)	4174(9)	6466(4)	68(1)
C(12)	7997(4)	2716(6)	7637(4)	61(1)
O(13)	7498(6)	1897(7)	9322(4)	95(1)
C(13)	7173(5)	2418(6)	8327(4)	70(1)
C(13A)	8747(9)	1691(15)	9693(6)	115(3)
C(14)	5957(5)	2640(7)	8012(4)	73(1)
C(15)	5568(4)	3166(5)	7021(4)	62(1)
C(16)	5940(4)	2305(5)	3202(3)	55(1)
C(17)	5492(6)	3574(8)	2361(5)	86(2)
C(18)	4437(5)	2808(7)	1670(4)	69(1)
C(19)	3289(6)	3006(8)	1901(5)	82(1)
C(20)	2316(7)	2289(14)	1303(9)	116(4)
C(21)	2521(12)	1314(15)	463(9)	124(4)
C(22)	3625(13)	1112(11)	198(6)	125(4)
C(23)	4591(8)	1851(10)	809(5)	91(2)
Br(24)	8999(1)	4191(1)	2459(1)	79(1)
O(25)	7380(5)	7844(11)	2635(6)	117(2)
C(26)	7585(7)	8882(14)	1818(7)	108(3)

Table S3. Bond lengths [Å] and angles [°] for (+)-**34**•HBr.

C(1)-N(2)	1.515(5)	C(1)-C(9)	1.527(5)
C(1)-C(8)	1.535(5)	C(1)-H(1)	0.9800
N(2)-C(3)	1.506(4)	N(2)-C(16)	1.507(5)
N(2)-H(2)	1.02(8)	C(3)-C(4)	1.515(5)
C(3)-H(3A)	0.9700	C(3)-H(3B)	0.9700
C(4)-C(5)	1.541(5)	C(4)-H(4A)	0.9700
C(4)-H(4B)	0.9700	C(5)-C(6)	1.533(5)
C(5)-C(11)	1.536(5)	C(5)-C(9)	1.538(5)
C(6)-C(12)	1.383(6)	C(6)-C(7)	1.399(5)
C(7)-C(15)	1.407(6)	C(7)-C(8)	1.459(6)
O(8)-C(8)	1.220(4)	C(9)-C(10)	1.535(5)
C(9)-H(9)	0.9800	C(10)-H(10A)	0.9600
C(10)-H(10B)	0.9600	C(10)-H(10C)	0.9600
C(11)-H(11A)	0.9600	C(11)-H(11B)	0.9600
C(11)-H(11C)	0.9600	C(12)-C(13)	1.399(7)
C(12)-H(12)	0.9300	O(13)-C(13)	1.361(7)
O(13)-C(13A)	1.433(12)	C(13)-C(14)	1.387(9)
C(13A)-H(13A)	0.9600	C(13A)-H(13B)	0.9600
C(13A)-H(13C)	0.9600	C(14)-C(15)	1.369(8)
C(14)-H(14)	0.9300	C(15)-H(15)	0.9300
C(16)-C(17)	1.512(6)	C(16)-H(16A)	0.9700
C(16)-H(16B)	0.9700	C(17)-C(18)	1.512(7)
C(17)-H(17A)	0.9700	C(17)-H(17B)	0.9700
C(18)-C(19)	1.376(9)	C(18)-C(23)	1.380(10)
C(19)-C(20)	1.375(11)	C(19)-H(19)	0.9300
C(20)-C(21)	1.38(2)	C(20)-H(20)	0.9300
C(21)-C(22)	1.345(19)	C(21)-H(21)	0.9300
C(22)-C(23)	1.384(14)	C(22)-H(22)	0.9300
C(23)-H(23)	0.9300	O(25)-C(26)	1.383(13)
O(25)-H(25)	0.82(3)	C(26)-H(26A)	0.9600
C(26)-H(26B)	0.9600	C(26)-H(26C)	0.9600
N(2)-C(1)-C(9)	109.2(3)	N(2)-C(1)-C(8)	110.3(3)
C(9)-C(1)-C(8)	112.1(3)	N(2)-C(1)-H(1)	108.4
C(9)-C(1)-H(1)	108.4	C(8)-C(1)-H(1)	108.4
C(3)-N(2)-C(16)	109.8(3)	C(3)-N(2)-C(1)	111.6(3)
C(16)-N(2)-C(1)	114.6(3)	C(3)-N(2)-H(2)	109(4)
C(16)-N(2)-H(2)	110(4)	C(1)-N(2)-H(2)	101(4)
N(2)-C(3)-C(4)	110.5(3)	N(2)-C(3)-H(3A)	109.5
C(4)-C(3)-H(3A)	109.5	N(2)-C(3)-H(3B)	109.5
C(4)-C(3)-H(3B)	109.5	H(3A)-C(3)-H(3B)	108.1
C(3)-C(4)-C(5)	114.1(3)	C(3)-C(4)-H(4A)	108.7
C(5)-C(4)-H(4A)	108.7	C(3)-C(4)-H(4B)	108.7
C(5)-C(4)-H(4B)	108.7	H(4A)-C(4)-H(4B)	107.6
C(6)-C(5)-C(11)	111.9(3)	C(6)-C(5)-C(9)	110.3(3)
C(11)-C(5)-C(9)	110.1(3)	C(6)-C(5)-C(4)	109.4(3)
C(11)-C(5)-C(4)	107.6(4)	C(9)-C(5)-C(4)	107.4(3)
C(12)-C(6)-C(7)	119.5(4)	C(12)-C(6)-C(5)	120.5(3)
C(7)-C(6)-C(5)	120.0(4)	C(6)-C(7)-C(15)	119.2(4)
C(6)-C(7)-C(8)	122.0(3)	C(15)-C(7)-C(8)	118.8(4)
O(8)-C(8)-C(7)	124.0(4)	O(8)-C(8)-C(1)	118.7(4)
C(7)-C(8)-C(1)	117.4(3)	C(1)-C(9)-C(10)	109.1(3)
C(1)-C(9)-C(5)	108.3(3)	C(10)-C(9)-C(5)	115.0(4)
C(1)-C(9)-H(9)	108.0	C(10)-C(9)-H(9)	108.0

C(5)-C(9)-H(9)	108.0	C(9)-C(10)-H(10A)	109.5
C(9)-C(10)-H(10B)	109.5	H(10A)-C(10)-H(10B)	109.5
C(9)-C(10)-H(10C)	109.5	H(10A)-C(10)-H(10C)	109.5
H(10B)-C(10)-H(10C)	109.5	C(5)-C(11)-H(11A)	109.5
C(5)-C(11)-H(11B)	109.5	H(11A)-C(11)-H(11B)	109.5
C(5)-C(11)-H(11C)	109.5	H(11A)-C(11)-H(11C)	109.5
H(11B)-C(11)-H(11C)	109.5	C(6)-C(12)-C(13)	120.3(4)
C(6)-C(12)-H(12)	119.9	C(13)-C(12)-H(12)	119.9
C(13)-O(13)-C(13A)	118.6(6)	O(13)-C(13)-C(14)	116.4(5)
O(13)-C(13)-C(12)	123.3(5)	C(14)-C(13)-C(12)	120.3(5)
O(13)-C(13A)-H(13A)	109.5	O(13)-C(13A)-H(13B)	109.5
H(13A)-C(13A)-H(13B)	109.5	O(13)-C(13A)-H(13C)	109.5
H(13A)-C(13A)-H(13C)	109.5	H(13B)-C(13A)-H(13C)	109.5
C(15)-C(14)-C(13)	119.5(4)	C(15)-C(14)-H(14)	120.2
C(13)-C(14)-H(14)	120.2	C(14)-C(15)-C(7)	121.1(4)
C(14)-C(15)-H(15)	119.4	C(7)-C(15)-H(15)	119.4
N(2)-C(16)-C(17)	113.4(3)	N(2)-C(16)-H(16A)	108.9
C(17)-C(16)-H(16A)	108.9	N(2)-C(16)-H(16B)	108.9
C(17)-C(16)-H(16B)	108.9	H(16A)-C(16)-H(16B)	107.7
C(18)-C(17)-C(16)	109.3(4)	C(18)-C(17)-H(17A)	109.8
C(16)-C(17)-H(17A)	109.8	C(18)-C(17)-H(17B)	109.8
C(16)-C(17)-H(17B)	109.8	H(17A)-C(17)-H(17B)	108.3
C(19)-C(18)-C(23)	117.8(6)	C(19)-C(18)-C(17)	120.7(6)
C(23)-C(18)-C(17)	121.5(6)	C(20)-C(19)-C(18)	122.0(8)
C(20)-C(19)-H(19)	119.0	C(18)-C(19)-H(19)	119.0
C(19)-C(20)-C(21)	117.9(10)	C(19)-C(20)-H(20)	121.1
C(21)-C(20)-H(20)	121.1	C(22)-C(21)-C(20)	122.1(8)
C(22)-C(21)-H(21)	119.0	C(20)-C(21)-H(21)	119.0
C(21)-C(22)-C(23)	119.0(9)	C(21)-C(22)-H(22)	120.5
C(23)-C(22)-H(22)	120.5	C(18)-C(23)-C(22)	121.2(9)
C(18)-C(23)-H(23)	119.4	C(22)-C(23)-H(23)	119.4
C(26)-O(25)-H(25)	117(5)	O(25)-C(26)-H(26A)	109.5
O(25)-C(26)-H(26B)	109.5	H(26A)-C(26)-H(26B)	109.5
O(25)-C(26)-H(26C)	109.5	H(26A)-C(26)-H(26C)	109.5
H(26B)-C(26)-H(26C)	109.5		

Table S4. Anisotropic displacement parameters ($Å^2$x 10^3) for (+)-**34•**HBr. The anisotropic displacement factor exponent takes the form: $-2\pi^2[h^2a^{*2}U^{11} + ... + 2 h k a^* b^* U^{12}]$

	U^{11}	U^{22}	U^{33}	U^{23}	U^{13}	U^{12}
C(1)	50(1)	39(2)	61(2)	2(1)	10(1)	2(1)
N(2)	49(1)	42(1)	58(2)	4(1)	11(1)	2(1)
C(3)	53(2)	40(1)	60(2)	3(1)	13(1)	7(1)
C(4)	45(2)	50(2)	70(2)	5(2)	14(2)	6(1)
C(5)	41(1)	48(2)	61(2)	2(1)	9(1)	-2(1)
C(6)	51(2)	45(2)	58(2)	-1(1)	14(1)	-4(1)
C(7)	51(2)	43(1)	65(2)	-5(1)	19(2)	-2(1)
O(8)	46(1)	94(2)	85(2)	-8(2)	9(1)	12(2)
C(8)	43(1)	49(2)	71(2)	-7(2)	12(1)	3(2)
C(9)	50(2)	42(2)	65(2)	5(1)	14(1)	-3(1)
C(10)	71(2)	46(2)	79(3)	-4(2)	10(2)	-2(2)
C(11)	48(2)	73(2)	82(2)	12(3)	2(2)	-10(2)
C(12)	64(2)	58(2)	62(2)	1(2)	9(2)	-6(2)
O(13)	131(4)	93(3)	65(2)	11(2)	27(2)	-6(3)
C(13)	99(3)	55(2)	60(2)	0(2)	24(2)	-9(2)
C(13A)	142(7)	126(7)	70(3)	22(4)	-6(4)	-6(5)
C(14)	89(3)	64(2)	73(3)	-10(2)	41(2)	-15(2)
C(15)	58(2)	51(2)	83(3)	-11(2)	29(2)	-6(2)
C(16)	59(2)	47(2)	58(2)	1(1)	6(2)	-1(1)
C(17)	95(3)	69(3)	87(3)	27(2)	-19(3)	-16(2)
C(18)	81(3)	60(2)	64(2)	14(2)	-1(2)	-4(2)
C(19)	90(3)	74(3)	80(3)	16(2)	13(2)	-3(2)
C(20)	80(3)	118(6)	141(8)	61(6)	-11(4)	-21(4)
C(21)	150(8)	109(6)	98(6)	25(5)	-40(6)	-43(6)
C(22)	220(12)	84(4)	61(3)	0(3)	-15(5)	-6(6)
C(23)	118(4)	87(4)	68(3)	9(3)	8(3)	12(3)
Br(24)	83(1)	86(1)	74(1)	2(1)	30(1)	-8(1)
O(25)	80(3)	140(5)	136(5)	-5(4)	38(3)	7(3)
C(26)	99(4)	124(8)	97(4)	-28(5)	-3(3)	31(5)

Table S5. Hydrogen coordinates (x10^4) and isotropic displacement parameters (Å2x10^3) for (+)-**34**•HBr.

	x	y	z	U(eq)
H(1)	6512	5397	4071	60
H(2)	7660(60)	3360(100)	3480(60)	74
H(3A)	7762	519	4134	60
H(3B)	6981	1044	4993	60
H(4A)	9311	2246	4835	65
H(4B)	8955	1112	5735	65
H(9)	8597	5202	4619	62
H(10A)	7303	6742	6092	98
H(10B)	7576	7634	5073	98
H(10C)	8624	7225	5959	98
H(11A)	9649	5083	6943	102
H(11B)	10248	4555	5974	102
H(11C)	10107	3203	6838	102
H(12)	8809	2524	7845	74
H(13A)	9064	762	9331	172
H(13B)	8847	1444	10421	172
H(13C)	9166	2728	9575	172
H(14)	5409	2433	8470	87
H(15)	4751	3299	6808	74
H(16A)	5306	2082	3617	66
H(16B)	6127	1233	2883	66
H(17A)	5250	4626	2669	104
H(17B)	6127	3842	1955	104
H(19)	3169	3645	2480	98
H(20)	1543	2455	1460	139
H(21)	1877	780	67	149
H(22)	3737	485	-388	150
H(23)	5358	1699	636	110
H(25)	7700(130)	6900(90)	2680(90)	175
H(26A)	7957	9928	2081	162
H(26B)	6837	9137	1399	162
H(26C)	8103	8300	1406	162

Table S6. Torsion angles [°] for (+)-**34•**HBr.

C(9)-C(1)-N(2)-C(3)	60.5(4)	C(8)-C(1)-N(2)-C(3)	-63.0(3)
C(9)-C(1)-N(2)-C(16)	-174.0(3)	C(8)-C(1)-N(2)-C(16)	62.5(4)
C(16)-N(2)-C(3)-C(4)	178.7(3)	C(1)-N(2)-C(3)-C(4)	-53.2(4)
N(2)-C(3)-C(4)-C(5)	52.1(4)	C(3)-C(4)-C(5)-C(6)	63.7(4)
C(3)-C(4)-C(5)-C(11)	-174.6(4)	C(3)-C(4)-C(5)-C(9)	-56.0(4)
C(11)-C(5)-C(6)-C(12)	-28.0(5)	C(9)-C(5)-C(6)-C(12)	-151.0(4)
C(4)-C(5)-C(6)-C(12)	91.1(4)	C(11)-C(5)-C(6)-C(7)	153.5(4)
C(9)-C(5)-C(6)-C(7)	30.6(5)	C(4)-C(5)-C(6)-C(7)	-87.3(4)
C(12)-C(6)-C(7)-C(15)	1.5(6)	C(5)-C(6)-C(7)-C(15)	180.0(3)
C(12)-C(6)-C(7)-C(8)	-178.7(4)	C(5)-C(6)-C(7)-C(8)	-0.3(6)
C(6)-C(7)-C(8)-O(8)	-179.9(5)	C(15)-C(7)-C(8)-O(8)	-0.1(7)
C(6)-C(7)-C(8)-C(1)	-1.0(6)	C(15)-C(7)-C(8)-C(1)	178.8(3)
N(2)-C(1)-C(8)-O(8)	-87.9(5)	C(9)-C(1)-C(8)-O(8)	150.2(4)
N(2)-C(1)-C(8)-C(7)	93.1(4)	C(9)-C(1)-C(8)-C(7)	-28.8(5)
N(2)-C(1)-C(9)-C(10)	169.6(3)	C(8)-C(1)-C(9)-C(10)	-67.9(4)
N(2)-C(1)-C(9)-C(5)	-64.5(4)	C(8)-C(1)-C(9)-C(5)	58.0(4)
C(6)-C(5)-C(9)-C(1)	-58.3(4)	C(11)-C(5)-C(9)-C(1)	177.7(4)
C(4)-C(5)-C(9)-C(1)	60.8(4)	C(6)-C(5)-C(9)-C(10)	64.1(4)
C(11)-C(5)-C(9)-C(10)	-59.9(5)	C(4)-C(5)-C(9)-C(10)	-176.8(3)
C(7)-C(6)-C(12)-C(13)	-2.9(6)	C(5)-C(6)-C(12)-C(13)	178.6(4)
C(13A)-O(13)-C(13)-C(14)	-178.5(7)	C(13A)-O(13)-C(13)-C(12)	1.9(10)
C(6)-C(12)-C(13)-O(13)	-178.0(5)	C(6)-C(12)-C(13)-C(14)	2.4(7)
O(13)-C(13)-C(14)-C(15)	179.9(5)	(12)-C(13)-C(14)-C(15)	-0.5(8)
C(13)-C(14)-C(15)-C(7)	-0.9(7)	C(6)-C(7)-C(15)-C(14)	0.4(6)
C(8)-C(7)-C(15)-C(14)	-179.4(4)	C(3)-N(2)-C(16)-C(17)	-168.6(5)
C(1)-N(2)-C(16)-C(17)	64.9(6)	N(2)-C(16)-C(17)-C(18)	177.0(5)
C(16)-C(17)-C(18)-C(19)	88.3(7)	C(16)-C(17)-C(18)-C(23)	-89.7(7)
C(23)-C(18)-C(19)-C(20)	-0.2(9)	C(17)-C(18)-C(19)-C(20)	-178.3(6)
C(18)-C(19)-C(20)-C(21)	1.5(10)	C(19)-C(20)-C(21)-C(22)	-2.7(13)
C(20)-C(21)-C(22)-C(23)	2.5(14)	C(19)-C(18)-C(23)-C(22)	0.0(9)
C(17)-C(18)-C(23)-C(22)	178.1(6)	C(21)-C(22)-C(23)-C(18)	-1.1(12)

Table S7. Hydrogen bonds for (+)-**34**•HBr [Å and °].

D-H...A	d(D-H)	d(H...A)	d(D...A)	<(DHA)
N(2)-H(2)...Br(24)	1.02(8)	2.24(8)	3.251(3)	176(6)
C(3)-H(3B)...O(8)#1	0.97	2.49	3.336(5)	145.6
C(9)-H(9)...Br(24)	0.98	3.02	3.843(4)	142.4
C(16)-H(16A)...O(8)	0.97	2.56	3.155(6)	119.6
O(25)-H(25)...Br(24)	0.82(3)	2.61(4)	3.410(7)	166(7)

Symmetry transformations used to generate equivalent atoms:
#1 -x+1,y-1/2,-z+1
